# Novel application of SANS provides quantitative non-destructive identification of forming techniques in late Roman and early medieval pottery from Pannonia

**DOI:** 10.1038/s41598-024-77426-2

**Published:** 2024-10-29

**Authors:** John Gait, Katalin Bajnok, Nicolas Hugot, Friderika Horváth, Gérard Pépy, Darren Ellis, Adél Len

**Affiliations:** 1 Neutron Spectroscopy Department, HUN-REN Centre for Energy Research, Budapest, Hungary; 2 Department of Scientific Research, The British Museum, London, UK; 3 Institute of Archaeological Sciences, Eötvös Loránd University, Budapest, Hungary; 4 ENSTA Paris, Institut Polytechnique de Paris, Palaiseau, France; 5 Institute of Archaeology, HUN-REN Research Centre for Humanities, Budapest, Hungary; 6 Paris, France; 7 Institute of Making, University College London, London, UK; 8 Faculty of Engineering and Information Technology, University of Pécs, Pécs, Hungary

**Keywords:** Pottery technology, SANS, Orientation analysis, Experimental archaeology, Late Roman Pannonia, Nanoscale materials, Characterization and analytical techniques, Archaeology

## Abstract

Within archaeological studies of ancient pottery, understanding the techniques used to form vessels from unfired clay provides significant information on the history of technology and economic systems, as well as wider cultural practices and social interactions. We introduce here a new analytical methodology, using small-angle neutron scattering (SANS) to investigate pottery forming techniques through the preferential orientation of nanoscale objects within pottery fabrics. Significantly, SANS is non-destructive, suitable for both coarse and fine-textured pottery fabrics, provides quantitative data, enables fast-throughput of samples, and is not significantly affected by surface modifications occurring after the primary forming stage. The use of SANS is systematically investigated through over 400 measurements of experimental vessels, and also compared with X-ray microtomography and neutron tomography. The results show that SANS can be used to differentiate wheel-throwing, coil-building, percussion-building, and coil-wheeling techniques. The archaeological application of SANS is demonstrated through a case study of 50 late Roman and early medieval (fourth–sixth century AD) pottery sherds from Hungary, spanning the collapse of the Western Roman Empire and the arrival of Barbarian polities into the region. The findings show a transition in production from predominantly wheel-throwing to coil-wheeling, but also coil-building, percussion-building, percussion-wheeling, and drawing. Such changes appear to reflect the disintegration of large-scale centrally organised Roman economic systems, and the diversification of production, consistent with the more diversified technological and cultural backgrounds of the producers themselves.

## Introduction

Pottery represents one of the most abundant forms of human material culture since its earliest attested appearance in the Late Pleistocene^1,2^. As such, it plays a significant role, either directly or indirectly, in archaeological interpretations of the past across a multiplicity of fields, including stylistic studies and the dating of archaeological sites, subsistence strategies and economics, resource exploitation and environmental interactions, as well as ancient technological practices^3–5^. Within archaeological pottery studies, the production of pottery may be considered as a sequence of discrete technologically distinct stages, from raw material procurement and processing, to forming, firing, and post-firing modifications. Of these, the techniques with which the unfired clay-rich paste is manipulated to form a vessel (e.g. wheel-throwing, coil-building etc.^6^) are of especial significance as they reflect not only the immediate physical requirements of the raw material and intended vessel shape, but also subjective choices made by individual potters in response to various technical, economic, social, cultural, or environmental factors, whether real or perceived^4,7–9^.

### Methodological approaches to the identification of ancient pottery forming techniques

Despite recent developments in many specific areas of scientific archaeological pottery studies^10–13^, analysis of pottery forming techniques still frequently poses significant challenges^14^, and largely relies on the visual observation of macroscopic features (e.g. coil-joins, fracture patterns, variations in wall thickness, surfaces rilling marks and finger impressions etc.^15^). Such features may be hidden or destroyed by later stages of production (e.g. by burnishing, painting, etc.), or lost through post-depositional abrasion, leading to diagnostic features being identified for only a small proportion of some archaeological pottery assemblages, particularly assemblages of fragmentary and abraded sherds^16,17^. In addition, some forming techniques tend to result in more distinctive macroscopic features than others, potentially leading to biases in their identification within the archaeological record. In this regard, the great extent to which coil-building is reported within many archaeological pottery assemblages, may be due in no small part to the comparative ease with which it may be identified from the joins between successive layers of coils^5^. These joins may, in some instances, be observed in thin sections or radiographs as large horizontal, elongated voids, or inferred in hand-specimens from characteristic horizontal fractures, and, significantly, may remain after subsequent surface treatments (although recent experimental studies have questioned the extent to which coil-joins may in fact be preserved^18^). By contrast, the identification of moulding or drawing, for example, commonly rely on more subtle and less durable features such as surface indentations, potentially leading to the under-detection of these forming techniques^5^.

Forming techniques have also been inferred from the microstructure of pottery fabrics, in particular from the orientation of elongated voids, aplastic inclusions and clay domains^6,14,19–22^. Such microstructural information is most commonly obtained through visual inspection of fresh breaks^23^, or optical microscopy of thin sections, both of which require destructive sampling. Microstructural information may also be gained from X-ray radiography^19,24–26^ and, increasingly, tomography^18,27–29^. Although these techniques offer non-destructive alternatives to thin sections, their effectiveness depends largely on the composition of the pottery fabrics and, in particular, the frequency of the objects (i.e. particles and voids) observed within the fabrics. For all microstructural approaches, instrumental limitations in spatial resolution, and hence the minimum size of objects that may be resolved, are also significant, thereby tending to favour the analysis of coarse-textured wares with sand-sized inclusions or voids (i.e. > 63 µm).

A further difficulty is the predominantly qualitative and subjective nature of many conventional analytical approaches to the investigation of forming techniques. This applies particularly to macroscopic studies, where purportedly diagnostic features are typically illustrated with annotated photographs of a small number of vessels or sherds. In such cases, it is difficult to independently scrutinise and re-evaluate the entire data from a study, as not only are the highlighted features prone to subjective interpretations, but also it is not practicable to illustrate every sample studied. Microstructural approaches potentially offer some advantages in this regard as, in principle, the orientation of objects within the fabric can be quantified^18,30^ and therefore objectively characterised. Nonetheless, in practice, the evaluation of the orientation of objects in thin sections or X-ray radiographs is often undertaken in an entirely qualitative manner^14,31^, or relies on the manual, and subjective, selection of individual objects for measurement^32^, or else is presented without adequate statistical information, such as the frequency of objects analysed. Consequently, in the absence of objective and quantitative analytical data it is often difficult to compare information from different archaeological pottery assemblages or from different research teams.

### Differentiating wheel-throwing and ‘composite’ forming techniques

The use of wheel-throwing has received particular attention within various regions on account of its potential direct economic impact, arising from increased speed and standardisation of production, as well as the more indirect social effects associated with craft specialisation and the regional and temporal dissemination of technological knowledge^8,33–40^. However, as wheel-throwing tends to favour the use of fine-textured fabrics containing small, often equant, inclusions, it is often difficult to identify within the archaeological record using microstructural approaches^14^. With regard to macroscopic traces, identification is frequently complicated by the practice of secondary ‘wheel-shaping’, where vessels that have been initially formed using a hand-building technique have subsequently been refined by shaping or smoothing on a wheel, resulting in surface traces that may be difficult to distinguish from those left by wheel-throwing.

The combination of primary hand-building techniques with secondary wheel-shaping has also been a particular focus of research^41–45^, and such composite techniques may be seen as important steps in the gradual assimilation of wheel-throwing, or as distinct technological practices in themselves. However, investigations have tended to concentrate on the specific combination of coil-building followed by modification on a wheel; so-called ‘wheel-coiling’ or simply ‘wheel-fashioning’^40,41,45^, although here the term ‘coil-wheeling’ is proposed instead, in order to reflect the sequence and types of forming actions (see also Supplementary Material A, Note 3 for clarification of technical terms used here). By contrast, the potential use of other primary hand-building techniques, such as various percussion-building techniques or drawing, prior to secondary wheel-shaping, has largely been ignored. While such alternative combinations have, occasionally, been suggested^39,46^, their use is difficult to confirm by existing conventional macroscopic or microstructural approaches. Nonetheless, the use of hand-building techniques other than coil-building in combination with secondary use of a wheel, should be examined in more detail and considered as potentially plausible alternatives, not least because of the relative speed with which vessels may be formed initially using percussion-building or drawing.

Accordingly, the present study attempts to redress some of these difficulties in the analysis of ancient pottery forming techniques, by describing a novel, non-destructive and quantitative analytical approach, combining the analysis of the orientation nanoscale objects (crystallites, grains, pores) together with the observation of macroscopic features. This approach offers the potential to differentiate a range of forming techniques, including coil-wheeling and other composite techniques, whilst avoiding existing intrinsic biases in identification, and which may be applied to either coarse or fine textured pottery fabrics. Following detailed experimental studies, the utility of this analytical approach is demonstrated with an archaeological case study examining fourth–sixth century AD pottery from Hungary. Here, the disintegration of the centralised Roman administration followed by the decline of large-scale ‘industrial’ pottery workshops is often linked to the reduction in the occurrence of wheel-thrown pottery, and, implicitly, to the disappearance of the Roman lifestyle and the emergence of early medieval barbarian polities^47^. From a wider perspective, this case study also hopes to illustrate that while the introduction of wheel-throwing is often the focus of attention, its apparent decline may also be a rewarding field of study.

### Using SANS for analysing the orientation of nanoscale particles and voids

Some of the specific difficulties encountered in macroscopic and microstructural studies of pottery forming techniques may be resolved through the use of small-angle neutron scattering (SANS). SANS is a well-established, non-destructive, material science analytical technique that uses the coherent and elastic scattering of incident neutrons by the nuclei of a target sample^48^ to investigate a range of structural properties of materials down to the nanometric scale.

As SANS is a non-destructive method, usually requiring no prior sample preparation, it is suitable for the study of a range of cultural heritage artefacts^49^. With regard to archaeological ceramics, SANS has previously been used to investigate the porosity and firing temperatures of pottery^49–55^ and clay bricks^56,57^, but as far as the present authors are aware, SANS has not previously been used to investigate pottery forming techniques.

During a SANS measurement, a sample is moved into the path of a moderated and collimated neutron beam, originating from a nuclear reactor or equivalent neutron source. As neutrons are electrically neutral particles, they are able to penetrate a few hundreds of micrometres to several millimetres of material, depending on the composition of the sample^49,58^, before multiple scattering starts to have a potentially detrimental effect on SANS measurements. For pottery, while samples of up to 8 mm in thickness have previously been measured with SANS^49^, the maximum suitable thickness has not yet been determined. Notwithstanding this uncertainty, SANS offers the potential to provide structural information averaged from the entire volume of a sample within the neutron beam, and not from just the surface. For the analysis of forming techniques, this has the advantage that structural features located in the core of a sample, produced during primary forming and not altered by subsequent surface modifications, may provide the predominant component of the overall neutron scattering during measurements.

Coherent neutron scattering occurs if spatially correlated atomic positions form ‘scattering domains’, which are defined as regions of uniform scattering length densities (determined by elemental/isotopic composition and bulk density) that differ from their surroundings in scattering length density^59^. Such scattering domains, ranging in length between c. 10 to a few 100 nm, may represent particles, pores or crystallites in their entirety, or partial regions of larger objects. Significantly, although the scattering domains themselves fall within the nanometre scale, domains forming regions of larger objects also contribute to the overall coherent scattering detected, therefore, the behaviour of particles with lengths of tenths of micrometres to millimetres also influence SANS measurements.

Pottery fabrics typically consist of greater than 50 vol% matrix, interspersed with variable amounts of aplastic inclusions (mineral/rock/ceramic particles, organic objects) and voids (pores, relics of thermally or chemically decomposed inclusions, or structural defects) greater than c. 10 µm in diameter. The matrix itself, in addition to smaller inclusions and voids, consists primarily of various types of hydrous aluminium silicate clay minerals (generally kaolinite, illite, or smectite), appearing as platy crystals of less than 2 µm diameter, in varying states of sintering or vitrification depending on the composition of the fabric and the temperature and atmosphere during firing^14^ (Supplementary Material A Figures S4 & S5). The majority of the neutron scattering may be assumed to result from the interfaces of the scattering domains within the matrix, rather than from the interfaces of larger inclusions or voids, and therefore, as a clay-rich matrix is a necessary component of all pottery fabrics, SANS may be used to investigate the structure of both fine- and coarse-textured pottery.

The neutrons scattered at angles below approximately 10° are recorded by a 2D position sensitive detector, and may display isotropic or anisotropic scattering. The measured intensity distribution of the scattered neutrons is governed by the 3D geometrical properties of the scattering domains, namely the curvature of the interfaces exposed to neutrons, and their spatial arrangement in the sample volume^60,61^. Accordingly, the neutron scattering intensity measured in any given 2D plane, will also be affected by the preferential alignment of the scattering domains within a sample. In practice, for a simple material system consisting of two phases, the scattering intensity distribution may provide information on both the orientation and shape of the domains, however, for materials with many phases and domains of more than one typical shape, and with a wide size distribution, reconstruction of the shape of the scattering domains becomes complex, or even impossible ^60,61^. Nonetheless, for such complex materials, more limited information on the average orientation and elongation of domains in a sample may still be obtained from the two-dimensional evaluation of the scattering intensity distribution^62,63^.

**Fig. 1:**
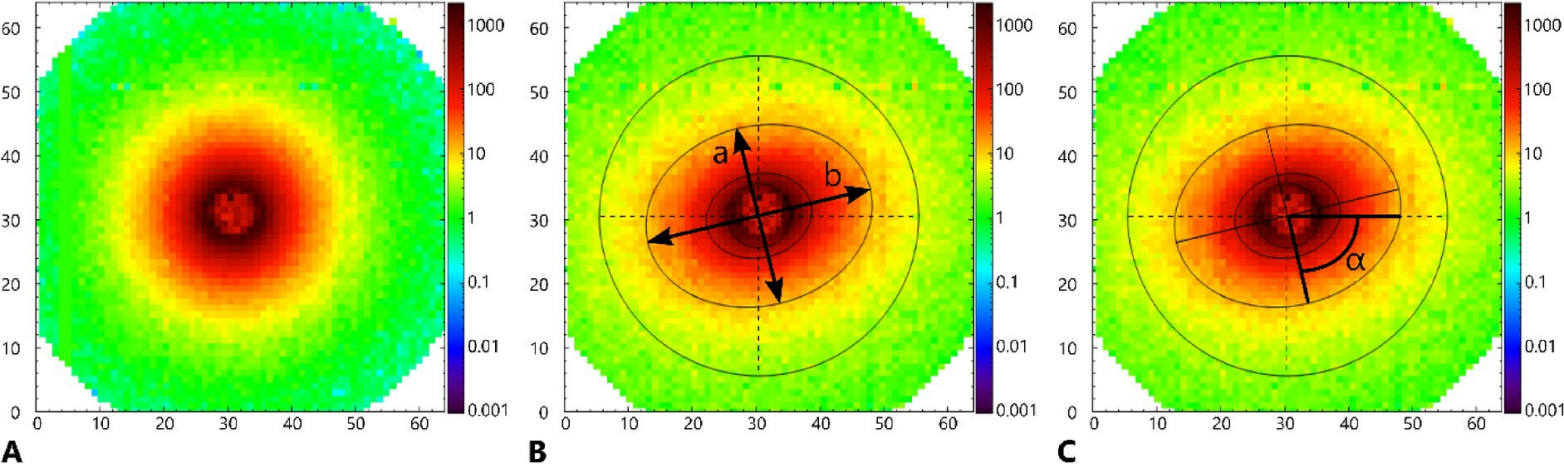
SANS 2D neutron scattering intensity distribution diagrams. **A** Isotropic scattering; **B** anisotropic neutron scattering, with the ‘isotropy’ of the interfaces of the scattering domains, *I*, calculated as the ratio of the lengths of the minor and major axes of the fitted ellipse (*I* = a/b where a ≤ b, 0 < *I* ≤ 1); **C** mean tilting angle, *α*, of interfaces of scattering domains measured as the angle from the positive horizontal axis to the nearest minor axis of the ellipse fitted to the neutron scattering intensity distribution (-90° < *α* ≤ 90°).

For the investigation of pottery forming techniques, two specific parameters of 2D SANS measurements may be utilised: ‘isotropy’ (0 < *I* ≤ 1), and ‘tilting-angle’ (-90° < *α* ≤ 90°). The 2D scattered neutron intensity distribution recorded by the SANS detector is a Fourier transformation of the real space neutron scattering length density distribution within the entire in-beam volume of the sample. For a material in which the scattering domains are randomly orientated, or are spherical, the recorded intensity distribution of the scattered neutrons will be circularly symmetric around the centre of the beam (isotropic scattering, *I* = 1) (Fig. 1A). For a material in which the scattering domains are non-spherical and have a preferential orientation, the intensity of the scattered neutrons varies with the direction of the scattering vector. The scattering vector is defined as the difference between the wave vectors of the incident and scattered neutrons. In these cases, the 2D scattering patterns on the detector often appear elliptical or show other forms of asymmetry, indicating that the structural properties of the sample differ along different directions (anisotropic scattering, *I* < 1). For example, the direction of the axis of greatest elongation for an elliptical scattering pattern (Fig. 1B), will be orientated perpendicular to that of the axis of the average alignment of the scattering domains (because of the reciprocal space – real space conversion). The magnitude of the isotropy of the neutron scattering intensity distribution, and hence also the isotropy of the scattering domain interfaces, is given by the ratio of the smallest and largest deviations from an isotropic scattering case. If the recorded neutron scattering intensity distribution is approximated as an ellipse (Fig. 1B), the isotropy will be given by the ratio of the lengths of the minor and major axes of the ellipse.

For samples displaying anisotropic scattering, the ‘tilting angle’ is defined as the mean angle of direction of the interfaces of the scattering domains, measured in degrees from a plane parallel to the rim or exterior wall of a vessel. In practice, the tilting angle is calculated as the angle from the horizontal axis of the scattering intensity diagram to the minor axis of an ellipse approximating the intensity distribution of the neutron scattering (Fig. 1C). For samples showing isotropic, or near-isotropic scattering (i.e. *I* ≥ c. 0.95), the accuracy of the tilting angle determined from a fitted ellipse may be reduced.

The curvature of the interfaces of the scattering domains ‘seen’ by the neutrons depends on the orientation of the scattering domains relative to the direction of the neutron beam. Accordingly, in order to determine the average 3D orientation of the scattering domains using 2D SANS measurements, it is necessary to measure in more than one plane. However, if certain assumptions may be made regarding limitations in the degrees of freedom of the orientation of the scattering domains, then their orientation may be ascertained from fewer measurements.

## Experimental investigations

### Measurement conditions and primary data processing

In order to investigate the application of SANS for the identification of pottery forming techniques, a series of experiments were performed at the YS-SANS instrument at the Budapest Neutron Centre^49^. This instrument is a pin-hole type, medium flux (~ 5 × 10^7^ n/cm^2^/s at 5 Å neutron wavelength) instrument, equipped with a 64 × 64 pixel position-sensitive detector, with 1 × 1 cm pixel size. The experimental setup was chosen to match the high scattering vector region (0.01–0.1 Å^-1^), where the scattering from the interfaces (power-law type scattering) dominates over the scattering described by geometrical models (form factors). The large Q region was set by neutron wavelengths between 4 and 5 Å, and a sample-to-detector distance of 5.3 m. The beam size was set from 2 to 16 mm according to experimental requirements, and individual measurements took between c. 5 and 15 min depending on the sample and beam diameter. For all measurements, the raw SANS neutron intensity distributions were first calibrated following the standard calibration procedure^64^ and then processed using the purpose-written 2D SANS evaluation software ‘NSXY v.2022’ (Supplementary Material A Note 1). The main advantage of this software is the fast data processing (hundreds of measurements per minute). As an output of the software, isotropy values and tilting angles were obtained. Where necessary, the resulting tilting angles were mathematically corrected to compensate for any slope of the wall of the sample relative to the beam at each measurement location (Supplementary Material A Note 2).

### Experimental materials

**Fig. 2:**
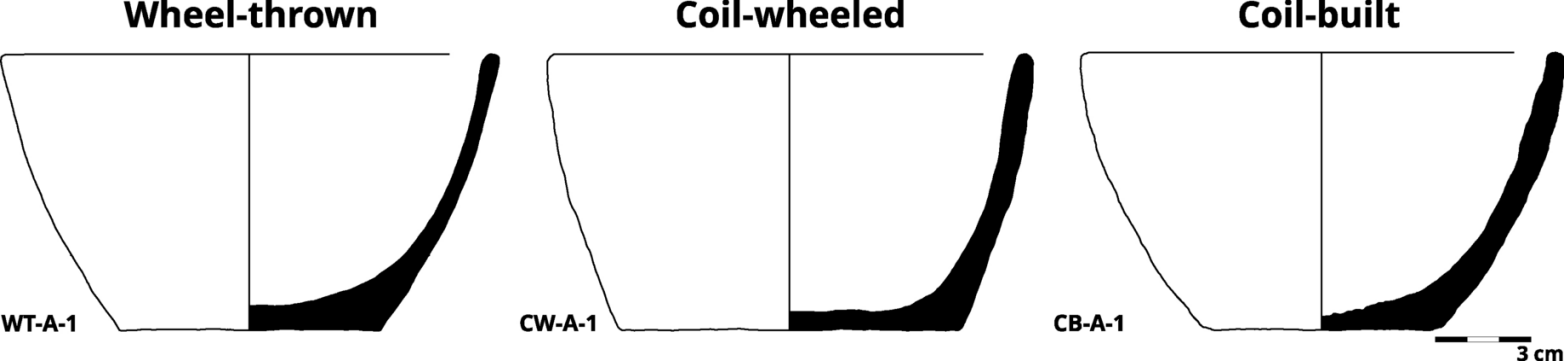
Profiles of representative experimental vessels (see also Supplementary Material A Figure S6).

The initial sets of experimental samples were made using two medium/fine-textured fabrics (A and B) and three forming techniques to produce simple, open-shaped bowls (Fig. 2; Supplementary Material A Note 3 and Figures S2 & S3). The initial forming techniques used were wheel-throwing, coil-building, and coil-wheeling (i.e. coil-building followed by secondary shaping on a wheel). Three vessels were made of each combination of fabric and forming technique. After forming and drying, the vessels were cut vertically into quarters, and each quarter was then fired at either 650, 800, or 950 °C, with one quarter left unfired. Sections of coils used in the coil-building technique were also analysed separately from the completed coil-built vessels.

In addition, samples from nine experimental vessels and coil-sections from an earlier study^18^, made from a coarse, calcareous, organic tempered fabric (designated here as Fabric C; Supplementary Material A Figure S3) using coil-building and tamper-and-concave-anvil percussion-building ^65^, were also measured with SANS (Table 1).

**Table 1:** Summary of experimental samples. WT (Wheel-thrown), CW (Coil-wheeled), CB (Coil-built), Co (Coil-sections), PB (Percussion-built). See Supplementary Material A Note 3 for further information.

Fabric	Forming techniques	Firing temperature (°C)	Number of samples
A	WT, CW, CB, Co	650, 800, 950	36
B	WT, CW, CB, Co	650, 800, 950	36
C	PB, CB, Co	700	9

### Measurement strategy

The measurement of the experimental samples was divided into three stages to investigate a range of variables and to determine the optimal parameters for differentiating forming techniques. The optimal measurement parameters determined in each stage were then implemented in subsequent stages.

#### 3D orientation and temperature

**Fig. 3:**
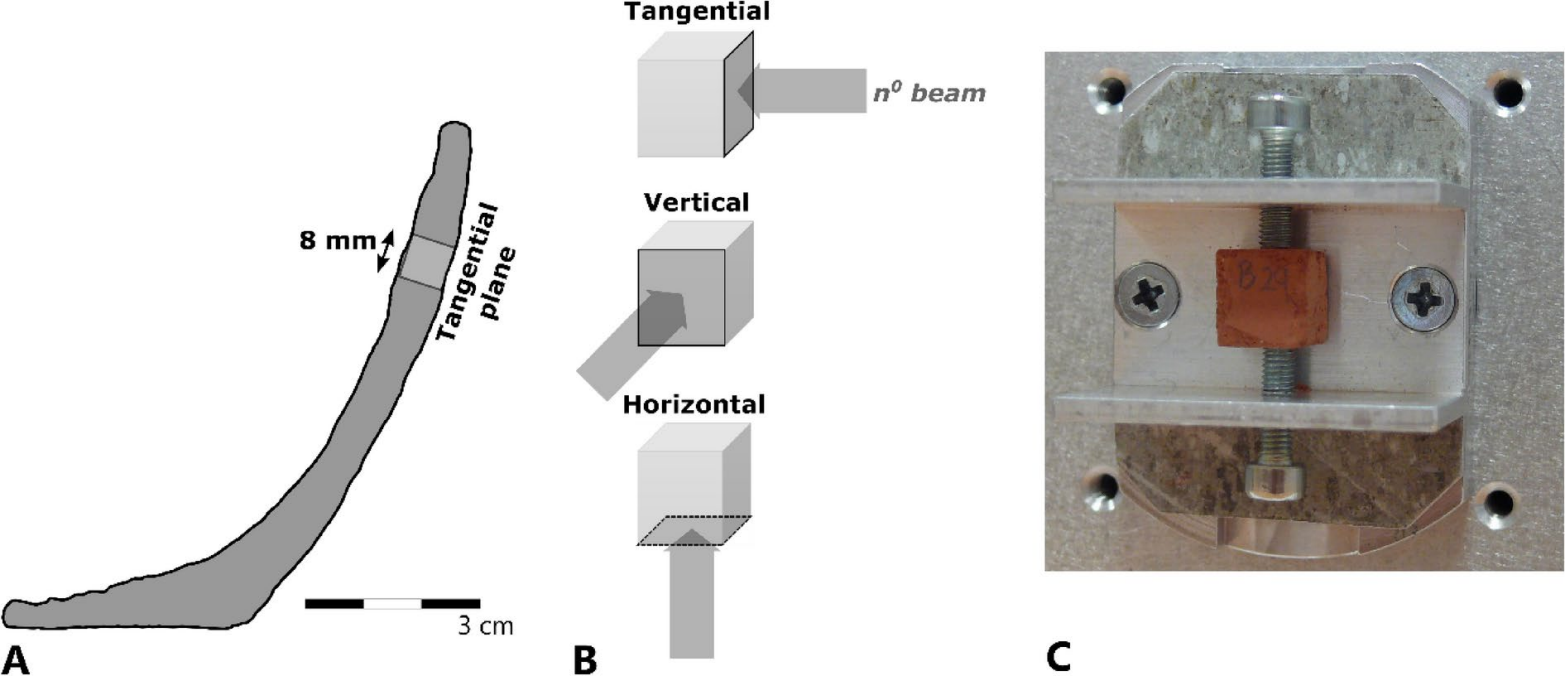
Measurement of cube samples cut from vessel-quarters. **A** Vertical cross-section of a vessel showing the location and orientation of a cube sample, with the exterior wall of the vessel defining the tangential plane of the cube; **B** Relative locations of tangential, vertical, and horizontal planes of a cube sample; **C** Cube sample mounted in SANS sample holder for measurement in the tangential plane.

The first stage of measurements aimed to investigate the average 3D orientation of the scattering domains within Fabrics A and B. As each SANS measurement provides only 2D information, in order to be able to infer the orientation in 3D, each sample was measured in three perpendicular axial directions, i.e. in the tangential, vertical, and horizontal planes, defined relative to the walls and rim of each vessel (Fig. 3). Consequently, so that the length of the path travelled by the neutron beam through each axis of each sample was approximately constant, cubes of c. 8 mm × 8 mm × 8 mm were prepared from the fired samples of Fabrics A and B. The effects of firing temperatures were investigated by repeating the measurements using samples fired at 650, 800, and 950°C. This temperature range encompasses that commonly reported for archaeological pottery.

#### Intra-vessel variability

**Fig. 4:**
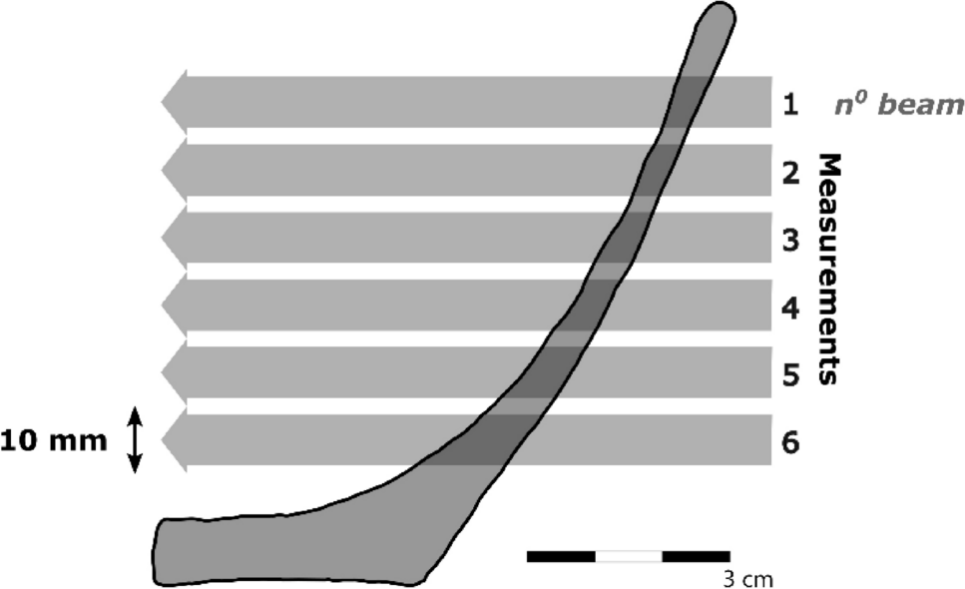
Schematic diagram of the vertical cross-section of an experimental vessel-quarter showing positions of multiple measurements along a vertical transect. The measured tilting angles were subsequently mathematically corrected to compensate for the slope of the sample relative to the beam at each measurement location.

The second stage of measurements examined potential variation within vessels according to thickness, measurement location, and beam diameter. For this purpose, single vessel-quarters of each forming technique of Fabrics A and B were measured at regularly spaced intervals along a vertical transect, starting from c. 15 mm below the rim and continuing towards the base, but stopping before reaching the thickest part of the base (Fig. 4). The sequences of measurements were repeated using 4, 10, and 16 mm diameter beams. In addition, in order to determine whether the boundaries between coils might be detected in coil-based (i.e. coil-built and coil-wheeled) vessels specifically, the coil-built samples were also measured using a 2 mm diameter beam. Unlike the measurements of the cube samples, values were determined for the tangential plane only and, therefore, no additional samples needed to be cut from the vessel-quarters.

#### Comparisons with tomography

The third stage of measurements aimed to compare the orientation of nanoscale scattering domains, determined by SANS, with equivalent measurements of the orientation of microscale objects obtained from tomographic imaging. For this purpose, it was necessary to use sherds made from a coarse-textured fabric, in order for there to be particles and voids large enough to be detected by tomographic imaging. Accordingly, sherds and coil sections of Fabric C, previously examined by X-ray microtomography (µ-CT) and neutron tomography (NT)^18^ were reanalysed by SANS, using an 8 or 16 mm diameter beam. This also enabled investigation of the nanostructure of percussion-built vessels. SANS measurements of the Fabric C samples were made in the tangential plane only, without any additional destructive sub-sampling.

## Results and discussion of experimental samples

The results from the SANS measurements of the experimental samples (Supplementary Material B Tables S1-6) are presented in terms of the tilting angle and isotropy of the interfaces of the scattering domains. The tilting angles are treated as axial circular data, using the appropriate circular statistics to calculate means and standard deviation^66^, and for convenience are reported here in the 0° to ± 90° range, without the equivalent symmetrical axial values in the ± 90° to ± 180° range (unless noted). As isotropy is a linear ratio, mean values and standard deviations are calculated with conventional linear statistics.

### Effects of firing temperature

**Fig. 5:**
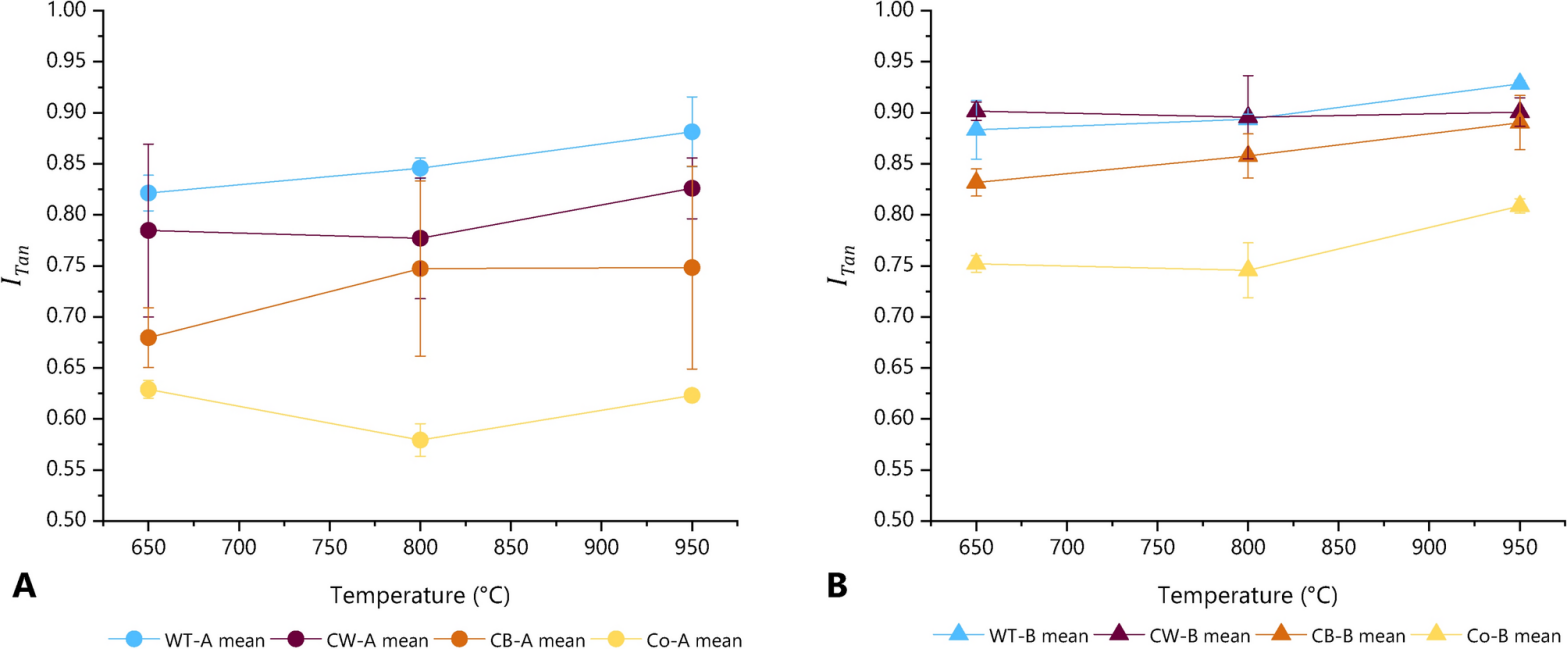
Isotropy (measured in tangential plane) plotted against firing temperature for cube samples of Fabrics A (left) and B (spread =  ± 1 SD).

Among the measurements of the cube samples for each forming technique and in each plane, no significant correlation is seen between firing temperature and tilting angle (Supplementary Material A Figure S7). However, mean isotropy tends to show a slight overall increase with increasing temperature (Fig. 5), although in some instances slight decreases are also seen. This increase in isotropy may be due to a decrease in the alignment of clay particles within the fabric as they undergo increased sintering at higher temperatures (eventually resulting in a vitrified amorphous structure), the loss of interfaces as scattering domains fuse, or the evolution of spherical gaseous pores as a result of the thermal breakdown of clay minerals^14^. These effects may also result in an increase in the smoothness of the interfaces^49^. Nevertheless, while such effects may be expected to adversely affect isotropy and tilting angle as the degree of vitrification becomes significantly pronounced above c. 950 °C, they do not appear to have a significant detrimental effect within the temperature range investigated within the present study. Therefore, in the following evaluations and discussions, the values measured for all three of the firing temperatures investigated are combined.

### 3D orientation

The tilting angle and isotropy measured in multiple planes of the cube samples may be used to investigate the effects of different forming techniques on the overall behaviour of the scattering domains in 3D space.

#### Tilting angles

**Fig. 6:**
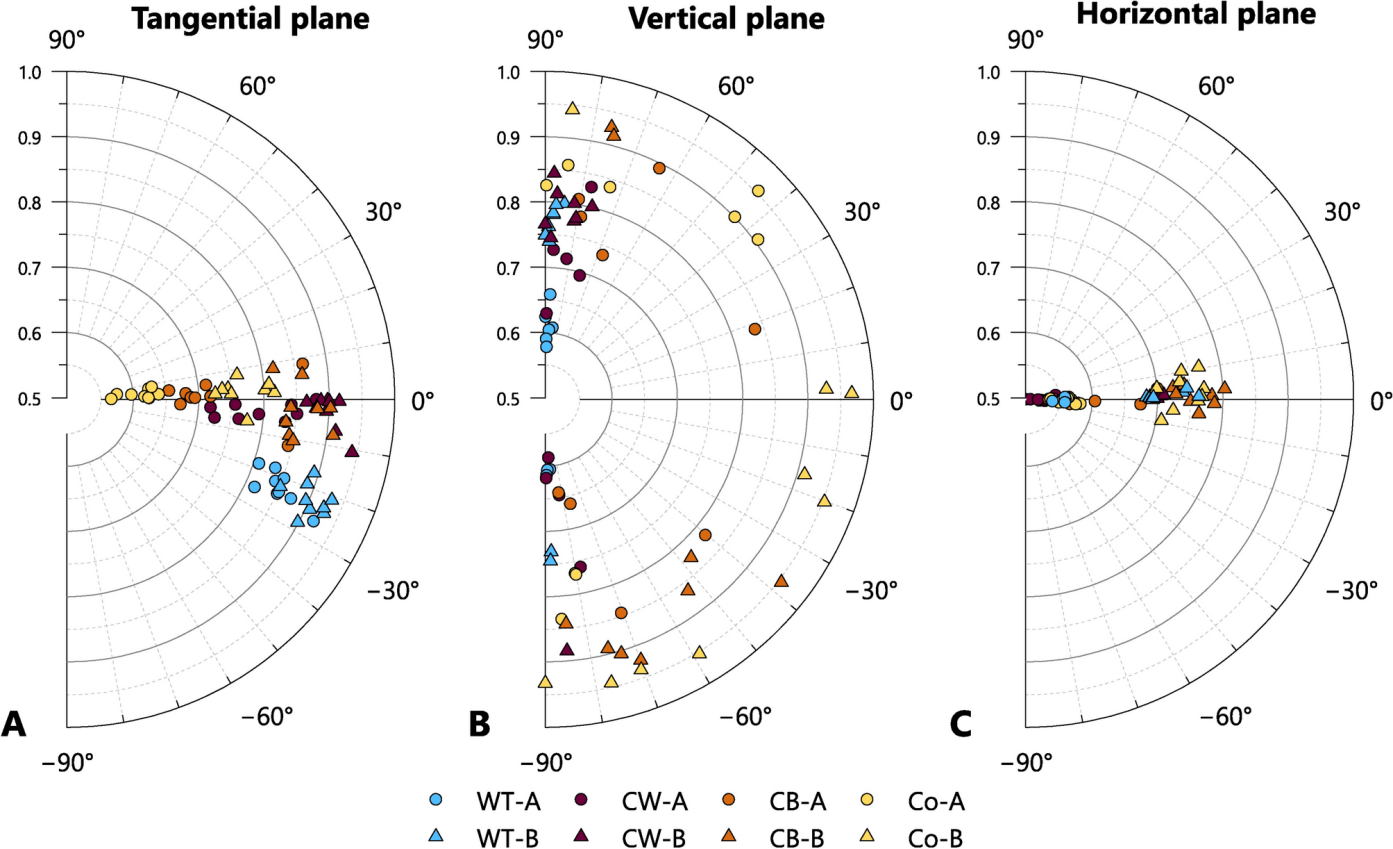
Tilting angle (angular axis) against isotropy (radial axis) for cube samples.

The tilting angles measured in each plane of 72 cube samples are shown in Fig. 6 and summarised in Supplementary Material B Table S2. In the tangential plane, for both Fabric A and Fabric B, the coil-based samples (i.e. CW, CB, and Co), all display low mean tilting angles (${\overline{\alpha }}_{Tan (A\&B)}$ = 0.3°, circular standard deviation (CSD) = 5.1°, number of samples (n) = 54). By contrast, the wheel-thrown samples all display steeper mean tilting angles (${\overline{\alpha }}_{Tan (A\&B)}$ = – 22.3°, CSD = 2.9°, n = 18), and all with a negative direction (i.e. within the -90° to 0° quadrant, Fig. 6A). This consistency in both the magnitude and direction of ${\alpha }_{Tan}$ among the wheel-thrown vessels probably derives from both the overall similarities in the size and shapes of the experimental vessels, and the specific technological habits of the potter, who used a wheel turning in an anticlockwise direction; had the vessels been made with the wheel turning in a clockwise rotation, it is anticipated that ${\alpha }_{Tan}$ would have been of similar magnitude but with a positive direction (i.e. 0° to 90° quadrant). Furthermore, the fact that such similar results were seen in both Fabric A and Fabric B suggests that the differences in their composition had little effect on the orientation of the scattering domains.

In the vertical plane, the wheel-thrown and coil-wheeled samples of both fabrics display mean tilting angles close to 90°, with low standard deviations for the former and slightly higher standard deviations for the latter (WT ${\overline{\alpha }}_{Ver (A\&B)}$ = 88.9°, CSD = 2.5°, n = 18; CW ${\overline{\alpha }}_{Ver (A\&B)}$ = 86.6°, CSD = 7.4°, n = 18). For the coil-built and coil-section samples, the tilting angle varies in magnitude more widely between samples (Fig. 6B), with correspondingly high standard deviations within each group (CB ${\overline{\alpha }}_{Ver (A\&B)}$ = –80.8°, CSD = 29.1°, n = 18; Co ${\overline{\alpha }}_{Ver (A\&B)}$ = 89.7, CSD = 42.9°, n = 18). Unlike for the preceding measurements, therefore, the specific values of the tilting angles for the coil-built and coil-section samples in the vertical plane should not be accorded particular significance; rather instead, the high variability between measurements, and resulting high CSD, appears to be the characteristic feature.

In the horizontal plane (Fig. 6C), all samples consistently display mean tilting angles close to 0° and low standard deviations. Accordingly, measurements of the tiling angle in this plane do not appear to be useful for the differentiation of forming techniques.

#### Isotropy

**Fig. 7:**
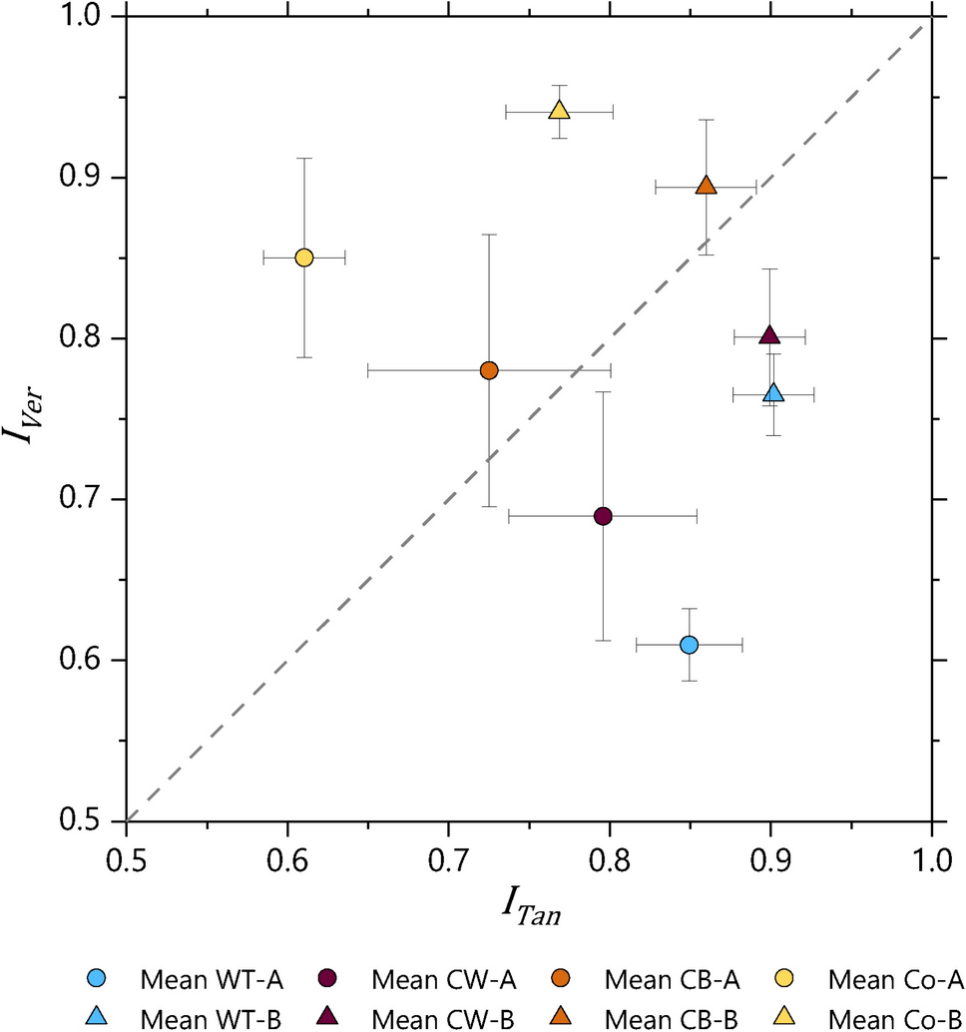
Mean isotropy in tangential plane against mean isotropy in vertical plane, showing forming techniques involving the use of a wheel (i.e. wheel-throwing and coil-wheeling) plotting below the line ${I}_{Tan}={I}_{Ver}$, and coil-built and coil-section samples above (spread =  ± 1 SD).

Absolute differences in mean isotropy in each measurement plane of the cube samples can be observed between the fabrics, with Fabric B consistently showing higher values than the corresponding measurements in Fabric A (Figs. 6, 7, and Supplementary Material B Table S3). However, similar patterns of relative variation in isotropy with regard to forming technique are seen within both fabrics, suggesting that isotropy is determined by both the composition of the samples as well as by forming techniques. In both Fabrics A and B, measurements in the tangential plane show the highest mean isotropy values for wheel-thrown samples, followed by coil-wheeled, coil-built and coil-section samples. For measurements in the vertical plane, the sequence is reversed, with coil-section samples showing the highest mean isotropy. Therefore, it is possible to differentiate wheel-made vessels (i.e. wheel-thrown or coil-wheeled vessels) from coil-built vessels by the relative magnitude of the mean isotropy in the vertical and tangential planes; for wheel-made vessels ${\overline{I} }_{Tan}$ > ${\overline{I} }_{Ver}$, while for coil-built vessels ${\overline{I} }_{Tan}$ < ${\overline{I} }_{Ver}$ (Fig. 7). In the horizontal plane, for both fabrics, the largest isotropy is seen with the coil-built samples, followed by coil-section, wheel-thrown and coil-wheeled samples respectively. For both fabrics, the mean isotropy values in the horizontal plane are lower than the corresponding values in the tangential or vertical planes, and also show less variation between forming techniques.

#### Scattering domains in 3D

**Fig. 8:**
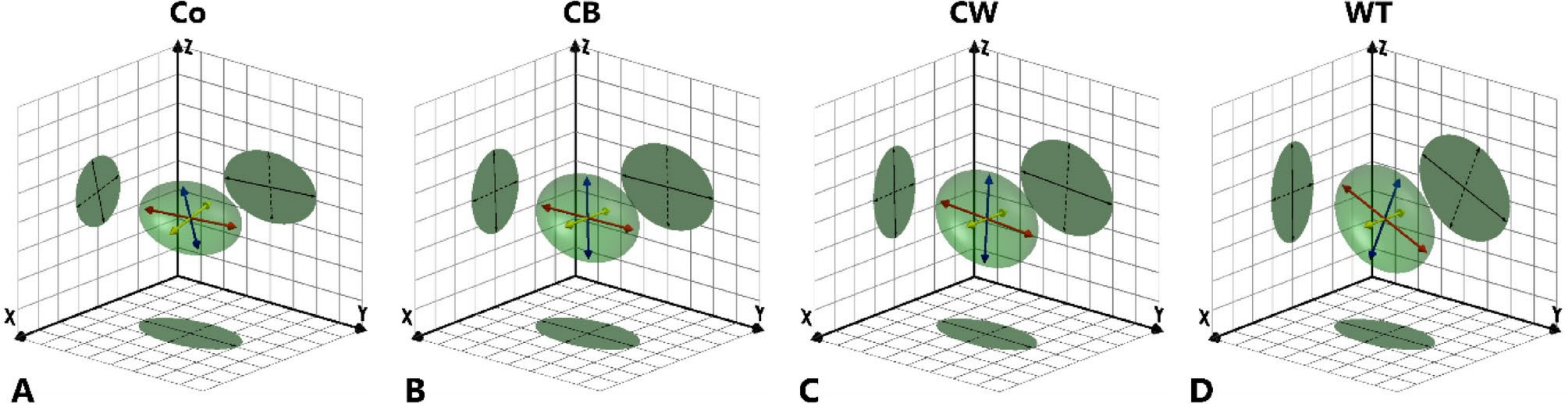
Average orientation and relative elongation of scattering domains in 3D space for samples of Fabric A, determined from mean tilting angle and isotropy values measured in tangential, vertical, and horizontal planes. **A** Coil-section; **B** coil-built; **C** coil-wheeled; **D** wheel-thrown. Primary, secondary and tertiary axes of the ellipsoids shown as red, blue and yellow arrows respectively. The position of the major axis of the 2D ellipse in each plane corresponds to the mean tilting angle in that plane, while the aspect ratio of the ellipse (width/length) equals the mean isotropy. The intersection of the projections of the ellipses in each plane combine to form a tri-axial ellipsoid, representing the mean 3D orientation and elongation of the scattering domains. (See Supplementary Material A Figures S8-S11 for interactive 3D models). XY = horizontal plane (i.e. parallel to vessel rim-plane); XZ = vertical plane (i.e. perpendicular to walls and rim-plane); YZ = tangential plane (i.e. parallel to walls).

The results from each 2D plane may be considered together to infer information about the average 3D characteristics of the scattering domains within the cube samples. Accordingly, the tilting angle and isotropy measured in each plane may be viewed respectively as the orientation and relative dimensions of an ellipse projected onto that plane by a tri-axial ellipsoid (Fig. 8), with the shape of the ellipsoid itself determined by both the average orientation and elongation of the scattering domains within the sample.

From this perspective, the consistently higher isotropy values measured for Fabric B (Supplementary Material B Table S3) indicate that the scattering domains themselves were, on average, less elongated in shape than those of Fabric A. However, the similarities in the tilting angles and relative order of the isotropy values, indicate that the scattering domains in Fabric A and Fabric B responded in similar manners to each forming technique.

For the samples from the coil-sections, the ellipsoid shows substantial elongation in the horizontal and tangential planes (Fig. 8A; indicated by low ${\overline{I}}_{Hor}$ and low ${\overline{I}}_{Tan}$) aligned to the long axis of the coil (i.e. horizontally in the tangential and horizontal planes; low ${\overline{\alpha }}_{Hor}$ and low ${\overline{\alpha }}_{Tan}$), whereas in the vertical plane, the ellipsoid is less elongated (indicated by high ${\overline{I}}_{Ver}$, especially for Fabric B). Together these indicate that, on average, the scattering domains themselves are also elongated in at least one axis, with that axis aligned to the axis of the coil. However, from these measurements alone, it cannot be determined whether the comparative lack of elongation of the ellipsoid in the vertical plane results from the individual scattering domains themselves having a similar relative width and thickness (i.e. an elongated prism, or a rod-like shape in 3D), or from their lack of preferential alignment around the axis of the coil.

For the coil-built samples, the ellipsoid shows a similar orientation and shape to that of the coil-section samples (Fig. 8B), but with slightly less elongation in the tangential plane (indicated by higher ${\overline{I}}_{Tan}$) and more elongation in the vertical plane (lower ${\overline{I}}_{Ver}$). This suggests that the original orientation of the scattering domains attained during the preparation of the coils is largely retained after they have been joined together during the coil-building process. Nonetheless, the slight increase in elongation in the vertical plane, compared to that of the coil-section samples, suggests that on average the scattering domains are also aligned slightly more closely to the walls of the vessel. For the ellipsoid to align horizontally in the tangential and horizontal planes, whilst also showing a degree of vertical alignment in the vertical plane, indicates that on average the scattering domains are not, in fact, equidimensional in their transverse cross-section, but instead have a more elongated platy 3D shape rather than rod-like shape.

For the coil-wheeled samples, the ellipsoid also shows a similar orientation to that of the coil-built and coil-section samples (Fig. 8C; low ${\overline{\alpha }}_{Tan}$ and ${\overline{\alpha }}_{Hor}$), but with less elongation in the tangential plane (higher ${\overline{I}}_{Tan})$ and increased elongation in both the horizontal and vertical planes (lower ${\overline{I}}_{Hor}$ and ${\overline{I}}_{Ver}$). A further difference between the coil-wheeled samples and the coil-built and coil-section samples is seen in the significant reduction in the CSD of the tilting angle in the vertical plane. Considered together, these data suggest that while on average the scattering domains remain aligned parallel to the rim, they are also increasingly aligned to the walls of the vessel; thereby providing further confirmation of the plate-like character of the scattering domains.

For the wheel-thrown samples, the ellipsoid shows a steeper angle of inclination relative to the horizontal plane (i.e. the rim-plane of the vessel; Fig. 8D; largest magnitude ${\overline{\alpha }}_{Tan}$ of all the forming techniques examined here) compared to that of the coil-wheeled samples, and a further reduction in elongation in the tangential plane (highest ${\overline{I}}_{Tan}$). In the vertical plane there is again a further increase in elongation (lowest ${\overline{I}}_{Ver}$ and lowest circular standard deviation ${\alpha }_{Ver}$), and an increase in mean tilting angle (highest ${\overline{\alpha }}_{Ver}$). However, contrary to preceding trends, the projection of the ellipsoid of the wheel-thrown samples is less elongated in the horizontal plane than that of the coil-wheeled samples (i.e. ${\overline{I}}_{Hor (WT)}$>${\overline{I}}_{Hor (CW)}$). This reduction in elongation in the horizontal plane appears to be caused by the larger magnitude of the tilting angle of the wheel-thrown ellipsoid from the horizontal plane, which also results in a corresponding increase in elongation in the vertical plane. From this it may be inferred that the scattering domains in the wheel-thrown samples are, on average, aligned closely to the walls of the vessel, whilst also being tilted relative to the rim. This evidence from the wheel-thrown samples also further confirms the average elongated and platy shape of the scattering domains in all of the samples, which appears to accord well with the layered structure of clay minerals used in the manufacture of pottery (e.g. smectite, illite, kaolinite^3,67^).

### Intra-vessel variability

The results from the cube samples demonstrated that the most substantial differences in the structure of samples made using wheel-throwing and coil-based forming techniques were seen in the tilting angle measured in the tangential plane. This finding agrees with those of previous microstructural studies that have used the orientation particles and voids, measured in tangential planes or slices, to identify wheel-throwing ^20,68,69^. Therefore, in most instances, the investigation of ancient forming techniques using SANS may be simplified by measuring in the tangential plane alone, thereby also avoiding the need for invasive sampling and additional damage to archaeological pottery sherds. However, for the practical application of SANS, it is also necessary to consider the extent to which tiling angle may vary within individual vessels.

#### Sample thickness

Sample thickness is, in general, an important consideration in SANS measurements^70^, owing to the increased possibility of multiple internal neutron scattering with increasing thickness. For pottery, cross-sectional thickness may vary significantly between separate vessels, or along the profile of individual samples. Nevertheless, Botti and co-workers concluded that for ceramics, at a high scattering vector range, no correction for multiple scattering was necessary^52^. A similar conclusion was also reached in an earlier investigation by members of the present study, which found that sample thickness had no observable effect on the fractal dimension of scattering surfaces for ceramic samples less than 8 mm thick^49^. However, the potential effects of thicker samples were not investigated, nor were the effects on tilting angle or isotropy considered.

In the present study, the potential effect of sample thickness was examined when undertaking multiple measurements along the vertical transect of vessel-quarters. In this context, sample ‘thickness’ represented the straight-line length of the path taken by the neutron beam through the sample at each measurement location, and therefore depended on the slope of the vessel wall relative to the beam, as well as the more conventional thickness measured perpendicular to the walls (Fig. 4).

From the vertical transect measurements (Supplementary Material B Table S4), no significant correlations were found between sample thickness and either ${\alpha }_{Tan}$ or ${I}_{Tan}$ for measurements made in the high scattering vector range, where thickness at the point of measurement was less than 25 mm. Only two measurement locations exceeded 25 mm in thickness, corresponding to measurements made near the base of sample B31 (using 10 mm and 16 mm Ø beams respectively). For both measurements the corresponding values of ${\alpha }_{Tan}$ exceeded values recorded within the same sample at other locations with thicknesses of less of than 25 mm, therefore, out of precaution, these measurements were excluded from further consideration.

#### Variation in tilting angle

**Fig. 9:**
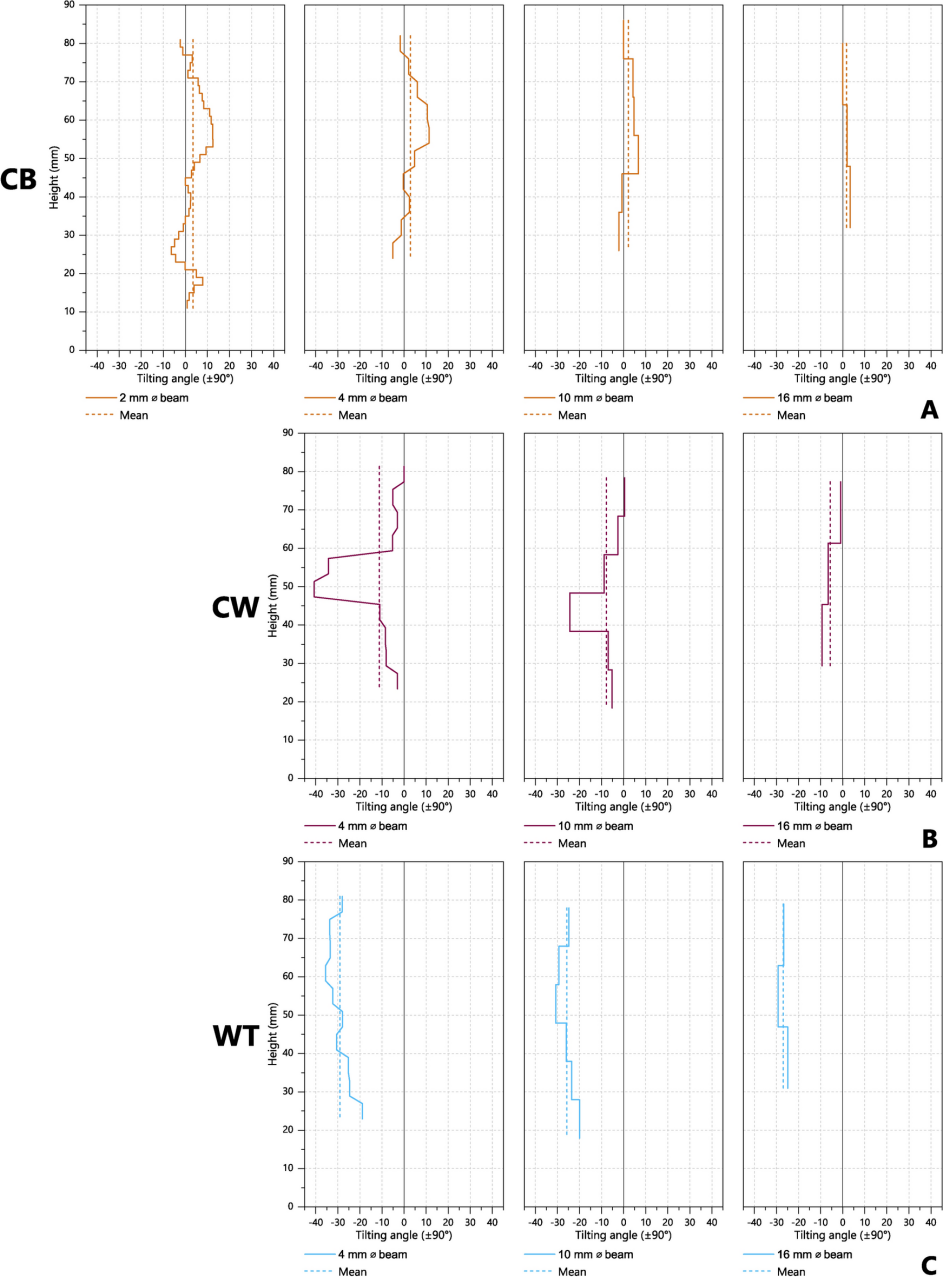
Tilting angle, ${\alpha }_{Tan}$, measured at multiple locations across vertical transects of vessel-quarters of Fabric A, using various beam diameters. **A** Coil-built sample A31; **B** coil-wheeled sample A19; **C** wheel-thrown sample A8 (for Fabric B see Supplementary Material A Figure S12).

Potential variation in the value of ${\alpha }_{Tan}$ at different locations within individual vessels was examined by taking multiple measurements along vertical transects of wheel-thrown, coil-wheeled, and coil-built vessel-quarters of Fabrics A and B. The results from the vertical transect measurements for Fabric A are shown in Fig. 9 (for Fabric B, see Supplementary Material A Figure S12; see also Supplementary Material B Tables S4 and S5), where it can be seen that the coil-built and coil-wheeled samples tend to show greater variation in ${\alpha }_{Tan}$ across the transect than do the wheel-thrown samples, especially for narrower beam diameters. This variation within the coil-wheeled and coil-built samples may result from their component coil sections, and reflect variations in the orientation of the scattering domains within different parts of the coils, or the uneven movement of clay during the joining of the coils. With regard to the former, there may be some variation between the centre and edges of coils, with clay particles and voids near the surface becoming aligned to the long axis and circumference of the coil, but those in the core retaining a more disorganised structure^71^. The extent of such potential variation within coils requires further investigation, although the results from the coil-section cube samples nevertheless indicate that the net orientation of the primary axis of the scattering domains is close to the long axis of the coils. The movement of clay during forming, to join successive layers of coils, may also have had an uneven effect on ${\alpha }_{Tan}$. In the construction of the coil-wheeled and coil-built vessels, clay was moved in an approximately vertical direction from the exposed outer edges of the coils and redeposited between the coil layers, but with a realigned orientation (Supplementary Material A Figure S2). The overall effects of such movements of clay on ${\alpha }_{Tan}$ may have varied across a vessel according to the quantity of clay moved and the exact direction of movement. Nonetheless, some indication of the initial layered structure of the coil-based vessels may be seen in the closely spaced measurements made using a 2 mm beam (Fig. 9A), where a periodicity in increasing and decreasing values of ${\alpha }_{Tan}$ may be seen, spaced at intervals approximately corresponding to the height of the coil layers (c. 14 mm).

In addition to the potential effects on ${\alpha }_{Tan}$ resulting from the joining of horizontal layers of coils, some localised variation in ${\alpha }_{Tan}$ may have been caused by the vertical joining of the ends of coils within a layer. Such a feature may account for the unusually high magnitude of the tilting angle in approximately the middle of coil-wheeled sample A19 when measured using the 4 and 10 mm Ø beams (max. $\left|{\alpha }_{Tan}\right|$ = 40.7° and 24.4° respectively, Fig. 9B; Supplementary Material B Table S4). However, such an interpretation is difficult to confirm with certainty as, when examined further by X-ray radiography (Supplementary Material A Figure S13), no clearly discernible anomalies can be seen. Nonetheless, it may be noted that horizontal coil joins also cannot be seen, illustrating the potential difficulty of identifying coil-building with radiography.

In contrast to the coil-built and coil-wheeled samples, the wheel-thrown samples tend to show more stable ${\alpha }_{Tan}$ values, indicating more homogeneous nanostructures. Nonetheless, some variation in ${\alpha }_{Tan}$ in the wheel-thrown vessel-quarters can be seen, especially with narrow beam diameters. For wheel-thrown sample A8 (Fig. 9C), when measured with a 4 mm diameter beam, the magnitude of the tilting angle gradually increases from 18.9° at 25 mm from the base, to a maximum of 35.5° at 61 mm, before decreasing towards the rim of the vessel (comparable values are also seen with Fabric B, sample B8: min. $\left|{\alpha }_{Tan}\right|$ = 17.8° at 37 mm, max. $\left|{\alpha }_{Tan}\right|$ = 25° at 73 mm, 4 mm Ø beam; Supplementary Material B Table S4).

**Fig. 10:**
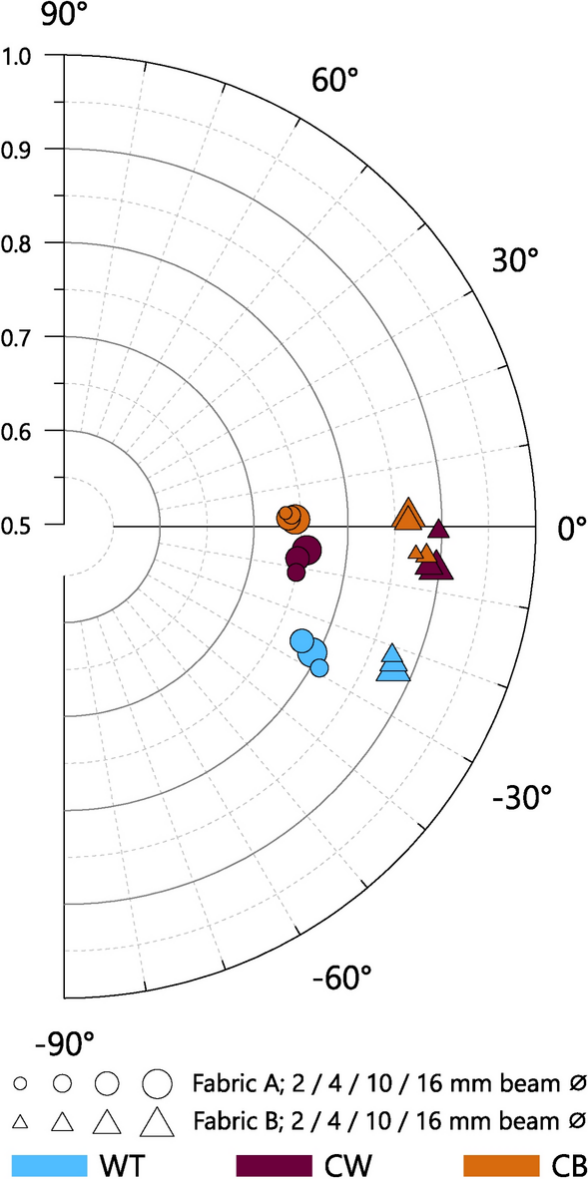
Mean tilting angle (angular axis) against mean isotropy (radial axis), for multiple measurements made in the tangential plane along vertical transects of wheel-thrown, coil-wheeled, and coil-built vessel-quarters of Fabrics A and B, repeated using various beam diameters.

With regard to identifying forming techniques, such potential variation in ${\alpha }_{Tan}$ within individual samples may cause some difficulties where the range of values from different forming techniques overlap, as in the case of the steep tilting angles seen for some measurements of the Fabric A coil-wheeled and wheel-thrown samples (Fig. 9B-C). However, in practice, wheel-thrown samples can be differentiated more consistently from coil-built and coil-wheeled samples when comparing mean tilting angles from multiple measurements (Fig. 9 and Fig. 10).

#### Variations with beam diameter

The effects of beam diameter on the values of ${\alpha }_{Tan}$ were also investigated, in order to compare highly localised measurements (made with a narrow beam) against measurements in which the orientations of scattering domains were averaged over a wider area (wide beam). While multiple measurements made along a vertical transect of a vessel may be used to investigate subtle, localised features, the overall task of differentiating wheel-throwing from coil-based forming techniques may be more efficiently achieved with the use of larger diameter beams. As seen in Fig. 9, increasing the diameter of the beam used for each measurement across the vertical transect reduces the overall variation in ${\alpha }_{Tan}$ within each vessel-quarter and removes extreme values seen with smaller diameters beams, but does not significantly affect isotropy (Fig. 10). The use of large beam diameters also substantially increases the volume of sample measured at each location, and therefore reflects the average orientation of a larger number of scattering domains (Supplementary Material B Table S5). Consequently, a larger beam diameter provides a more reliable method for differentiating forming techniques, especially for archaeological pottery sherds that may be too small to easily permit multiple measurements to be made. The use of a large diameter beam also has the additional benefits of significantly reducing both the number of individual measurements needed per sample, and also the time needed for each measurement, as a larger proportion of the incident neutron beam may be utilised.

### Comparisons of SANS and tomography

The use of SANS to differentiate pottery forming techniques may be compared with that of µ-CT and NT, with the additional aim of determining whether nanoscale scattering domains behave in a similar manner to microscale segmented objects in response to the forces applied during forming.

In an earlier study^18^, µ-CT and NT were used to differentiate coil-built vessels from those made with the tamper-and-concave-anvil percussion-building forming technique (PB), using the orientation of segmented elongated inclusions and voids in the micron to millimetre size range (in the present study these samples are designated as Fabric C). Separate coil-sections of Fabric C were also analysed using the same methods. The 3D orientations of the inclusions and voids were described by reference their polar and azimuth angles within a spherical coordinate system. In the coil-section samples, the objects tended to lie with their primary axis aligned close to the long axis of the coils. In the coil-built vessel samples, a similar alignment was seen, with the primary axis of the objects tending to align with both the walls and the rim-plane of the vessels, whereas in the percussion-built samples the primary axis of the objects also showed strong alignment to the walls, but only slight alignment relative to the rim-plane.

The values of ${\alpha }_{Tan}$ and ${I}_{Tan}$ from SANS reflect the average orientation and relative elongation of the scattering domains within the 2D tangential plane. Therefore, for purposes of comparison, the 3D polar and azimuth angles previously determined by µ-CT and NT were used to calculate the equivalent mean tilting angle and circular standard deviation of the 2D tangential projections of the microstructural objects (${\overline{\alpha }}_{Tan (CT)}$ and ${\overline{\alpha }}_{Tan (NT)}$). The results from the SANS measurements of the Fabric C samples (Supplementary Material B Table S6), together with the corresponding µ-CT and NT values, are shown in Table 2.

**Table 2:** Mean tilting angles and distributions of scattering domains and segmented objects in samples of Fabric C, in tangential plane measured by SANS, µ-CT and NT. For µ-CT and NT, objects with volume > 0.01 mm^3^ and aspect ratio < 0.3 were used.

**Sample**	**Forming technique**	${\overline{\boldsymbol{\alpha }}}_{{\varvec{T}}{\varvec{a}}{\varvec{n}}({\varvec{S}}{\varvec{A}}{\varvec{N}}{\varvec{S}})}$**(°)**	${\overline{{\varvec{I}}}}_{{\varvec{T}}{\varvec{a}}{\varvec{n}}({\varvec{S}}{\varvec{A}}{\varvec{N}}{\varvec{S}})}$	${\overline{\boldsymbol{\alpha }}}_{{\varvec{T}}{\varvec{a}}{\varvec{n}}({\varvec{C}}{\varvec{T}})}$**(°)**	${\mathbf{C}\mathbf{S}\mathbf{D}}_{({\varvec{C}}{\varvec{T}})}$**(°)**	${\overline{\boldsymbol{\alpha }}}_{{\varvec{T}}{\varvec{a}}{\varvec{n}}({\varvec{N}}{\varvec{T}})}$**(°)**	${\mathbf{C}\mathbf{S}\mathbf{D}}_{({\varvec{N}}{\varvec{T}})}$**(°)**
C1	PB	17.3	0.986	17.7	42.5	12.0	39.3
C2	PB	5.5	0.961	17.4	28.2	19.2	29.2
C3	PB	-3.5	0.966	-5.3	29.4	-6.8	33.4
C4	CB	1.1	0.839	-3.2	15.8	-2.2	15.1
C5	CB	-2.9	0.868	0.8	15.7	0.4	14.2
C6	CB	-3.3	0.847	0.2	15.9	0.3	16.7
C7	Co	2.0	0.821	-2.5	16.7	-1.0	16.9
C8	Co	2.0	0.813	-0.5	16.6	-0.8	17.4
C9	Co	1.2	0.809	-0.8	18.3	-1.5	17.1

**Fig. 11:**
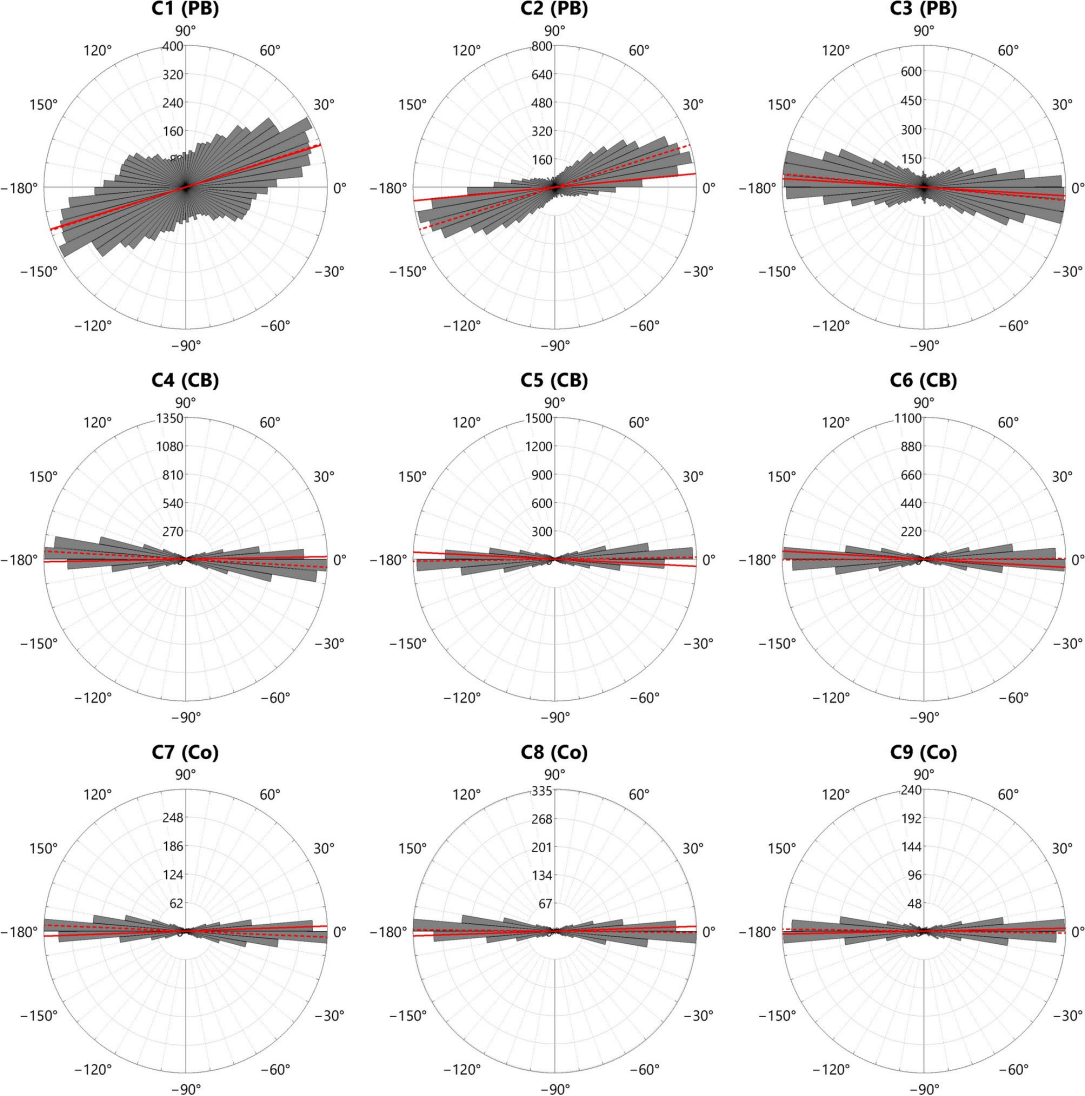
Circular frequency distribution histograms of ${\alpha }_{Tan(CT)}$ of Fabric C samples. The mean µ-CT tilting angles and the corresponding mean SANS tilting angles are shown as dashed and solid red lines, respectively.

For the coil-built and coil-section samples of Fabric C, the low mean tilting angles in the tangential plane measured by SANS agree closely with the values from µ-CT and NT. In addition, the SANS and tomography data show good agreement in the low degree of angular dispersion in the orientation of the objects in both spatial scales, as reflected by the comparatively low SANS isotropy values and corresponding low tomography CSD values. The frequency distributions of the µ-CT projected tilting angles are shown in Fig. 11, where the low values and narrow dispersions for the coil-built and coil-section samples can be seen in the tight clustering of values near the horizontal axis (for NT see Supplementary Material A Figure S14).

For the percussion-built samples, the mean tilting angles show generally good agreement between SANS, µ-CT, and NT for each individual sample, but vary more widely between the three percussion-built samples than do the values for the coil-built and coil-section samples. Such variation between the percussion-built samples appears to be due to the poor preferential alignment of objects with regard to the rim-plane, produced by the percussion-building forming technique^6,18^. More significantly, therefore, the characteristic high isotropy values of the SANS measurements, indicative of a poorly ordered nanostructure, are mirrored by the high CSD values of both the µ-CT and NT projected tilting angles (Fig. 11, Table 2). However, as shown in Fig. 11, and by the fact that the SANS mean isotropy values are less than 1, the percussion-built samples do not show an entirely random distribution in the orientation of objects in the tangential plane, but rather a slight tendency (less pronounced than with the CB and Co samples) towards low mean tilting angles. As previously reported for the µ-CT and NT analyses^18^, this slight tendency towards alignment appears to be due to the comparatively broad shape of the specific percussion-built vessels analysed (mean vessel height c. 10 cm and rim diameter c. 30 cm), which resulted from the clay being moved more in a lateral direction than vertically during the forming process.

The small differences in the mean tilting angles between SANS and tomography may be accounted for by slight discrepancies in the mounting of the samples in the SANS sample holder or in orientating the µ-CT and NT digital models relative to the 3D coordinate axes in the tomographic analysis software. However, they may also be due to localised differences in the orientation of objects within the volumes measured for each sample. The actual volume irradiated by the neutron beam at each SANS measurement location is less than 10% of the entire volume of the sample used for the µ-CT and NT measurements. Therefore, rather than representing an error between the different measurement modalities, the differences in mean tilting angle may instead reflect localised heterogeneity within the structure of percussion-built samples. In this regard, SANS offers potentially greater sensitivity as an analytical technique, as measurements may, if desired, be focused over small areas, in contrast to the comparatively larger samples that are generally required for tomographic analysis in order to record statistically significant numbers of segmented objects.

Considered together, the SANS and tomography results demonstrate that the characteristic patterns of object orientation in the tangential plane associated with coil-building and percussion-building forming techniques are seen at both the nanoscale (SANS) and microscale (µ-CT and NT). At both scales, the coil-building forming technique may be recognised by a strong tendency for objects to align horizontally when viewed in the tangential plane, as reflected by low mean tilting angle and comparatively low mean isotropy in SANS measurements, and corresponding low mean projected tilting angle and CSD in both µ-CT and NT. Similarly, at both scales of analysis, the percussion-building forming techniques may be recognised by the poor alignment of objects when viewed in the tangential plane, as displayed by high isotropy in SANS and high CSD in µ-CT and NT.

### Expected SANS ${\overline{\alpha }}_{Tan}$ and ${\overline{I}}_{Tan}$ according to forming techniques

**Fig. 12:**
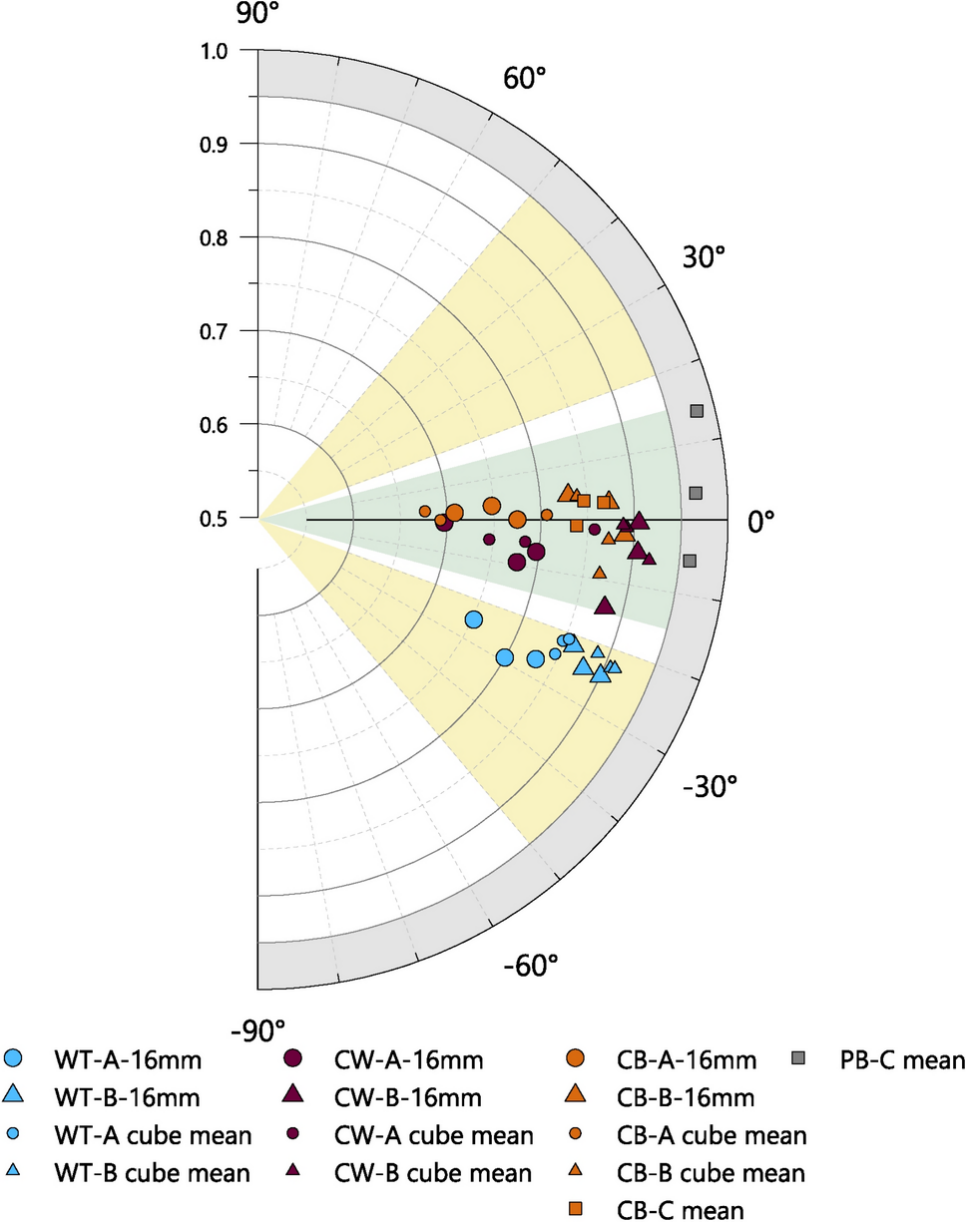
Summary of SANS tilting angle (angular axis) against isotropy (radial axis), measured in tangential plane, for experimental samples of Fabrics A, B and C, together with proposed expected ranges for different forming techniques. Yellow zones: wheel-throwing, WT, for clockwise (20° ≤  ${\alpha }_{Tan}$ ≤ 50°) and anticlockwise (– 50° ≤  ${\alpha }_{Tan}$ ≤ – 20°) wheel-rotation; Green zone: coil-building, CB, or coil-wheeling, CW, (– 15° ≤  ${\alpha }_{Tan}$ ≤ 15°); Grey zone: percussion-building, PB (0.950 ≤ ${I}_{Tan}$≤1).

The results from the experimental vessels may be used more generally to help define the expected ranges of tilting angle and isotropy values measured by SANS associated with certain forming techniques. The expected ranges are shown in Fig. 12, using values from the tangential plane only, as these can be measured without invasive sampling and therefore have greatest utility with regard to the analysis of archaeological materials.

The thresholds of these ranges are informed by either the mean values of multiple measurements, or individual measurements made with a 16 mm Ø beam, as these avoid extremes associated with small beam diameters, and give the most reliable criteria for differentiating the forming techniques of the experimental samples. Additional information from previous studies have also been considered ^6,19,22,30,32,39,72^.

Percussion-building is characterised by high isotropy; among the percussion-built samples of Fabric C, the minimum recorded mean isotropy is 0.961 (Table 2). However, as isotropy also varies to some extent according to composition, it is possible that percussion-built vessels of different fabrics could potentially also display slightly lower values. Conversely, the maximum recorded isotropy among the wheel-thrown, coil-wheeled, coil-built, and coil-section samples of Fabric A and B, as well as the coil-built and coil-section samples of Fabric C is 0.905 (sample B19, 16 mm Ø, Supplementary Material B Table S4). Accordingly, a value of 0.950 is suggested as a reasonable minimum threshold for percussion-building (Fig. 12, grey zone), with lower isotropy belonging to forming techniques that impart a more organised structure to the fabrics.

The coil-based forming techniques (i.e. coil-building and coil-wheeling) are characterised primarily by tilting angles close 0°. Among the experimental samples of Fabrics A, B and C, the maximum recorded magnitude of the tilting angle was 14.3° (sample B19, 16 mm Ø), with no consistent patterns seen among the samples regarding whether the direction was positive or negative. Accordingly, rounding up to the nearest convenient whole number, the limits of the coil-built range may be given as ± 15° (Fig. 12, green zone). As coil-building and coil-wheeling cannot be differentiated by the tangential plane tilting angle alone, both techniques are included within a single range.

Wheel-throwing is characterised primarily by tilting angles of comparatively large magnitude. The direction of tilting is determined by the direction of rotation of the wheel: clockwise wheel rotation results in a positive tilting angle, and anticlockwise rotation a negative tilting angle. Among the WT experimental samples of Fabrics A and B, the tilting angles range from -21.8° to -29.2° (samples B8 and A8 respectively, 16 mm Ø beam).

The magnitude of this range falls within the ranges previously reported elsewhere^19,30^ for wheel-thrown vessels when analysed by other methods, but does not extend as high, or as low, as suggested by some studies^32,73^. The comparatively limited range in the tilting angles reported here may be due to the limited variation in the shape and size of the experimental vessels, as well as the consistency of the throwing technique of the potter. Although the experimental vessels used here may be considered broadly comparable to many archaeologically attested forms, steeper tilting angles may potentially also be found with vessels of different shapes and sizes, or those thrown by potters using faster lifting actions or slower wheel-rotation^19^. To accommodate this additional variation, a tilting angle of 50° magnitude is proposed here as a reasonable upper limit for the expected range of tilting angles for wheel-thrown vessels, although this figure may be modified by future studies.

Tilting angles of a magnitude of less than c. 20° have been reported in the literature for wheel-thrown vessels^72,73^, however, in the present study, such low values were only seen with SANS at the extremities of the bases of wheel-thrown vessels, and when measured with a small diameter beam (Fig. 9C, 4 mm Ø beam). For some vessels, especially those with complex shapes, the bases and rims may receive additional manipulation after initial forming, and the joining of extra components (e.g. ring bases, collars, rims, and spouts, etc.) may result in localised areas where the tilting angles of particles and voids are not necessarily indicative of the initial forming technique of the vessel. Consequently, while bases and rims were used to orientate sherds for analysis, these particular areas (along with necks, handles and carination points among the archaeological samples) were intentionally avoided during SANS measurements, or excluded from subsequent evaluations. For the low tilting angles reported in the literature, the precise locations of measurements are often unclear, but in some instances may not be comparable with the SANS measurements targeting the body of vessels. Accordingly, the limits of the wheel-throwing range proposed here are ± 20° to ± 50° (Fig. 12, yellow zones), although tilting angles of lower magnitude might be expected for measurements made near the base or rim, or for exceptionally large vessels that accordingly require slow lifting speeds and slow wheel rotation.

In Fig. 12, the gaps of 5° between the limits of the expected ranges of coil-based and wheel-thrown vessels reflect areas of some uncertainly, where it is difficult to determine a specific probable forming technique based on tangential tilting angle alone, and other information may be required. Additionally, potential tilting angles of greater than 50° magnitude cannot, at present, be accounted for by the experimental samples examined in the present study. However, such very steep tilting angles may be expected for vessels made with a drawing technique^6^.

The values given here for the expected ranges of tilting angles and isotropy for various forming techniques are intended to serve only as guides rather than reflecting absolute boundaries. Wider ranges might be expected for especially large or small vessels, or vessels made with particularly fast or slow wheel rotation (which itself may be linked to vessel size, the plasticity of clay, and the choices of the potter), or vessels with unusual shapes (e.g. shallow plates, or irregular shapes). Accordingly, the results from SANS measurements are only an aid to interpretation, and determining forming techniques necessarily requires the input of additional information, such as evidence from macroscopic traces (where available), or historical evidence of techniques known to have been practiced at a particular time or place.

### Inferred 3D structures

The similarities noted here between the results of the SANS analysis and the results of previous tomographic analyses^18^, suggest that both nanoscale scattering domains and microstructural segmented objects may respond in similar manners to the forces applied during forming. Accordingly, an attempt may be made to integrate the effects observed at both scales of analysis using simplified interpretive models, based on the 3D behaviour of hypothetical particles without reference to specific spatial scales. The models proposed here draw on the results of the present study, as well as previous results and discussions within the literature, and aim to provide 3D analogues to earlier schematic 2D particle orientation diagrams^6,19,25,68,74,75^. Using this broad range of information, it is also possible to tentatively propose models for additional primary, and composite, forming techniques other than those investigated experimentally within the present study. It should be emphasised, however, that such models necessarily involve a number of assumptions and simplifications of what are complex systems; nonetheless, they may serve as a basis from which to attempt to explain some of the effects of forming techniques on the 3D structure of pottery fabrics.

**Fig. 13:**
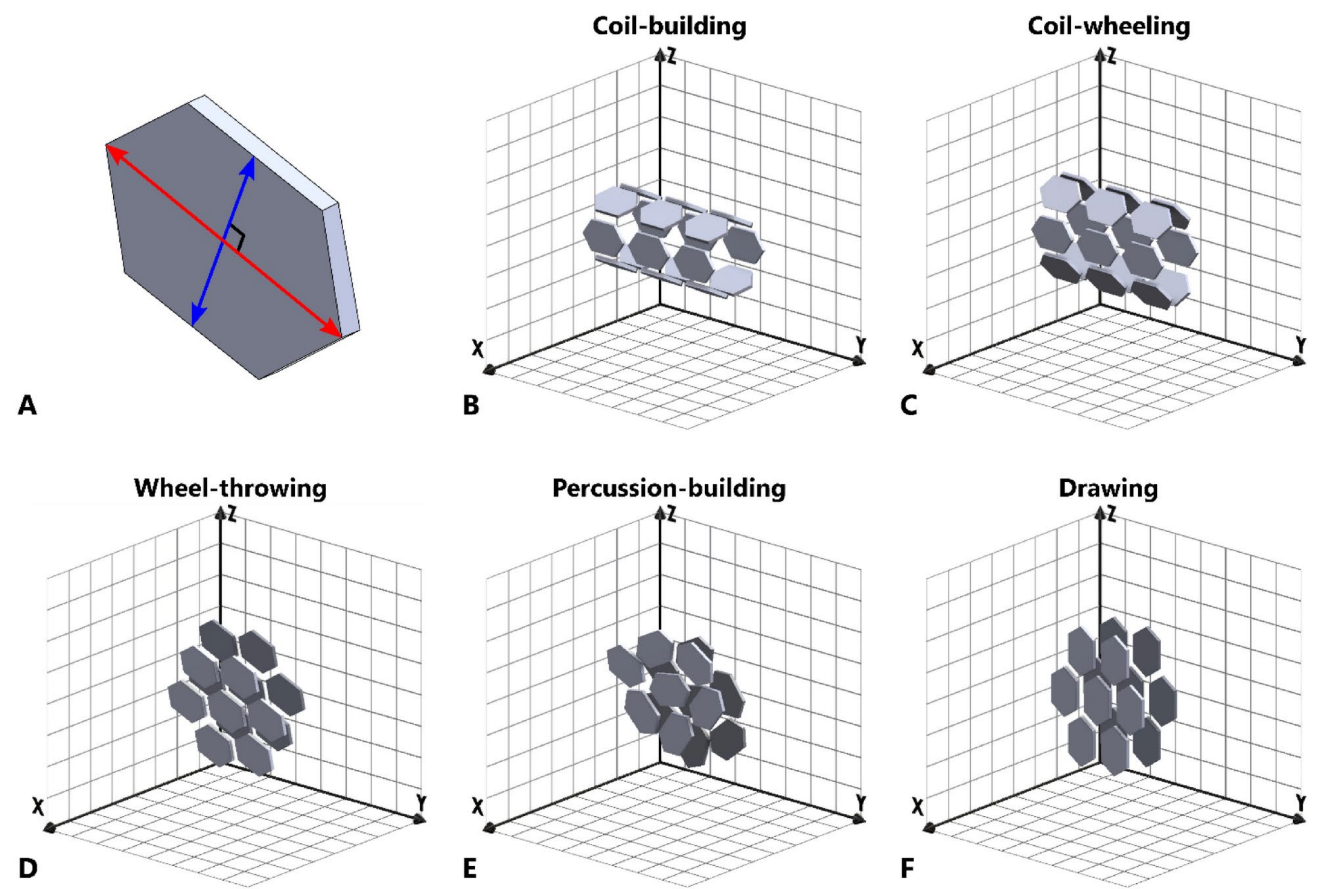
Proposed schematic models (not to scale) showing the simplified 3D orientations of hypothetical particles resulting from different forming techniques. **A** Hypothetical rigid, tri-axial particle, showing primary and secondary axes as red and blue arrows respectively; **B** coil-section/coil-building; **C** coil-wheeled; **D** wheel-thrown (anticlockwise wheel rotation); **E** percussion-building/percussion-wheeling; **F** drawing/drawing-wheeled. See Supplementary Material A Figure S15-S19 for interactive 3D models.

Within these models, both scattering domains and segmented objects are assumed to act in the manner of hypothetical particles of an identical, rigid, slightly elongated and platy appearance. Accordingly, the averaged 3D behaviours of scattering domains determined by SANS (Fig. 8), and of segmented objects determined by µ-CT and NT, may be interpreted in terms of the aggregated orientation of these hypothetical particles (Fig. 13). The 3D orientations of the particles themselves can be described by reference to the orientation their primary and secondary axes relative to the geometry of the samples (i.e. the walls and rim-plane of the vessels).

For the coil-section samples, particles become aligned towards the length and surface of the coil during the forming of the coil^71^. Accordingly, the primary axis of the particles shows strong alignment relative to the horizontal and tangential planes, while the comparatively high isotropy in the vertical plane measured by SANS (Fig. 7, Fig. 8A and Supplementary Material B Table S3) may be explained as due to the poor average alignment of the secondary axis of the particles relative to both the tangential and horizontal planes as, instead, it becomes aligned parallel to the circumference of the coil (Fig. 13B). For coil-built vessels, the alignment of the particles is largely unchanged from that of the original component coils, with the primary axis of the particles strongly aligned to both the walls and rim-plane of the vessel, and the secondary axis, on average, poorly aligned to the walls and rim-plane (Fig. 13B).

For the coil-wheeled samples, while the primary axis of the particles also continues to remain strongly aligned towards the walls and rim-plane of the sample, the secondary axis shows increased alignment towards the walls and away from the rim-plane (Fig. 13C). The increased alignment of the secondary axis towards the walls of the vessels can be most readily explained by the net lateral pressure (i.e. perpendicular to the tangential plane) applied during shaping on the wheel. For the coil-wheeled samples, this lateral pressure is also displayed by the reduction in the thickness of the walls of the vessels compared to that of the coil-built vessels.

Among the wheel-thrown samples, both the primary and secondary axes of the particles are aligned parallel to the walls of the vessel, but steeply inclined relative to the rim-plane (Fig. 13D). The steeper mean tilting angle of the primary axis relative to the rim-plane, compared to that of coil-wheeled samples, can be accounted for by the resultant movement of the clay as it is pulled in a vertical direction whilst simultaneously moving in the horizontal plane around the rotational axis of the wheel. For the wheel-thrown vessels, the clay was drawn upwards to the final height of each vessel during the forming process itself, whereas for the coil-wheeled vessels the height of the vessel was achieved during the primary coil-building stage, and the subsequent modifications on the wheel did not result in substantial additional vertical movement of the clay, but rather only some reduction in the thickness of the vessel walls. Therefore, while ${\overline{\alpha }}_{Tan}$ for the wheel-thrown samples is broadly consistent among the vessels in the present study, it might also be expected to vary according to the specific size and shape of vessels, as well as the personal choices made by the potter regarding the speed and direction of rotation of the wheel, and the speed with which the clay was drawn upwards from the wheel-head.

Within percussion-built vessels, both the primary and secondary axes of the particles show a strong tendency to align close to the walls of the vessel but with poor alignment relative to the rim-plane (Fig. 13E). When followed by secondary wheel-shaping (percussion-wheeling), on average the primary axis shows little change, but the secondary axis may become more closely aligned towards the vessel walls.

For vessels formed by drawing, both the primary and secondary axes of the particles may be expected to show a strong tendency to align close to the walls, with the primary axis also very steeply inclined relative to the rim-plane (Fig. 13F). When followed by secondary wheel-shaping (drawing-wheeling), it may again be expected that on average the primary axis will show little change, while the secondary axis may show increased alignment towards the walls.

## Archaeological case study

**Fig. 14:**
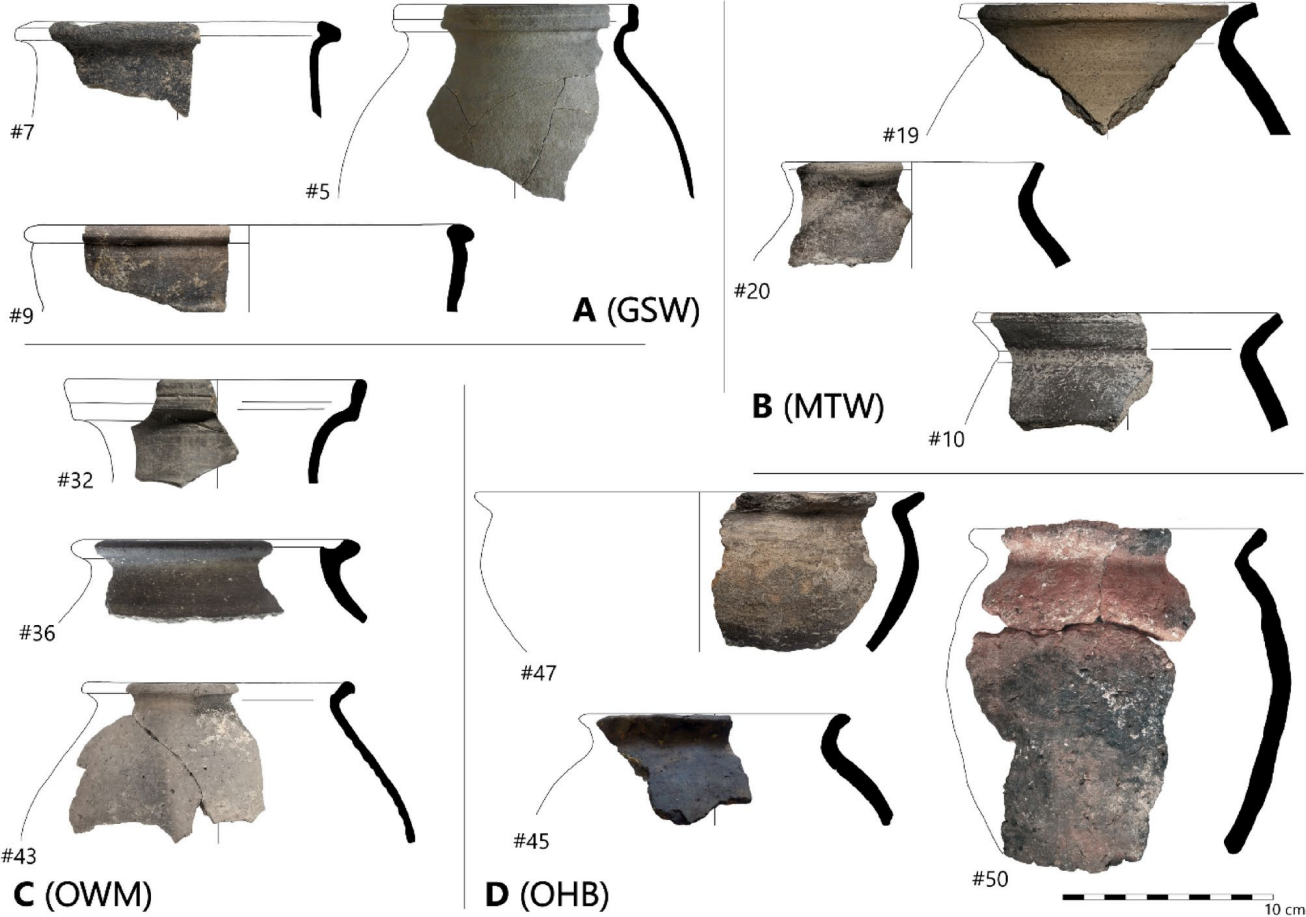
Selection of archaeological pottery sherds analysed in the present study (for all samples, see Supplementary Material A Figures S20-S26). **A** Grey Grainy Surface Ware, GSW; **B** Marble Tempered Ware, MTW; **C** Other Wheel-Made, OWM; **D** Other Hand-Built, OHB.

In order to demonstrate the application of SANS for the analysis of forming techniques, 50 sherds of ancient pottery were selected for analysis. The sherds were intentionally selected so as to span a wide range of macroscopic traces, from those with clearly discernible features derived from some sort of shaping on a wheel, through to those with more ambiguous features, and those with no evidence for any use of a wheel (Fig. 14; Supplementary Material B Table S7). Accordingly, through this diversity of macroscopic features, the sampling aimed to incorporate pottery made using a potentially wide range of hand-building and wheel-based forming techniques.

**Fig. 15:**
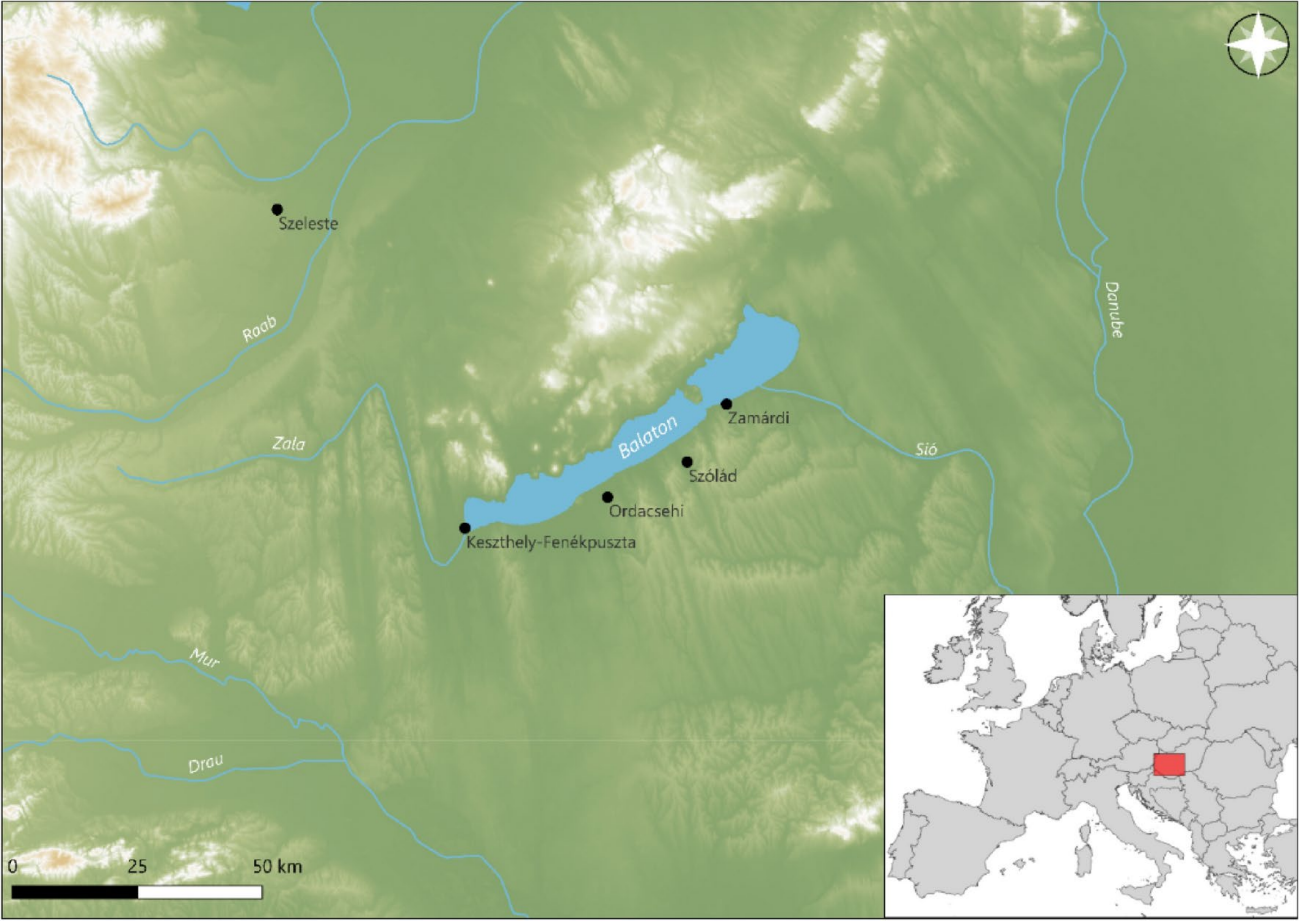
Location of sites sampled within the territory of the former Roman province of Pannonia (Hungary).

Material was selected from five sites within the territory of the former Roman province of Pannonia (Hungary, Fig. 15). The majority (n = 38) of the sherds, dated to the fourth – sixth centuries AD, were recovered from the late Roman fortress of Keszthely-Fenékpuszta^76^. Additional samples were selected from the fifth century and fifth – sixth century settlements of Ordacsehi and Zamárdi^77^, and the sixth century cemeteries of Szeleste^78,79^ and Szólád^80,81^ (see Supplementary Material A Note 4).

In general, the SANS measurement conditions for the archaeological samples matched those of the experimental samples, however, measurements were made in the tangential plane only in order to avoid the need for destructive sampling. Where possible, multiple locations on each sherd were measured using a wide beam (most often 12 to 16 mm Ø), with the results averaged. Rims and bases were used to orientate the sherds in the sample holder, but these areas, together with necks, handles and carination points were avoided as measurement locations; instead areas of the body were targeted.

### Archaeological materials

Based on their general visual appearance (i.e. colouration, surface features, fabric texture etc.), the sherds were assigned to one of four groups. The majority of the sherds represented two distinct pottery wares, while the remaining sherds, belonging to various other wares, were grouped according to whether or not they displayed macroscopic traces for the use of a wheel during forming.

Nine sherds belonged to the ‘Grey Grainy Surface Ware’ (GSW, also termed *Graukörnige Ware*^82^ and *Tokoder Keramik*^83^; Fig. 14A). This ware consists of various types of utility vessels (cooking pots, lids, bowls, plates, jugs) that were very commonly found across Pannonia in the mid-fourth and fifth centuries, and can be recognised by their distinctive grey, or bluish grey, grainy surface, probably a result of an extra slip layer mixed with coarse sand. The selected sherds, all from the fortress of Keszthely-Fenékpuszta, displayed conspicuous macroscopic traces suggestive of wheel-throwing, including prominent and often steeply inclined rilling marks on the interior, and spiral marks on the exterior of some base-sherds. Previous macroscopic analysis of a large assemblage of this ware from the same site has suggested that c. 95% of vessels were formed by wheel-throwing, with the remainder only shaped or finished on a wheel after initial forming with a hand-building technique^84^, however, the specific type of hand-building technique was not determined.

Nineteen sherds, also from Keszthely-Fenékpuszta, were assigned to another distinctive group, termed here ‘Marble Tempered Ware’ (MTW; Fig. 14B). This ware consists primarily of cooking pots and lids, and commonly appears in fifth–sixth century contexts in Pannonia, and also in the neighbouring provinces of Noricum (Austria and Slovenia) and Italia (northern Italy)^80,85–87^. Vessels of this ware are commonly dark grey/black (occasionally greyish brown) small globular-shaped pots, with flattened bases and everted rims. Stylistically they fall within the general classification of late Roman ‘grey coarse wares’ (e.g. *Graue Rauhwandige Ware*^84^), but have recently been described as a distinct ware based on the intentional technological choice of adding crushed, presumably recycled, marble as a temper^80^. Sixteen of the MTW sherds selected displayed macroscopic evidence for the use of a wheel, especially evenly shaped rims and rilling marks on the interior surfaces, although the latter often appeared less prominent than those seen on the GSW sherds. However, from macroscopic features alone, it was unclear whether these sherds had originally been thrown on a wheel, or formed using hand-building techniques with additional secondary modification on a wheel. Three MTW sherds did not display any macroscopic features derived from a wheel, and were therefore presumed to have been formed with a hand-building technique only, although whether by coil-building or another technique could not be determined from the macroscopic features preserved.

A further 16 sherds, from a range of other wares and grouped here as ‘Other Wheel-Made’ (OWM; Fig. 14C), each displayed macroscopic traces indicative of forming with the aid of a wheel. The general term ‘wheel-made’ is accordingly used as a macroscopic descriptive category for vessels made either by wheel-throwing, or by hand-building followed by secondary modification on a wheel^88^. The sherds of this group belonged stylistically to a range of common fourth – sixth century wares, and included cooking pots, jugs, deep bowls and cups.

The final category of samples, designated ‘Other Hand-Built’ (OHB; Fig. 14D), consisted of six sherds of various wares and forms (closed jars, biconical deep bowls and cooking pots), that did not show any macroscopic features indicating throwing or secondary modification with the aid of a wheel, but instead typically displayed uneven surfaces and irregularly shaped rims. Accordingly, they were identified as having been formed with one or other hand-building technique, although it was not possible to determine macroscopically the specific techniques used.

### Archaeological results and discussion

**Fig. 16:**
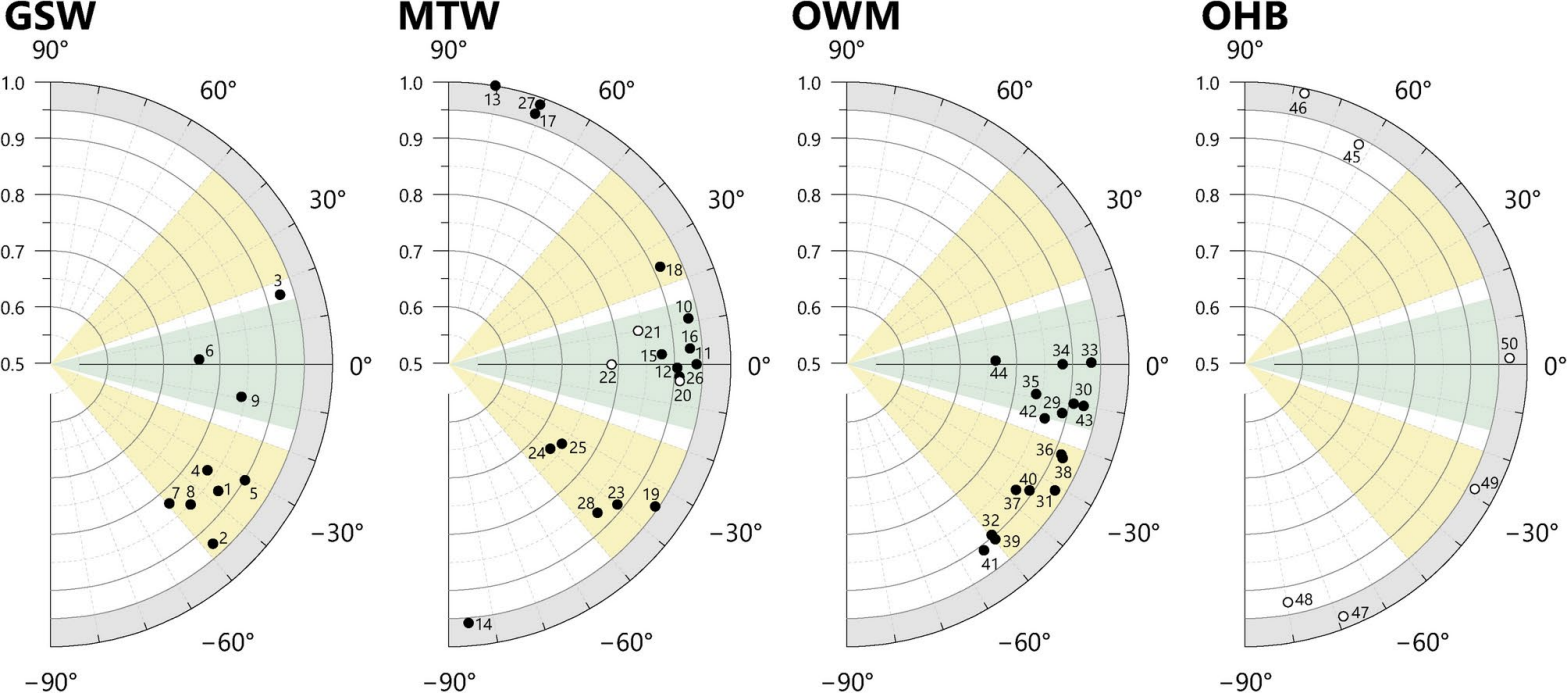
Mean SANS tilting angle (angular axis) against isotropy (radial axis), measured in the tangential plane, for 50 archaeological samples. GSW: Grey Grainy Surface Ware; MTW: Marble Tempered Ware; OWM: Other Wheel-Made; OHB: Other Hand-Built. Black dots represent samples with macroscopically visible wheel-traces, and white dots represent samples without any traces of wheel use. Yellow zones: wheel-throwing, with clockwise (positive quadrant) or anticlockwise (negative quadrant) wheel-rotation; Green zone: coil-building and coil-wheeling; Grey zone: percussion-building.

The results of the SANS measurements of the archaeological samples are shown in Fig. 16 (see also Supplementary Material B Table S7). As the coil-wheeling and coil-building techniques are both characterised by low magnitude tilting angles in the tangential plane, these two techniques were differentiated from each other by the presence of macroscopic surfaces traces indicating the use of a wheel.

Of the nine GSW samples, the majority (66.7%) fall within the range expected for wheel-throwing with an anticlockwise direction. Two sherds fall within the range expected of coil-based forming techniques, but from the presence of macroscopic traces of wheel use they may be identified more specifically as coil-wheeled. Sample 3 falls between the ranges expected for coil-wheeling and wheel-throwing with a clockwise wheel rotation, however, the pronounced internal macroscopic rilling marks and similarities with other samples suggest wheel-throwing as the more probable option. Notwithstanding the uncertainty regarding sample 3, and the small number of GSW sherds analysed, the SANS measurements broadly agree with the earlier macroscopic analysis that suggested this ware was predominantly made by wheel-throwing^84^. The predominance of wheel-throwing in GSW agrees well with our previous knowledge that this ware group, being dated broadly to the late provincial phase of Pannonia and disappearing at the end of the Roman administration (first half of the fifth century), is connected to centrally organised, industrial-scale, pottery production. However, larger assemblages of samples, and more refined chronological data, are required to further examine the sparse, but interesting, occurrence of coil-wheeled vessels among this group. These individuals may for example originate from different workshops, or reflect chronological variations in production strategies, or else specific technological choices made according to the size and shape of vessels.

The MTW sherds, by contrast, display an unexpectedly high level of diversity, with four distinct forming techniques attested: wheel-throwing (31.6%), coil-wheeling (31.6%), percussion-wheeling (21.1%), and coil-building (15.8%). Of the nine samples displaying low magnitude tilting angles, six also display macroscopic wheel-traces and therefore may be described as coil-wheeled, while the remaining sherds, lacking such wheel-traces, are identified specifically as coil-built (samples 20–22). Six sherds fall within the range for wheel-throwing, of which one appears to have been made with the wheel rotating clockwise. Four samples have isotropy values greater than 0.950, indicative of percussion-building, but also display macroscopic wheel-traces, suggesting a composite percussion-wheeling forming technique (PW), analogous to the more frequently described combination of coil-building followed by wheel-shaping (i.e. coil-wheeling). A similar technique has also recently been proposed for marble-tempered pottery from Noricum^46^. The possibility that the high isotropy values may be due to very high firing temperatures can be discounted, as all four sherds retain many marble inclusions (which undergo thermal decomposition above c. 700° C) and do not display any other features associated with high firing (e.g. bloating, a hard fabric, unusual colouration etc.). As MTW can be considered as one of the most common utility wares from the fifth and sixth centuries in Keszthely-Fenékpuszta, the observed variability of forming techniques may also reflect changes in production away from earlier, more centralised pottery centres (most likely performing wheel-throwing) to multiple smaller-scale workshops. Furthermore, as Keszthely-Fenékpuszta was an important centre for both surviving Late Antique cultural traditions and several new barbarian cultures in the early medieval period ^89,90^, it may have been home to potters from various cultural backgrounds, with skills acquired from distinct technological traditions. Such cultural diversity may therefore possibly account for some of the technological diversity of observed among the forming techniques.

For the other wares displaying macroscopic wheel-traces (OWM), the analysed sherds are evenly divided among wheel-throwing and coil-wheeling. Samples 41 and 42 show tilting angles slightly outside the guidelines previously defined for these forming techniques, but nonetheless are probably attributable to wheel-throwing and coil-wheeling respectively. Although, this group includes various wares and shapes, the balanced distribution between wheel-throwing and coil-wheeling appears to agree broadly with previous studies, where an increase in the proportion of ‘slow-wheeled’ (i.e. non-wheel-thrown, but wheel-shaped) pottery can be observed from the fourth century^91^, in contrast to the predominance of wheel-throwing seen in earlier periods. It is curious that percussion-wheeling was not observed among these samples; this technique therefore seems to be unique to MTW according to our current knowledge.

For the six hand-built sherds (OHB), four display high isotropy values, within the range expected for percussion-building. In the case of sample 49, the small size of the original vessel (height 10.5 cm, rim Ø 10.3 cm), combined with frequent macroscopic cracks and an irregular surface and rim (Supplementary Material A Figure S26), suggests that it may have been made by pinching, and as such agrees with the SANS evaluation. The two remaining sherds display tilting angles of very high magnitude, and isotropy below 0.950, which together with the absence of macroscopic wheel-traces, may be tentatively identified as having been made by drawing (samples 45 and 48). While drawing as a forming technique has not yet been systematically investigated with SANS, the steep tilting angles observed are consistent with expectations previously described in the literature^6^. With regard to isotropy, the alignment of particles by drawing would also be expected to result in an ordered structure, and comparatively low isotropy, however, further experimental studies are needed to confirm such interpretations. Nonetheless, despite these limitations, the results of the SANS measurements demonstrate how more specific forming techniques can potentially be identified among sherds otherwise characterised only as ‘hand-built’. In the present assemblage of OHB sherds, the suggestion that percussion-building and drawing may have been practiced is perhaps an unexpected finding for late Roman/early medieval pottery production in Pannonia, but, as with the MTW, may begin to hint at the existence of a more diverse range of technological practices and traditions than previously envisaged. Conversely, however, it is notable that coil-building is entirely absent from the group, despite it being, in general, the most commonly cited hand-building forming technique, and also being found among the MTW samples.

Despite the comparatively small size and intentionally varied nature of the archaeological assemblage analysed here, the interpretations of forming techniques based on the SANS measurements begin to cast new light on pottery production during the transitional late Roman/early medieval period in Pannonia. While wheel-throwing is suspected as having been practiced on a large scale in the fourth – fifth centuries, as exemplified by the abundant GSW sherds found at Keszthely-Fenékpuszta, the more surprising finding is the diversity of other forming techniques also practiced slightly later, in the fifth – sixth centuries. This is perhaps most clearly demonstrated among the 19 MTW sherds, which appear to have been made with four different forming techniques. Such diversity in forming techniques suggests that despite similarities in style and composition, and that all the MTW sherds examined in this study were recovered from Keszthely-Fenékpuszta, there were multiple distinct technological traditions in use. It also demonstrates the fundamental change in pottery technology that occurred during the end of the Roman provincial period, specifically the shift away from wheel-throwing in favour of hand-building and composite forming techniques (i.e. coil-wheeling, percussion-wheeling), providing further details to earlier suggestions of the relative increase in ‘slow wheel-made’ pottery^91^.

The identification of percussion-building and percussion-wheeling (i.e. percussion-building followed by secondary wheel modification) also represents a significant finding, as these techniques have not previously been attested for this period within Pannonia itself. In particular, the use of percussion-wheeling among Pannonian MTW vessels is of interest as it closely resembles the suggested forming techniques of stylistically similar pottery found in Noricum^46^. As such, it appears to offer further evidence for a close connection between Keszthely-Fenékpuszta and Noricum, either in terms of trade, or through the use of a shared technological tradition. Furthermore, on a more general level, while coil-wheeling is among the most common composite forming techniques described in the archaeological literature, the possibility that other hand-building techniques, including also percussion techniques and drawing, may have been combined with secondary wheel-shaping or finishing should now also be considered more closely in future studies.

The relative scarcity of coil-built vessels in the archaeological assemblage (6%), specifically without secondary wheel-shaping, is a further point of note. In this particular geographical and cultural context, the predominant use of coil-wheeling rather than coil-building, suggests that the former should perhaps be regarded as a distinct technology in its own right, rather than as a gradual transition between coil-building and wheel-throwing techniques, or vice versa. In this regard, secondary shaping with a wheel may have been performed explicitly in an attempt to imitate the visual appearance (e.g. rilling marks, even rims) of wheel-thrown vessels, by potters lacking the technological skills, or means, to practice wheel-throwing themselves.

A further feature to emerge from the SANS analysis, is the predominance of anticlockwise wheel rotation among samples identified as having been wheel-thrown (Fig. 16). Of the 21 samples identified as wheel-thrown (including samples 3 and 41), only two samples appear to have been thrown with a clockwise wheel rotation. As far as may be discerned from the limited assemblage analysed here, an apparent preference for anticlockwise wheel rotation is seen in all of the wares examined where wheel-throwing is attested. Accordingly, it is difficult, at present, to ascribe this phenomenon to any specific technological traditions. However, among contemporary potters the direction of wheel rotation has been claimed to vary according to handedness and cultural background; among Western potting traditions, right-handed potters have been reported to favour anticlockwise rotation when throwing, whereas among Far Eastern traditions they favour clockwise rotation^92^. The proportion of anticlockwise wheel rotation within the analysed archaeological assemblage (90%) agrees with the proportion of right-handedness in contemporary populations (c. 90%^93^), suggesting that handedness may perhaps also explain the pattern seen in the archaeological assemblage. However, such an interpretation needs to be viewed with caution as, as the Eastern traditions demonstrate, the preference of wheel direction may also primarily reflect technological traditions, and systems of learning and teaching, rather than individual handedness.

## Conclusions

The present study has demonstrated the utility of SANS for the quantitative and non-destructive analysis of forming techniques in ancient pottery by examining the average orientation of nanoscale scattering domains within their fabrics. For this purpose, the average orientation of the scattering domains (consisting of nanoscale particles, pores, grains, crystallites, and including nanoscale regions of larger, microscale, objects) may be inferred from the tilting angle and isotropy of the 2D anisotropic small-angle scattering of neutrons (Fig. 1).

Using a large number of samples from experimental vessels, multiple 2D SANS measurements in orthogonal planes have been combined to model the effects of different forming techniques on the average 3D orientation of the scattering domains (Fig. 8). While such 3D structural information shows clear differences between wheel-thrown and coil-based forming techniques (i.e. coil-built or coil-wheeled), in most instances these forming techniques, as well as percussion-building, may also be differentiated by SANS measurements made in the tangential plane alone (Table 3), thereby largely avoiding the need for destructive sampling. For coil-building and coil-wheeling, as both techniques show low magnitude tilting angles in the tangential plane, they may often be differentiated from each other and from other forming techniques by combining tangential plane SANS measurements with additional macroscopic evidence for secondary wheel-shaping (e.g. the absence or presence of rilling marks preserved on the interior of vessels, or evenly shaped rim, etc.). However, where it is suspected that surface wheel-traces may have been removed by later production stages (e.g. by burnishing or addition of a slip), coil-building and coil-wheeling may instead be differentiated by additional measurements made in the vertical plane, although in this case only, requiring the removal of samples of c. 10 mm × 10 mm × 10 mm. More generally, the combination of macroscopic information with SANS measurements may also be used to identify other potential composite forming techniques, such as percussion-wheeling or drawing-wheeling, which are otherwise difficult to detect from macroscopic features alone or using conventional optical microscopy of thin sections.

**Table 3:** Summary of the characteristics of primary and composite forming techniques, identified using SANS measurements in the tangential plane, based on experimental and archaeological results.

Primary forming technique	Tilting angle	Isotropy
Coil-building (CB)	0° to ± 15°	< 0.95
Drawing*	50° to 90°, or – 50° to – 90°	< 0.95
Percussion-building (PB)		≥ 0.95
Wheel-throwing (WT)	Clockwise rotation: 20° to 50°	< 0.95
	Anticlockwise rotation: – 20° to – 50°	< 0.95
**Composite wheel-techniques**		
e.g. Coil-wheeling (CW), percussion-wheeling (PW)*	Same as primary forming technique, but often with additional macroscopic surface traces indicating wheel-use	

*Drawing and percussion-wheeling were not investigated experimentally, but the characteristic features are those expected based on previous discussion in the literature and results seen from archaeological samples.

From the results of the experimental samples, it appears that sample thickness has no clearly discernible effect on either tilting angle or isotropy, at least up to a thickness of 25 mm. Accordingly, this suggests that SANS measurements may be undertaken for sherds from all but the very largest vessels (e.g. some types of pithoi). For samples of more than 25 mm thickness, multiple scattering may potentially have a detrimental effect on SANS measurements, although this has yet to be investigated systematically.

In a similar manner, the experimental samples demonstrate that firing temperature also appears to have a negligible effect on SANS measurements, at least for samples fired in an oxidizing atmosphere below 950 °C. This result appears to be linked to the level of vitrification of the samples, which for both Fabrics A and B (non-calcareous) and Fabric C (calcareous) exhibit extensive, or lower, levels of vitrification when examined by scanning electron microscopy (Supplementary Material A Figures S4 & S5). For samples showing more extensive (i.e. continuous) vitrification, it may be expected that isotropy in particular will be higher, as the fabrics lose the original structure gained during forming. Vitrification itself is linked to the proportion of fluxing materials within a fabric (most commonly calcite) and firing atmosphere (oxidising or reducing), as well as firing temperature^14,94^. Accordingly, following the criteria for the levels of vitrification described by Maniatis and Tite^94^, SANS may be expected to be suitable for calcareous fabrics fired below 1050 °C, and for non-calcareous fabrics fired below 950 °C in an oxidising atmosphere. However, due to the earlier onset of continuous vitrification, SANS may potentially be less suitable for non-calcareous fabrics fired in a reducing atmosphere above 900 °C.

The close agreement between the results from X-ray microtomography, neutron tomography, and SANS presented here (Fig. 11 and Table 2) suggests that microstructural objects and nanoscale scattering domains within pottery fabrics respond in similar manners to the forces applied during forming. In turn, therefore, this suggests that SANS may offer some advantages over X-ray and neuron imaging methods in the quantitative analysis of forming techniques, as the scattering domains of the matrix measured by SANS are found in both coarse-textured and fine-textured fabrics, whereas imaging methods are necessarily largely confined to fabrics containing comparatively large inclusions or voids.

Furthermore, taking into account potential localised structural variations seen in coil-based samples, determinations of forming techniques using SANS may be most successfully undertaken using a 10 or 16 mm diameter beam, enabling even small pottery sherds to be analysed. By contrast, samples of considerably larger size are generally required for equivalent quantitative analyses by microstructural approaches (e.g. optical microscopy of thin sections, radiography, and tomography) in order to measure the orientation of sufficient numbers of segmented objects. The ability to undertake measurements over small areas of samples using SANS, also potentially enables more complex pottery production strategies to be investigated, such as the use of different techniques to form various sections within an individual vessel.

While SANS may offer a range of advantages, in other regards it may be considered to share some of the shortcomings of microstructural approaches concerning the identification of certain subtle individual details of closely related forming techniques. This criticism is perhaps most evident in the difficulty of discriminating individual techniques within the broader category of percussion-building. Such technologically distinct forming techniques such as moulding, pinching, tamper-and-concave-anvil, etc., may be considered to be related to each other in their use of repetitive, discontinuous, pressure applied perpendicular (or near-perpendicular) to the walls of a vessel, and consequently may all be expected to result in similar patterns of object orientation (i.e. closely aligned to the walls of vessels, but very poorly aligned to the rim-plane). Similarly, these analytical techniques may also struggle to describe potentially pertinent subtleties within other categories of forming techniques, such as the specific manner in which coil-layers or pot sections may have been joined.

Notwithstanding the limitations briefly considered here, the practical application and analytical utility of SANS have been demonstrated using a large assemblage of archaeological pottery, representing a wide range of styles and including coarse and fine textured fabrics. The SANS measurements, when combined with macroscopic information, demonstrated the use of a wider range of forming techniques in the former Roman province of Pannonia during the fourth–sixth centuries AD than had previously been suspected. Whilst in agreement with earlier suggestions for a shift from large-scale pottery production using wheel-throwing to other forming techniques^91^, the increase in the number of distinct technological traditions attested should perhaps be seen in terms of the growing early medieval social and cultural diversity of the region, rather than simply the economic decline of the late Roman empire. The archaeological case study also demonstrates the comparatively high rate with which forming techniques can be identified with the aid of SANS. In this instance, forming techniques could be assigned to all of the 50 archaeological sherds investigated, notwithstanding the fact that many would otherwise be considered as ‘undiagnostic’ from the perspective of conventional macroscopic analysis.

While further methodological developments are underway, the present study has demonstrated the potential value of SANS as an aid to the analysis of the forming techniques of ancient pottery. As such, the non-destructive nature of the analysis, its suitability for fine- or coarse-textured fabrics, and the comparative speed of measurements, together with the production of objective, quantifiable, and reproducible data may be highlighted as particular advantages.

## Electronic supplementary material

Below is the link to the electronic supplementary material.

Supplementary Information 1

Supplementary Information 2.

## Data Availability

Additional data are provided in the Supplementary Materials.
