# Publisher Correction: Novel application of SANS provides quantitative non-destructive identification of forming techniques in late Roman and early medieval pottery from Pannonia

**DOI:** 10.1038/s41598-024-79319-w

**Published:** 2024-11-26

**Authors:** John Gait, Katalin Bajnok, Nicolas Hugot, Friderika Horváth, Gérard Pépy, Darren Ellis, Adél Len

**Affiliations:** 1 Neutron Spectroscopy Department, HUN-REN Centre for Energy Research, Budapest, Hungary; 2 Department of Scientific Research, The British Museum, London, UK; 3 Institute of Archaeological Sciences, Eötvös Loránd University, Budapest, Hungary; 4 Paris, France; 5 Institute of Archaeology, HUN-REN Research Centre for Humanities, Budapest, Hungary; 6 Institute of Making, University College London, London, UK; 7 Faculty of Engineering and Information Technology, University of Pécs, Pécs, Hungary

Correction to: *Scientific Reports* 10.1038/s41598-024-77426-2, published online 29 October 2024

In the original version of this Article Gérard Pépy was incorrectly affiliated with ‘ENSTA Paris, Institut Polytechnique de Paris, Palaiseau, France’. The correct affiliation is listed below.

Paris, France.

The original Article has been corrected.

