# Supplementary material for: Novel application of SANS provides quantitative non-destructive identification of forming techniques in late Roman and early medieval pottery from Pannonia
Source: Sci Rep. 2024 Oct 29;14:25926. doi: 10.1038/s41598-024-77426-2 (PMC11522291; doi:10.1038/s41598-024-77426-2)
Supplement: Supplementary file 1 — Supplementary Information 1 [file 41598_2024_77426_MOESM1_ESM.pdf]

Supplementary Material A: Notes S1-S4 and Figures S1-S26

Gait, J., Bajnok, K., Hugot, N., Horváth, F., Pépy, P., Ellis, D. & Len, A. (2024). Novel application of SANS provides quantitative non-destructive identification of forming techniques in late Roman and early medieval pottery from Pannonia

Note 1: NSXY v. 2022

The NSXY software has been designed especially for the 2D data evaluation of anisotropic small-angle neutron scattering (SANS) from archaeological pottery.

<u>Characteristics of the software</u>

- Deals with elliptical scattering shapes.
- Uses a "pre-processing" algorithm based on a geometrical approximation to determine the initial parameters for the iterative model fitting process, making possible the simultaneous reading and evaluation of hundreds of measured and calibrated data files in a very short time.
- Uses the least-squares model fitting method to find the final parameters of the power-law model that describes the data.
- Calculates the uncertainties of the fitted parameters.

<u>Basic features</u>

- Browse file system and load 64 × 64 data files from the YS-SANS instrument at Budapest Neutron Centre, calibrated using the BerSANS data reduction software[1]
- Visualize the measured 2D data: display results on a 1D and 2D graph
- Process data files either individually or as a batch, and export graphs as image files, and save numerical pre-processed results, final results, uncertainties and the χ^2values in a text file

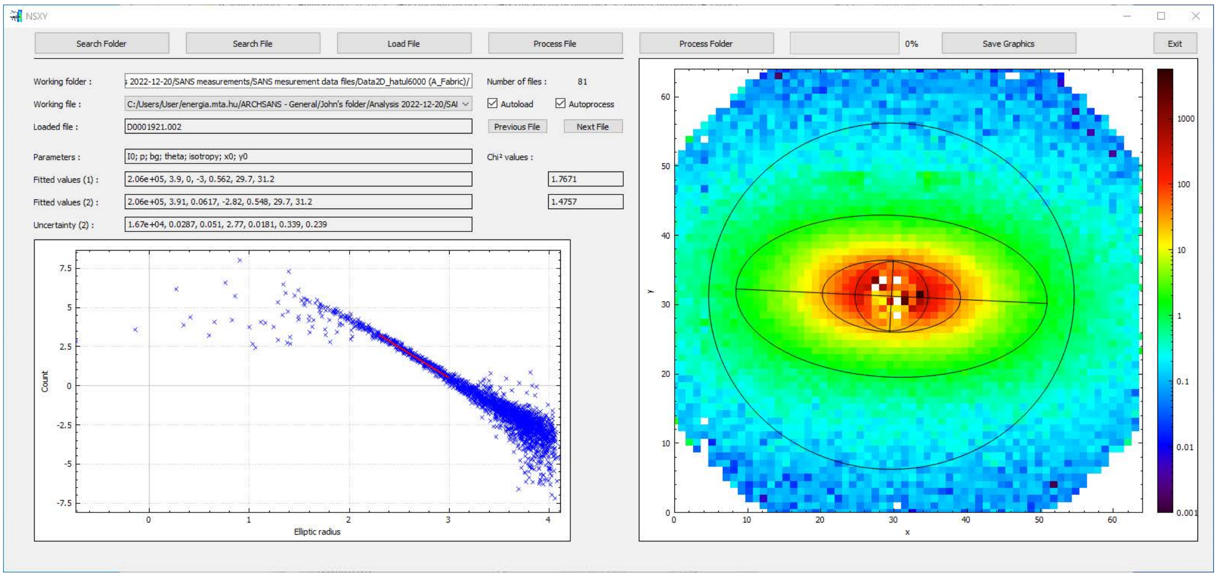

Supplementary Material A Figure S 1: User interface of the NSXY software

Data evaluation procedure

- Pre-processing
  - Using a geometry related algorithm[2], determines the following geometrical parameters, which are used as entry parameters for the least-square fitting:
    - θ – tilting angle
    - γ – isotropy
    - x_0, y_0 – centre of the direct neutron beam on the detector

- Least square fitting
  - Uses a standard optimization algorithm, based on minimising the χ^2, which quantifies the difference between the observed data and the values predicted by the model
  - Used model: power law

$$I(Q_{x,}Q_y) = \frac{I_0}{\sqrt{\Delta x_\theta^2 + \gamma^2 \Delta y_\theta^2}^{\,p}} + bg$$

$\Delta x_\theta^2 = (x - x_0)cos\theta + (y - y_0)\,sin\,\theta$
$\Delta y_\theta^2 = -(x - x_0)\,sin\,\theta + (y - y_0)\,cos\,\theta$
$I_0 -$ intensity
x_0 – beam centre x coordinate
y_0 – beam centre y coordinate
γ – isotropy
p – power
θ – tilting angle
bg – background noise

- Data processing

Let $\vec{\xi} = (I_0, p, bg, \gamma, \theta, x_0, y_0)$ be the parameter vector. For each pixel $i \in [\![1, N]\!]$, given ν_i the neutron count, σ_i its standard error and its coordinates x_i and y_i, the maximum likelihood estimator is found by minimising the following weighted mean-square distance over the parameter space:

$$\chi^2(\vec{\xi}) = \frac{1}{N}\sum\left(\frac{I(\vec{\xi}; x_i, y_i) - \nu_i}{\sigma_i}\right)^2$$

Technical specifications

- Programming language: C++14 (on Ubuntu 20.04 LTS)
- Built for: Microsoft Windows 10 Professional (MinGW)
- External libraries: NLopt, Qt 5.15, QCustomPlot

Validation of the software

The software was validated with random testing and by comparison with the reference software HWXY[3].

Availability of NSXY

https://bnc.hu/ys-sans/

Contributions and acknowledgments

The software project was based on an original idea by AL, with further design and development by AL, GP and NH. The software was written by NH. Testing and evaluation were undertaken by NH, AL, JG and KB. Financial support was provided by the Budapest Neutron Centre Internship Program and HUN-REN Proof-of-Concept program: ELKH-PoC-2022-029.

Note 2: Corrections for non-perpendicular alignment of measurement locations

Owing to the angled or curved nature of the walls of the experimental and archaeological vessels, the location point of each measurement was often not aligned perpendicular to the axis of the neutron beam. As a result of a foreshortening effect[4], the slope of the surface of the sample at the measurement location relative to the axis of the neutron beam had the effect of reducing the measured tiling angle, α. In order to compensate for this effect, and to determine the maximum tilting angles, corresponding measurements made perpendicular to the beam, a correction was applied to all measurements (where measurements were made perpendicular to the beam, as with the cube samples, the correction did not change the original value of the tilting angle). Corrections were calculated in two separate ways:

1) A trigonometric correction. The corrected tilting angle was calculated as the projection of the original tilting angle when the plane of the tangent to the measurement location was elevated from an inclined position to a position perpendicular to the beam and parallel to the plane of the detector.

2) A correction using a propagation along a sine wave. Following the results of the cube measurements, the tiling angle in the horizontal view was assumed to be zero for all forming techniques, and that as the slope of the measurement location approached the extremes of 0° or 180°, it could be considered as a measurement made in the horizontal view. It was also assumed that the tilting angle would be at its maximum when the tangent to the measurement location was perpendicular to the axis of the neutron beam (i.e. a slope of 90°).

In practice, the slope of the samples was usually greater than 60° from the axis of the beam, and appears not to have a significant effect on the tilting angle. Both correction methods give similar results (generally with 1° or 2° of each other). By default, values presented in this work are corrected using the trigonometric correction (1).

Note 3: Additional information concerning experimental samples and forming techniques

Fabric A and B experimental vessels

The initial series of experimental samples were made at the Institute of Making, University College London, by Darren Ellis, an experienced professional potter, with assistance from Sara Brouwer and Beth Munroe. Two commercial potting clays were used: Doble Standard Stoneware (Fabric A, buff firing) and Tiranti Terracotta Clay (Fabric B, red firing), both low/non-calcareous, medium/fine-textured clays containing predominantly well-rounded medium sand- to silt-sized quartz inclusions. Prior to forming, the raw clay was prepared by wedging and kneading by hand. Approximately 600 g of raw clay were used for each vessel. Aside from general descriptions of the desired approximate size and shape of the vessels, and the range of forming techniques to be used, no detailed instructions or limitations were given regarding how the vessels should be made.

The wheel-thrown vessels were made on an electric potter's wheel by a right-handed potter, with the wheel rotating in an anti-clockwise direction. The potter was able to vary the rotational speed of the wheel, as well as the speed at which the clay was lifted, according to his own self-determined requirements. For centring the clay, the wheel rotated at c. 192 rpm, whilst during the shaping of vessels a speed of c. 96 rpm was used.

For coil-building (including the initial forming of coil-wheeled vessels), long cylinders ('coils') of clay were prepared by hand by simultaneously compressing and rotating in a reciprocal manner on a flat surface masses of prepared clay, until the coils reached a diameter of c. 1.4 cm. A simple round slab of clay was used for the base of each vessel, onto which a length of coil, forming a ring, was pressed. Two additional layers of coils were then applied, and pressed onto the preceding layer. All three coil layers, and the base, were further joined to each other by gentle pinching and pressing, and smearing the outer surfaces in an approximately vertical direction. A further three coil layers were then applied and joined in the same manner.

For coil-wheeling, vessels initially prepared by coil-building were further modified using an electric wheel rotating in an anti-clockwise direction at c. 60 rpm.

After forming, the vessels were left to dry at room temperature, before being cut vertically to form vessel-quarters. Quarters from each vessel, and of both Fabrics A and B, were fired to either 650, 800, or 950°C, in an oxidising atmosphere, in an electric kiln using a predefined standard firing regime. This firing regime had previously been devised to achieve even firing throughout vessels, whist minimising risks of failure associated, in particular, with inadequate drying. The stages of each firing were as follow:

Heating from ambient to 500°C at 35°/h

Soak for 20 min. at 500°C

Heat at 120°/h to maximum temperature

Soak for 30 min. at maximum temperature

Kiln turned off and allowed to cool naturally to ambient temperature over 1½ - 2 days

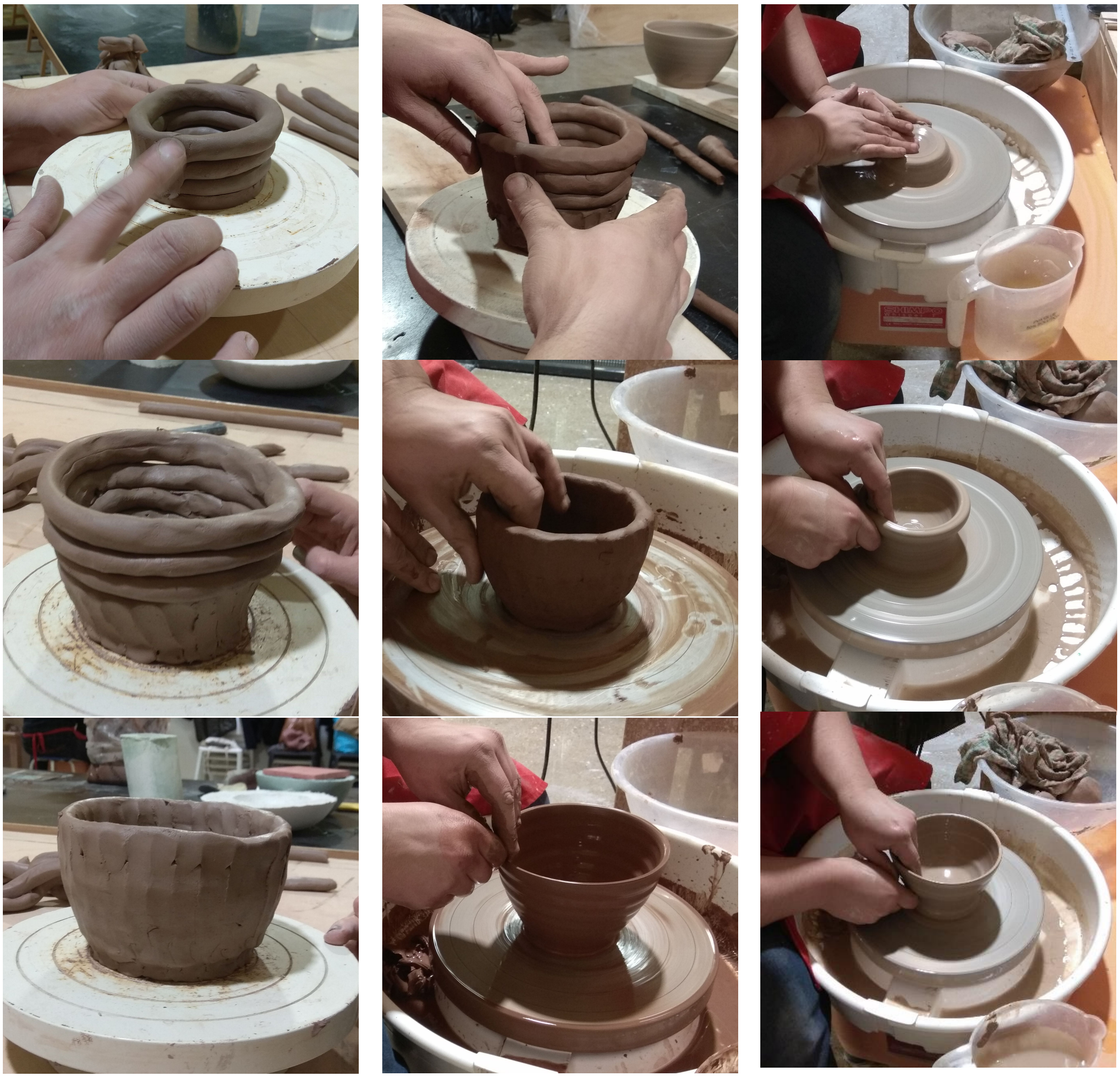

Coil-building **Coil-wheeling** **Wheel-throwing**

Supplementary Material A Figure S 2: Stages of production of Fabric A and B experimental vessels

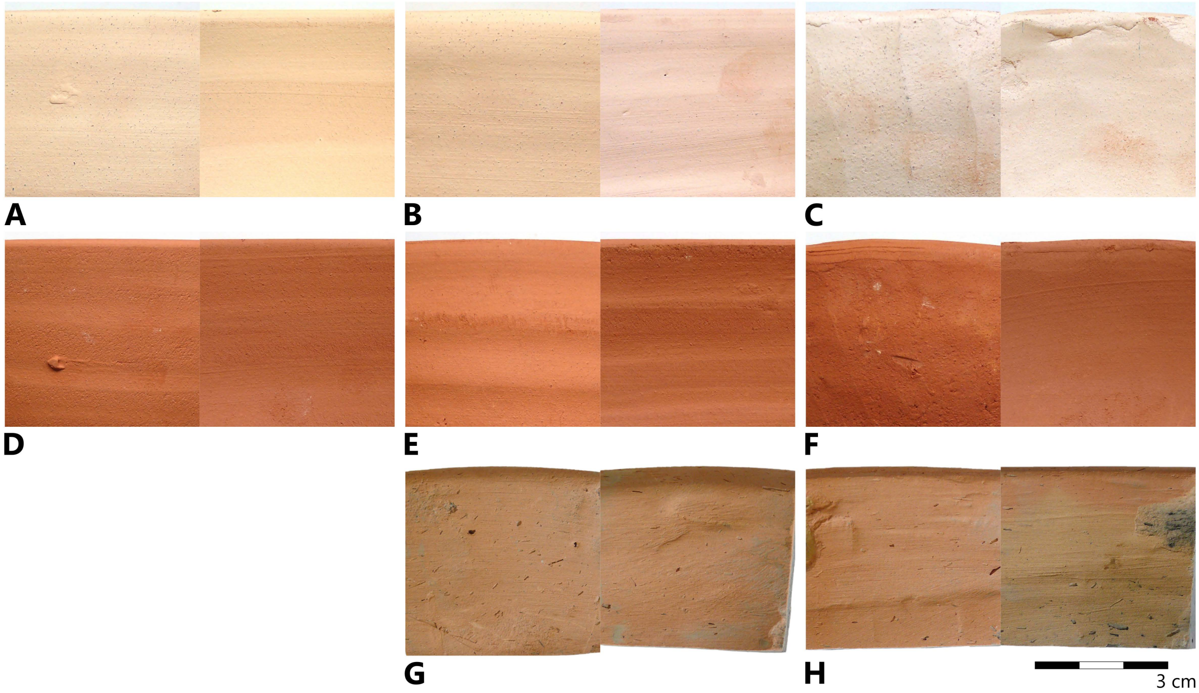

Supplementary Material A Figure S 3: Exterior (left) and interior (right) surfaces of representative samples from experimental vessels of Fabrics A (top row), B (middle row), and C (bottom row); **A & D:** wheel-thrown; **B & E:** coil-wheeled; **C, F & H:** coil-built; **G:** percussion-built. Wheel-thrown and coil-wheeled vessels both show fine rilling marks and undulations slightly inclined relative to the rim, and are difficult to distinguish macroscopically. Coil-built vessels show comparatively rough exterior surfaces with faint, near-vertical ridges formed by the smudging of successive coil-layers. Percussion-built vessels show occasional shallow depressions on surfaces, but are difficult to consistently distinguish from coil-built vessels.

Terminology:

Within the archaeological literature, a variety of different terms have been used to describe pottery forming techniques. With the hope of avoiding potential ambiguity, the meaning of certain terms, as used by the authors, are further clarified here:

- Coil-based: a collective term referring to forming techniques using coiling-building as the primary forming technique, i.e. coil-building (CB) and coil-wheeling (CW).

- Coil-building (CB): a primary forming technique where a vessel is formed using long cylinders of clay ('coils') placed, either in a rising spiral or as horizontal layers, to form the initial, or final, desired shape and height of the vessel. Successive coil layers are pressed together, and further joined together by smearing. The coils themselves are usually formed by pressing and rolling lumps of clay against a flat surface or between the palms of the hands, or alternatively they may be formed by roughly squeezing by hand (particularly for large coils), or by extrusion with a suitable mechanical device (as used by modern potters, but not applicable to ancient pottery technology). In some instances, coils and elongated slabs may be difficult to differentiate from each other, both in their physical appearance as well as conceptually[5].

- Coil-wheeling (CW): a composite forming technique, where a vessel is formed first using coil-building (primary forming), before being further modified to varying extents using a rotational device (e.g. potter's wheel or turntable; secondary forming), typically resulting in a decrease of wall thickness, alterations to the surfaces of the walls, and minor to more significant changes in the overall vessel shape. This specific combination of primary and secondary forming techniques is often referred to in the literature as 'wheel-shaping' or 'wheel-fashioning'[6], although Roux later also uses 'wheel-fashioning' to denote an overarching category that includes both 'wheel-coiling' and wheel-throwing[7]. Notwithstanding such ambiguity, the former terms may be considered unhelpful, or potentially misleading, in that they overly emphasise the role of the secondary forming to the total exclusion of the primary forming. While 'wheel-coiling' is certainly an improvement in this regard, the authors of the present article suggest that 'coil-wheeling' is more apt, in that it reflects the relative order in which the forming techniques are executed. In the present article, this latter system of nomenclature is also applied to other potential composite forming techniques e.g. 'percussion-wheeling'.

- Drawing: a primary forming technique in which a vessel is formed by simultaneously squeezing clay between fingers and thumb, or between hands, and pulling upwards[8]. The technique may be applied to a lump of clay in which an opening has been first made, or for large vessels, used in combination with coil- or slab-building but in which the height of the vessel is increased by the drawing action (in contrast to the similar action of smudging used to join successive layers during coil-building).

- Hand-building (HB): a collective term for all primary and secondary forming techniques that do not use a rotational device (e.g. potter's wheel or turntable). Accordingly, for example, vessels made using coil-building alone, or coil-building followed by secondary beating, may be described as hand-built. In contrast, vessels made by coil-building followed be secondary wheel-shaping, may be described as coil-wheeled or wheel-made, even though all the vessels were made with the same primary forming technique.

- Percussion-building techniques (PB): a collective term for various primary forming techniques that use repetitive intermittent pressure, e.g. slab-building, pinching, moulding, and tamper-and-concave-anvil[8,9]. While these individual techniques represent distinct technological practices, differentiated by a range of factors, such as the types and manners of tools used or the types/sizes of vessels for which they are applied, they may be more difficult to differentiate in the archaeological

record. From the perspective of microstructural, or nanostructural, studies they may all be expected to result in a similar pattern of object orientations. Similar (or identical) tools and actions to those used for primary forming, may also be applied during secondary forming, e.g. beating[8], although significantly, without necessarily destroying the structure of the fabric resulting from the primary forming stage.

- Wheel-made: a collective term referring to forming techniques utilising a rotational device (e.g. potter's wheel or turntable) for primary or secondary forming, e.g. wheel-throwing, coil-wheeling, percussion-wheeling. Accordingly, a coil-wheeled vessel may be categorised as both coil-based and wheel-made.

- Wheel-shaping: the secondary use of a rotational device (e.g. a potter's wheel or turntable) to modify a previously formed vessel, usually made without the use of a wheel for primary forming (i.e. hand-built). Significantly, the application of wheel-shaping is not constrained by any specific type of preceding primary forming technique. Accordingly, the meaning suggested here differs slightly from those instances where it has been assumed that only coil-building was used for primary forming, and where consequently 'wheel-shaping' has been used as a synonym for the coil-wheeling technique specifically.

- Wheel-throwing (WT): a primary forming technique in which a vessel is formed by lifting a mass of plastic clay upwards from the head of a potter's wheel as it is simultaneously rapidly rotated around the central axis of the wheel. Wheel-throwing itself employs multiple specific individual techniques[8] or operations, including centring and opening, as well as lifting. While the term 'fast-wheeled' is often used to denote wheel-throw vessels, in contrast to 'slow-wheeled' (i.e. the secondary modification of a vessel using a rotatory device, after initial forming by some other technique), the critical importance of rotational speed for differentiating forming techniques that these terms imply has been questioned.[10,11]

Note 4: Additional description of archaeological sites

Keszthely-Fenékpuszta (Zala County)

Keszthely-Fenékpuszta is a Roman fortress along the shore of Lake Balaton, behind the Danube *limes*, which was built in the mid-4th century, and which played an important role also in the civilian administration of the Late Antique Pannonia.[12] The square-shaped fortification, covering an area of 15 hectares, was surrounded by ca. 2.2 m thick walls with 44 outward-facing circular towers, and could be entered through four gates located in the central axes of the walls. So far 29 buildings have been identified within the walls, including a principal building, baths, a *gymnasion*, an early Christian basilica, granaries (*horrea*), and other agricultural/utility buildings.[13]

Following the end of the Roman administration of the provinces *Pannonia Prima* and *Valeria* (ca. mid-5th century AD), Keszthely-Fenékpuszta remained populated continuously until the 7th century[14,15], and developed into a local centre in the region.[16] From this perspective, it represent one of the most important sites in Hungary where there appears to be a continuity of occupation across the Roman and the early Medieval periods.

Archaeological investigations of the fortress started at the end of the 19th century and continue to this day.[17] More recently, a German-Hungarian collaboration between 2006 and 2009, supported by the Deutsche Forschungsgemeinschaft (DFG) and the Hungarian Academy of Sciences (MTA), re-evaluated the results of the earlier excavations up to 2002, and in addition, carried out an extensive geophysical survey and other scientific analyses (i.e. anthropological, strontium isotope and radiocarbon analysis). From 2009 to 2017, a new German-Hungarian collaboration has been formed to further excavate the fortress and evaluate old excavation results, led by Orsolya Heinrich-Tamáska (Leibniz Institute for the History and Culture of Eastern Europe, GWZO), Roland Prien (University of Heidelberg), Péter Straub (Balatoni Museum) and Bálint Havasi (Göcseji Museum).[13]

Selected ceramic materials from the 4th – 5th century, from the excavations up to 2002, were published by Friderika Horváth.[18–20] In the present study, pottery sherds from Buildings 4 (*horreum*), 24 (baths), 25 (principal building) and 27 (*gymnasion*) are analysed, recovered during the recent excavation campaigns (2009 to 2017).

Ordacsehi, Kis-töltés (Somogy County)

The rescue excavations of the M7 motorway on the southern shore of Lake Balaton in 2001 revealed a settlement with Roman and Germanic features in Ordacsehi. The excavations were carried out by the Institute of Archaeology of the Hungarian Academy of Sciences on behalf of the Directorate of Museums of Somogy County, led by Gabriella Kulcsár, during which an area of 21 500 m^2 was excavated.[21] Among the excavated features, 7 houses, 21 pits (including features & material relating to bronze working[22]), an oven, and 4 burials were assigned to the late Roman/Early Medieval period. The materials connected to these features – mainly consisting of ceramics – were studied by Zsófia Bocsi.[23,24] Preliminary petrographic studies on the pottery were published by Katalin Bajnok.[25] Based on the stylistic analysis of the excavated material, Zsófia Bocsi suggested that the settlement was in use in the first half of the 5th century AD.[26]

Zamárdi, Kútvölgyi-dűlő (Somogy County)

The rescue excavations of the M7 motorway on the southern shore of Lake Balaton in 2002 revealed a settlement with Roman and Germanic features in Zamárdi. The excavations were carried out by the Institute of Archaeology of the Hungarian Academy of Sciences on behalf of the Directorate of Museums of Somogy County, led by Viktória Kiss and Péter Polgár, during which an area of 32 300 m^2 was excavated.[27] Among the excavated features, 10 houses, 14 workshops (?), 13 baking ovens, 3 wells and 6 burials were assigned to the late Roman/Early Medieval period. A unique feature of this site is that a complete skeleton of a lynx was found in one of the pits, along with the skeleton of several dogs.[28] The materials connected to these features – mainly consisting of ceramics – were studied by Zsófia Bocsi.[23,24,29] Preliminary petrographic studies on the pottery were published by Katalin Bajnok.[25] Based on the stylistic analysis of the excavated material, Zsófia Bocsi suggested that the settlement was in use between the second half of the 5th and the second third of the 6th century AD.[24] This dating is further supported by the radiocarbon analyses of the animal skeletons, which are dated to 430–570 AD.[30]

Szólád, Kertek mögött (Somogy County)

In 2003, a burial dated to the Langobard period (6th century AD) was found in Szólád during the rescue excavation along the M7 motorway.[31] Two years later, in 2005, an international, Hungarian-German collaborative project was launched led by Tivadar Vida (Institute of Archaeology of the Hungarian Academy of Sciences and Institute of Archaeological Sciences of the Eötvös Loránd University), Uta von Freeden and Daniel Winger (Romano-Germanic Commission of the German Archaeological Institute) to fully excavate the cemetery. Between 2003 and 2010, a total of 45 graves were uncovered, with which the cemetery can be considered fully excavated.[32]

Since the beginning of the excavations, particular attention was paid to modern scientific investigations,[33] including archaeogenetics, anthropological, N, C and Sr isotopic studies,[34,35] and petrographic and geochemical analysis of the ceramic materials.[36] According to both the stylistic evaluation of the finds and the radiocarbon dating, the cemetery can be dated to the middle third of the 6th century and was probably in use for 20-30 years.[35]

Szeleste, Vasút mellett (Vas County)

In 2013, during the construction of the M86 motorway between Szeleste and Csorna, a cemetery with 112 burials dated to the Langobard period (6th century AD) was excavated by the archaeologists of the Savaria Museum, led by Ildikó Katalin Pap.[37] Despite the fact that much of the cemetery was robbed probably as early as the 6th century, the remaining grave goods (e.g. various weapons, buckles) and some graves covered with Roman *tegulae* are indicative of a relatively wealthy population.[38] Both the material culture and the burial practices, as well as the preliminary anthropological results, suggest a mixed population reflecting late antique ("Mediterranean") and Germanic ("Nordic") cultural influences and anthropological characteristics.[39] The processing and evaluation of the cemetery is currently ongoing.

Additional figures:

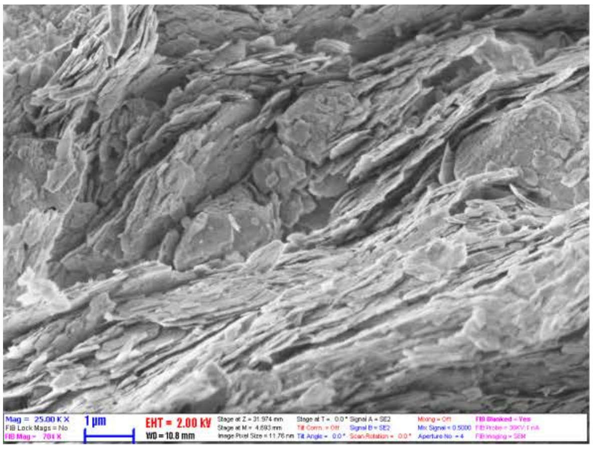

A28

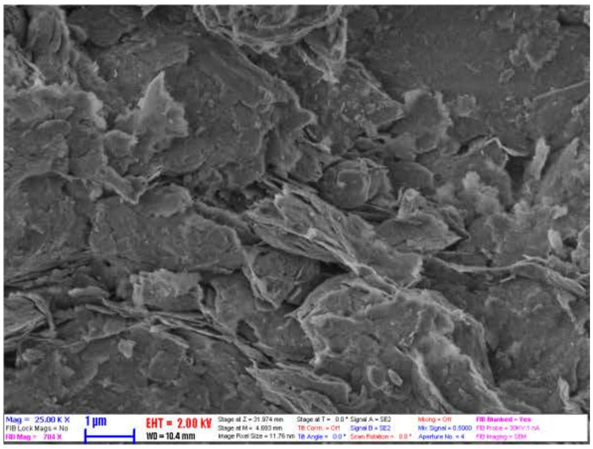

B28

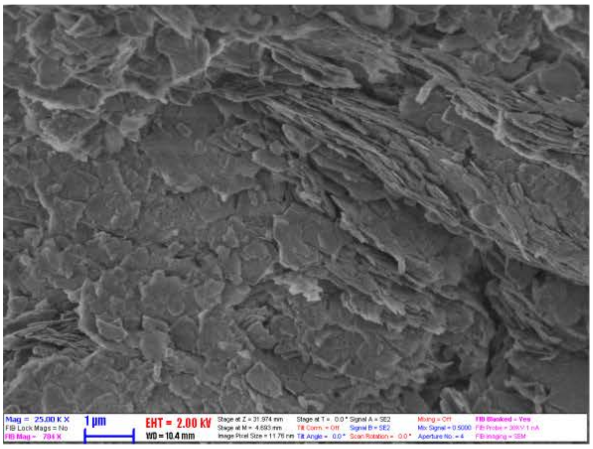

A31

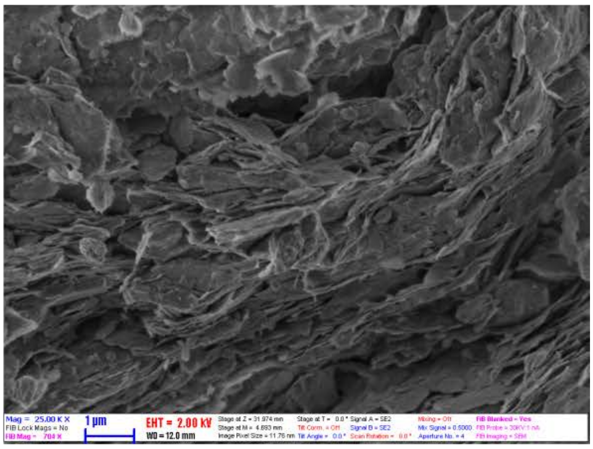

B31

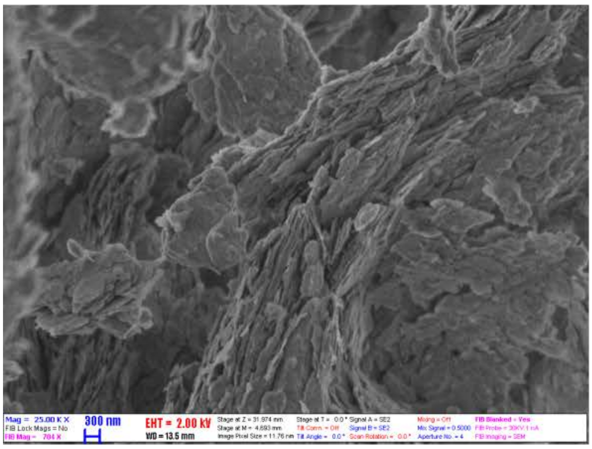

A34

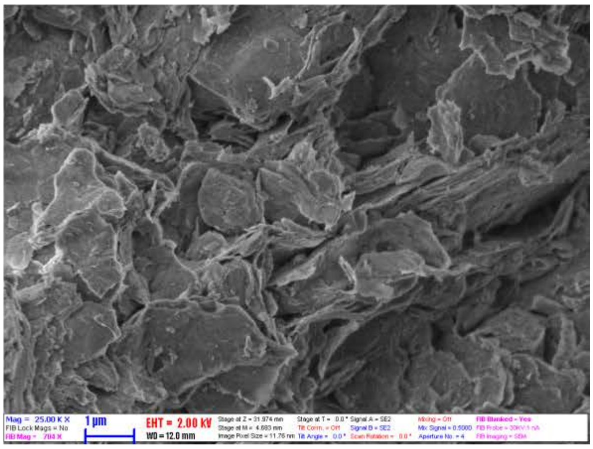

B34

Supplementary Material A Figure S 4: SEM secondary electron images (× 25K) of experimental pottery Fabrics A and B, fired at 650 (A28, B28), 800 (A31, B31), and 950 °C (A34, B34). The foliated, platy appearance of the clay minerals is apparent. The surfaces imaged were prepared as fresh-breaks, randomly orientated, and without coatings.

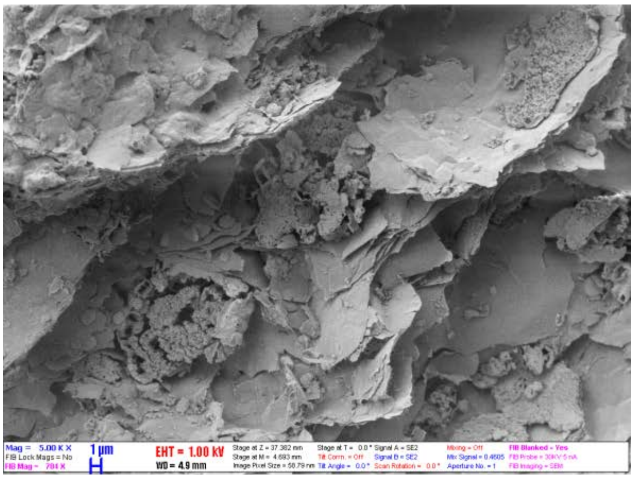

C6

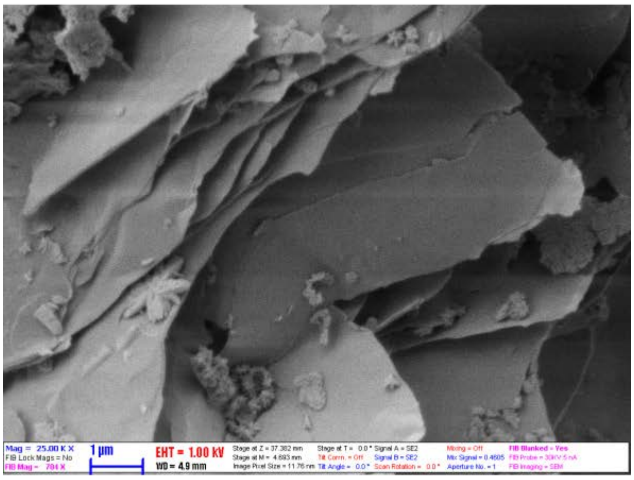

C6

Supplementary Material A Figure S 5: SEM secondary electron images (× 5K, top, and × 25K, bottom) of experimental pottery Fabric C, sample C6, fired at 700 °C. The foliated, platy appearance of the clay particles is also apparent, but of a larger size range than those of Fabrics A and B. The surfaces imaged were prepared as fresh-breaks, randomly orientated, and without coatings.

Experimental vessels:

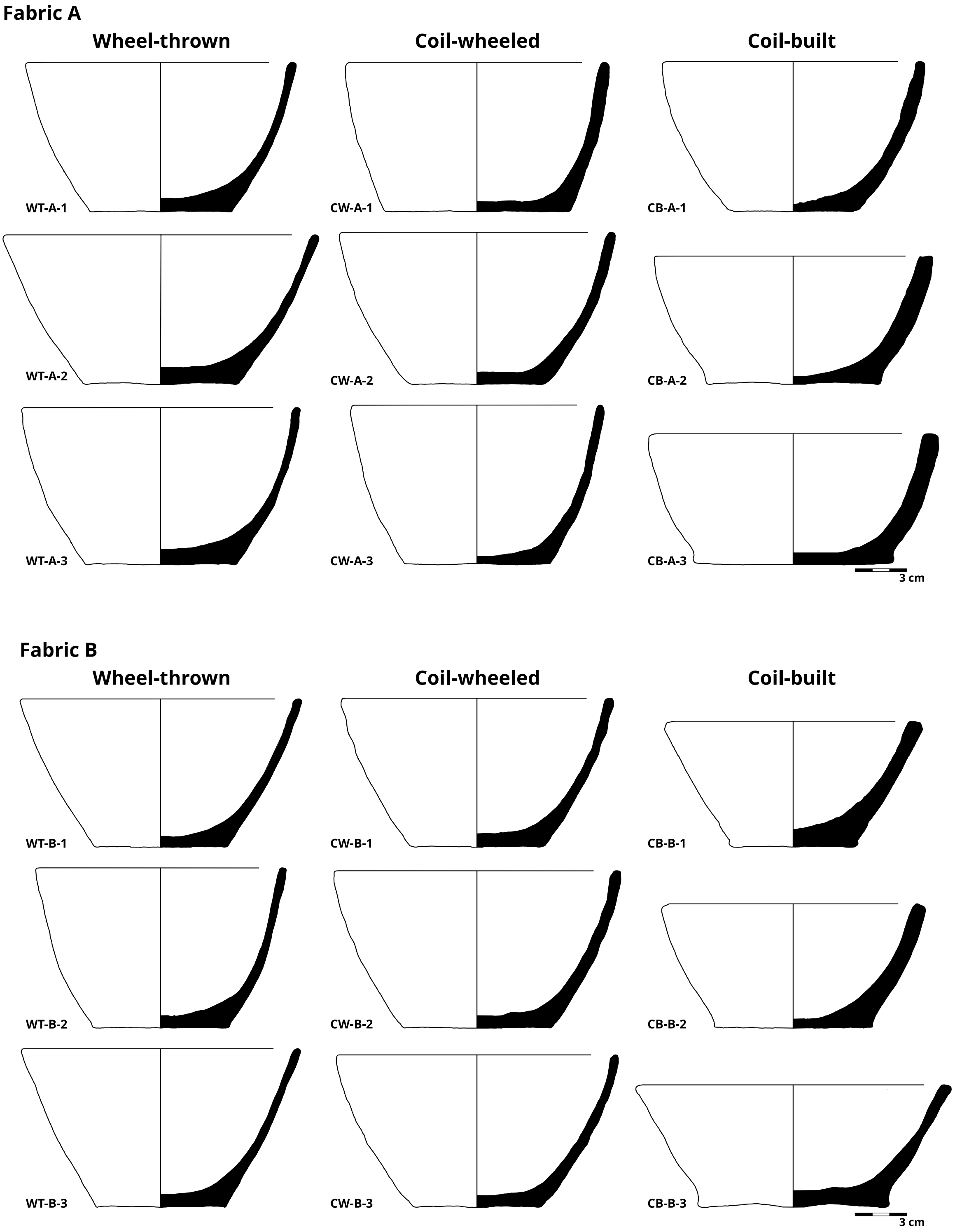

Supplementary Material A Figure S 6: Profile drawings of experimental pottery vessels of Fabric A and B.

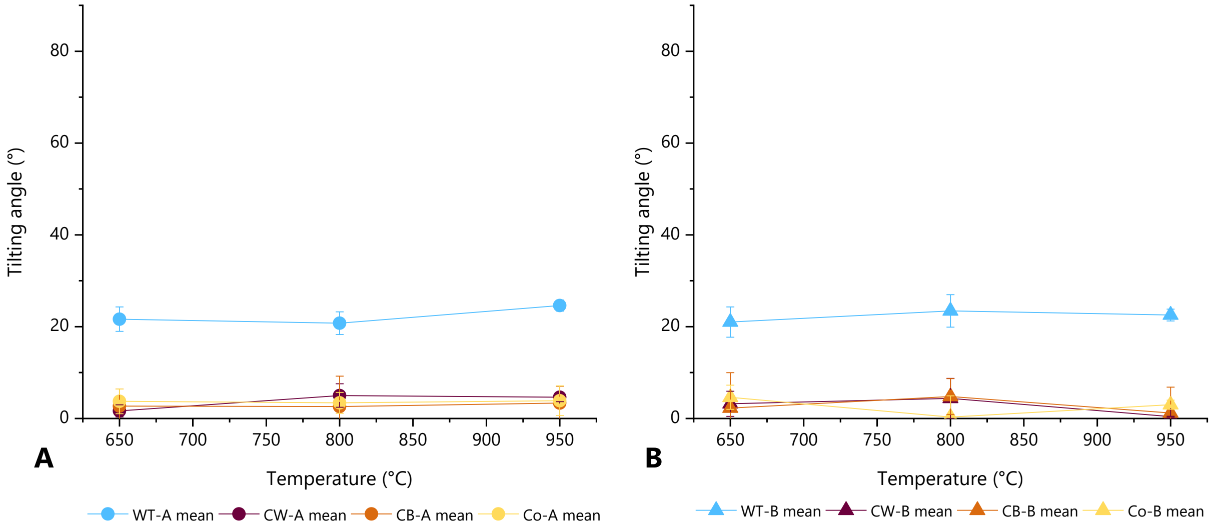

Supplementary Material A Figure S 7: Charts showing mean tilting angle against temperature for cube samples of Fabrics A and B. The firing temperature of the samples appears to have little effect on the mean tilting angle, below 950 °C (spread = ±1 SD)

Supplementary Material A Figure S 8: Interactive model of the average orientation and relative elongation of scattering domains in 3D space for coil-section samples of Fabric A. For this model, the defined coordinate axes may differ from the default axes shown in some PDF-viewers. This 3D model is best viewed using Adobe Acrobat Reader. To view the model, enable multimedia and 3D content, and click the centre of page to activate the model

Supplementary Material A Figure S 9: Interactive model of the average orientation and relative elongation of scattering domains in 3D space for coil-built samples of Fabric A

Supplementary Material A Figure S 10: Interactive model of the average orientation and relative elongation of scattering domains in 3D space for coil-wheeled samples of Fabric A

Supplementary Material A Figure S 11: Interactive model of the average orientation and relative elongation of scattering domains in 3D space for wheel-thrown samples of Fabric A.

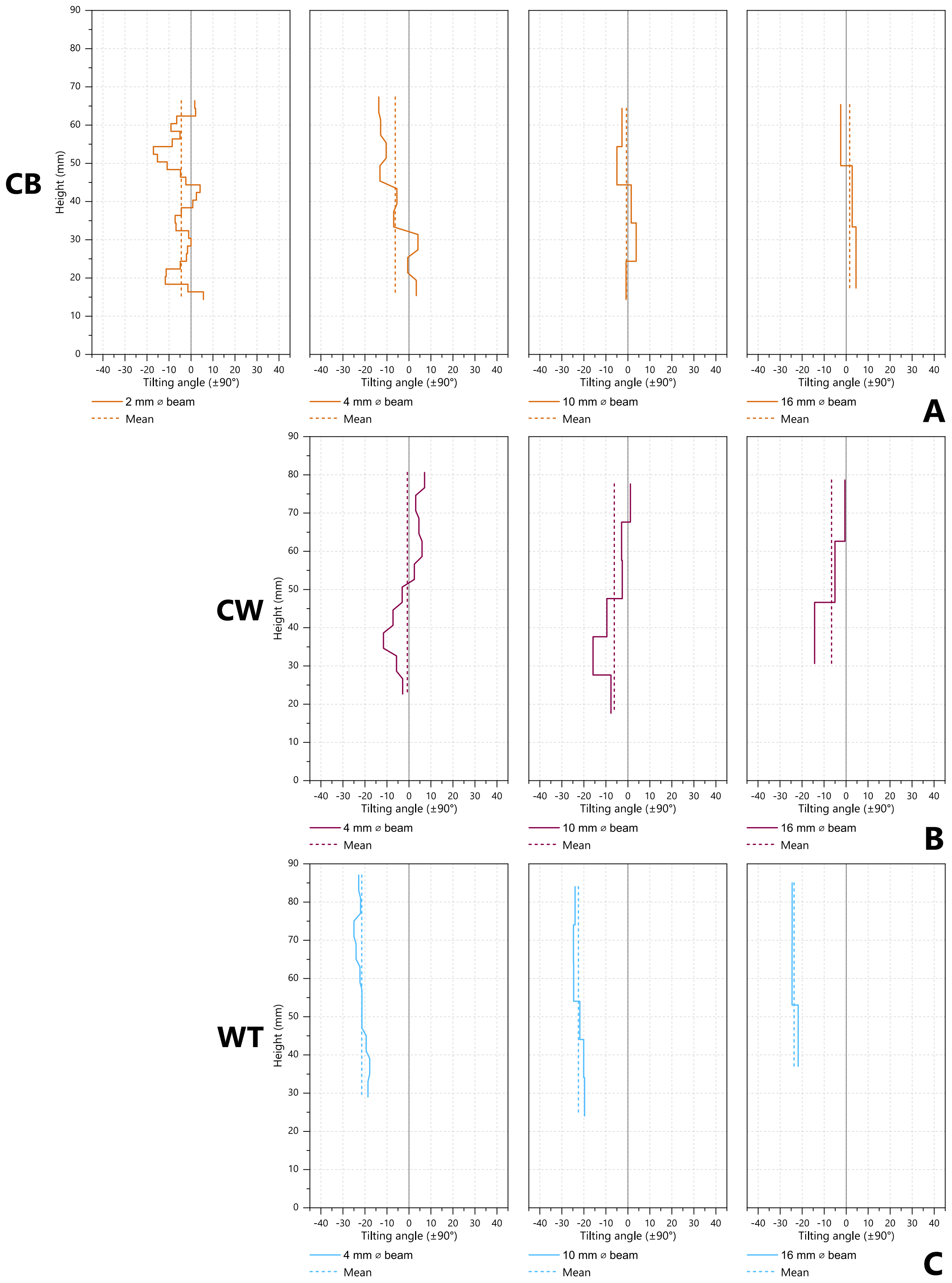

Supplementary Material A Figure S 12: Tilting angle, α_{Tan}, measured at multiple locations across vertical transects of vessel-quarters of Fabric B, using various beam diameters; mean values shown as dotted lines. A: coil-built sample B31; B: coil-wheeled sample B19; C: wheel-thrown sample B8.

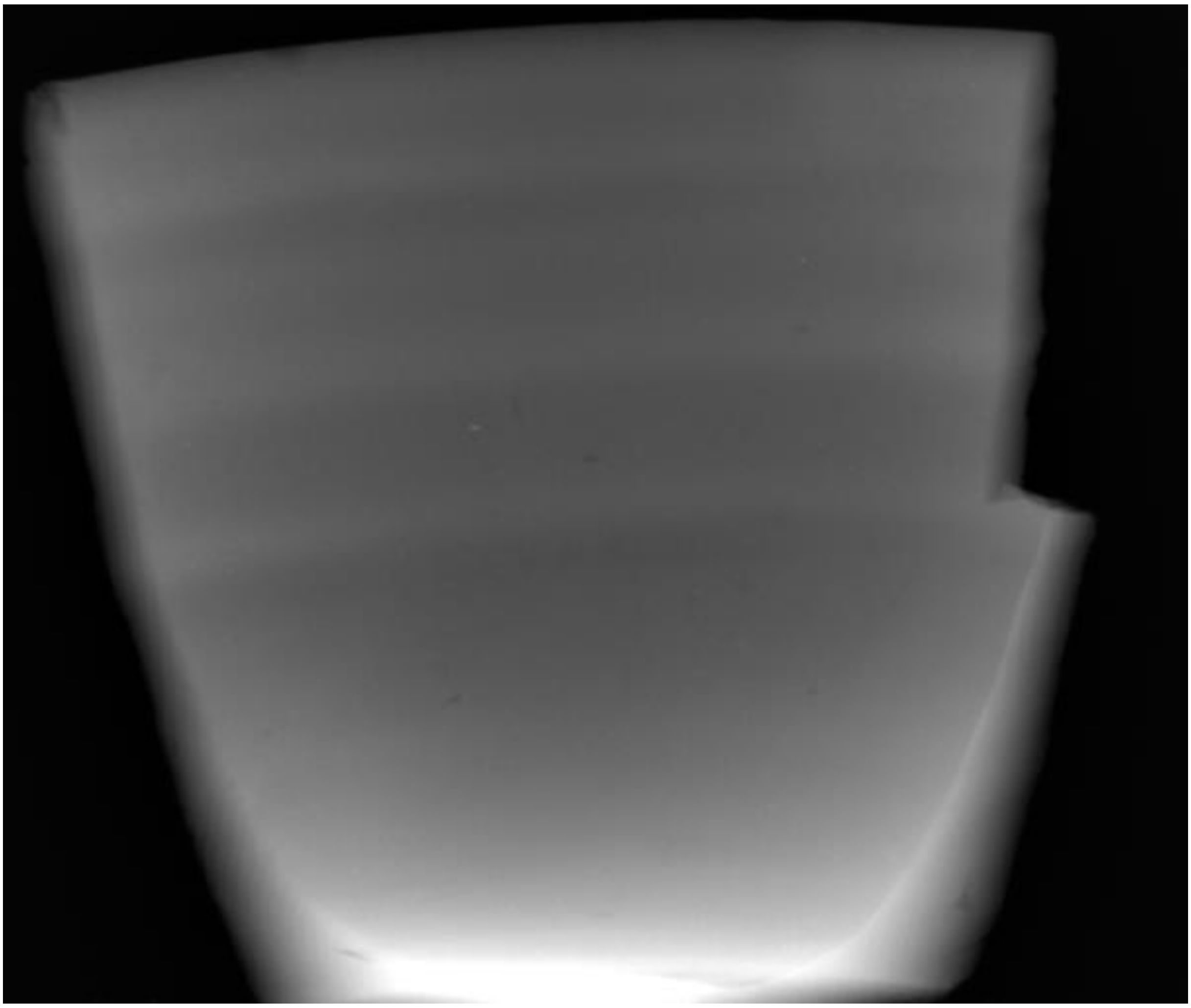

Supplementary Material A Figure S 13: Radiograph of Fabric A, coil-wheeled vessel-quarter (A19). No horizontal or vertical coil-joins are discernible between any of the six layers of coils used to form the vessel.

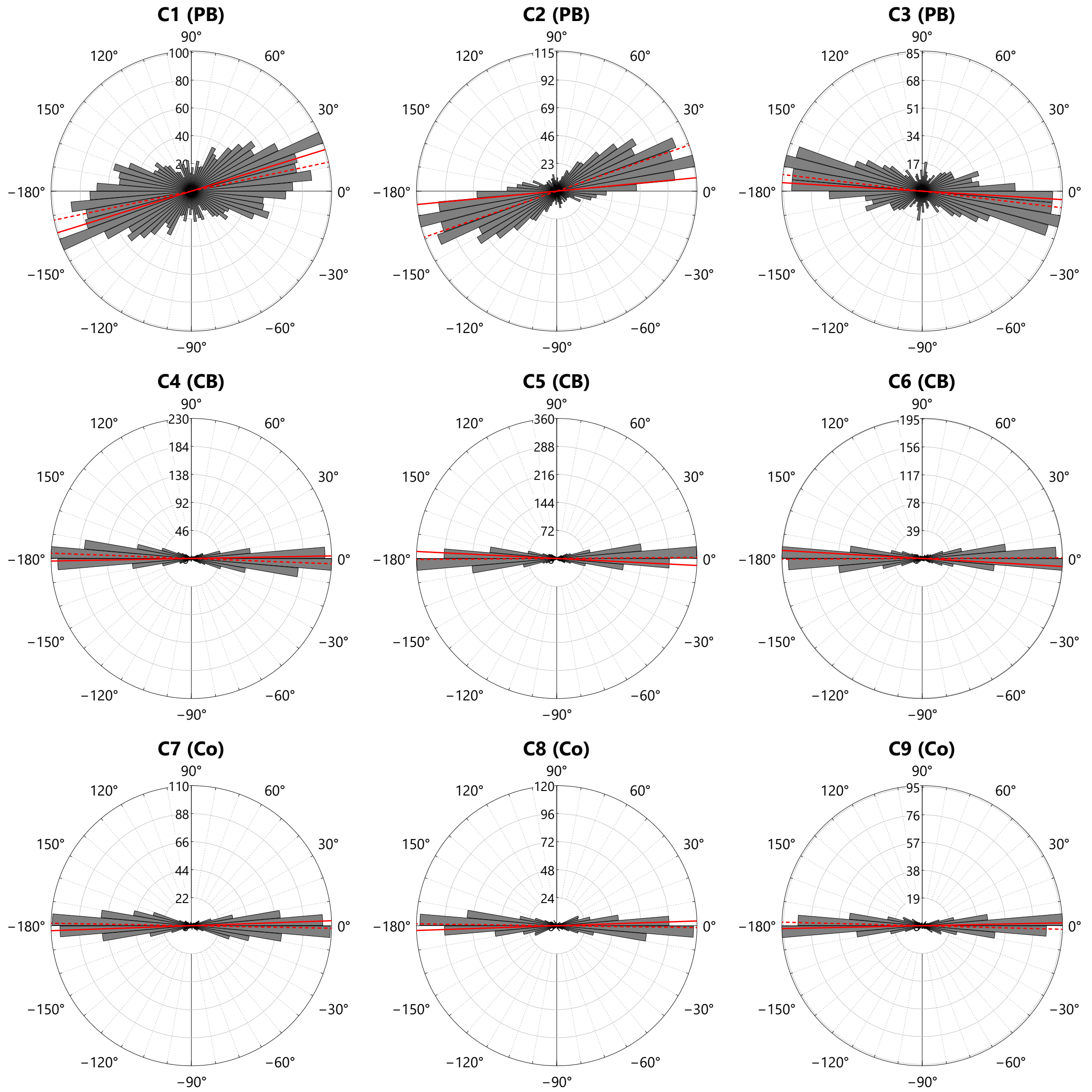

Supplementary Material A Figure S 14: Circular frequency distribution histograms of the tangential 2D projected tilting angles of segmented objects from neutron tomography (NT) analysis of Fabric C samples, $\boldsymbol{\alpha_{Tan(NT)}}$. The mean NT tilting angles, $\boldsymbol{\overline{\alpha}_{Tan(NT)}}$, and corresponding SANS mean tilting angles, $\boldsymbol{\overline{\alpha}_{Tan(SANS)}}$, are shown as dashed and solid red lines respectively. The projected tilting angles are calculated from the 3D polar and azimuth angles of each segmented object with a volume > 0.03 mm^3 and an aspect ratio < 0.3. In both SANS and NT measurements, the coil-built and coil-section samples display low mean tilting angles; in addition, the NT data show low dispersion, comparable to the relatively low SANS isotropy values (Table 4). By contrast, the percussion-built samples display considerably greater dispersion, comparable to the high SANS isotropy values.

Supplementary Material A Figure S 15: Interactive 3D schematic model of coil-section / coil-building (not to scale). For this model, the defined coordinate axes may differ from the default axes shown in some PDF-viewers. This 3D model is best viewed using Adobe Acrobat Reader. To view the model, enable multimedia and 3D content, and click the centre of page to activate the model

Supplementary Material A Figure S 16: Interactive 3D schematic model of coil-wheeling (not to scale)

Supplementary Material A Figure S 17: Interactive 3D schematic model of wheel-throwing (not to scale)

Supplementary Material A Figure S 18: Interactive 3D schematic model of percussion-building (not to scale)

Supplementary Material A Figure S 19: Interactive 3D schematic model of drawing (not to scale)

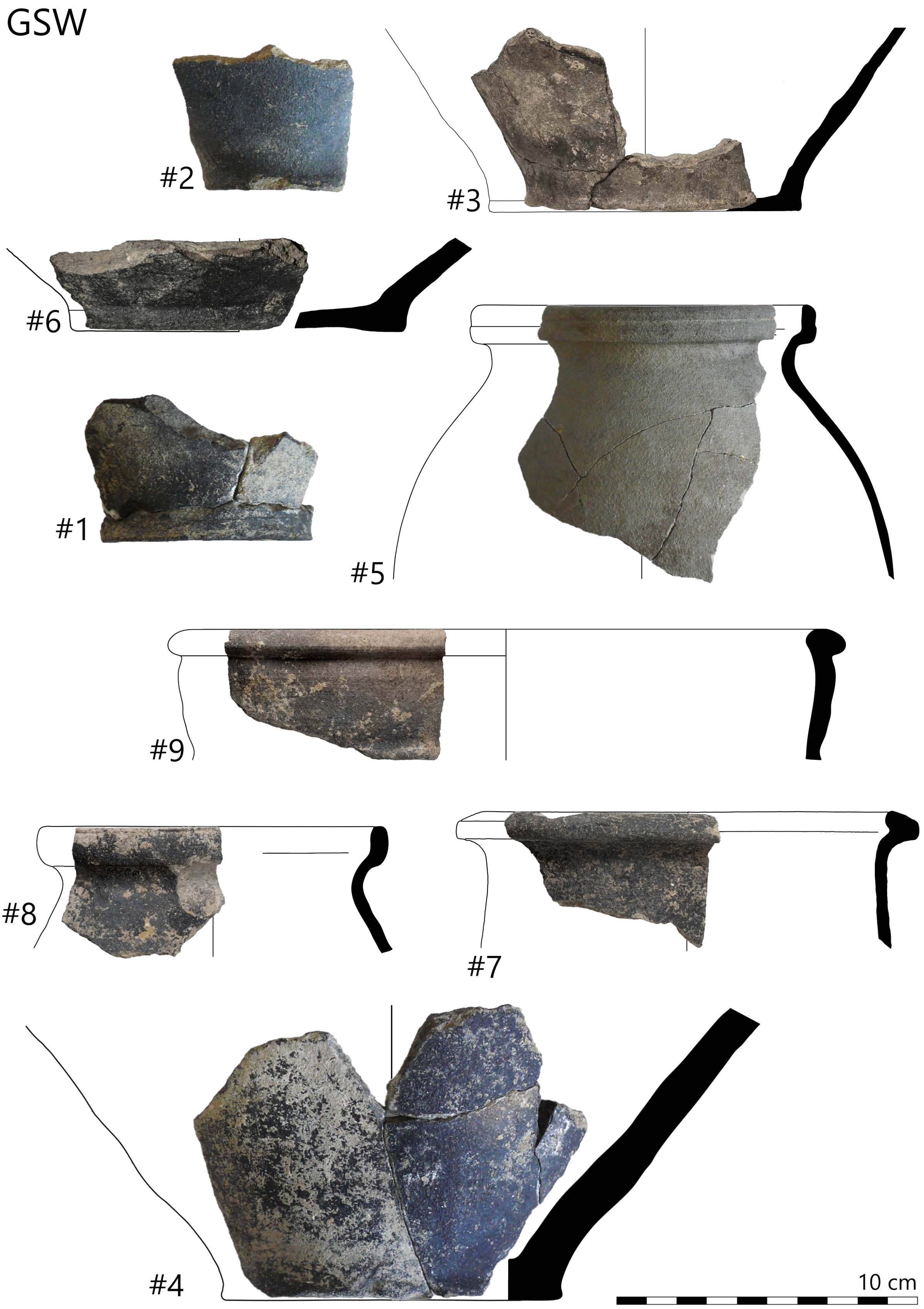

Supplementary Material A Figure S 20: 'Grey Grainy Surface Ware' archaeological pottery sherds. See Supplementary Material B: Table S5 for details.

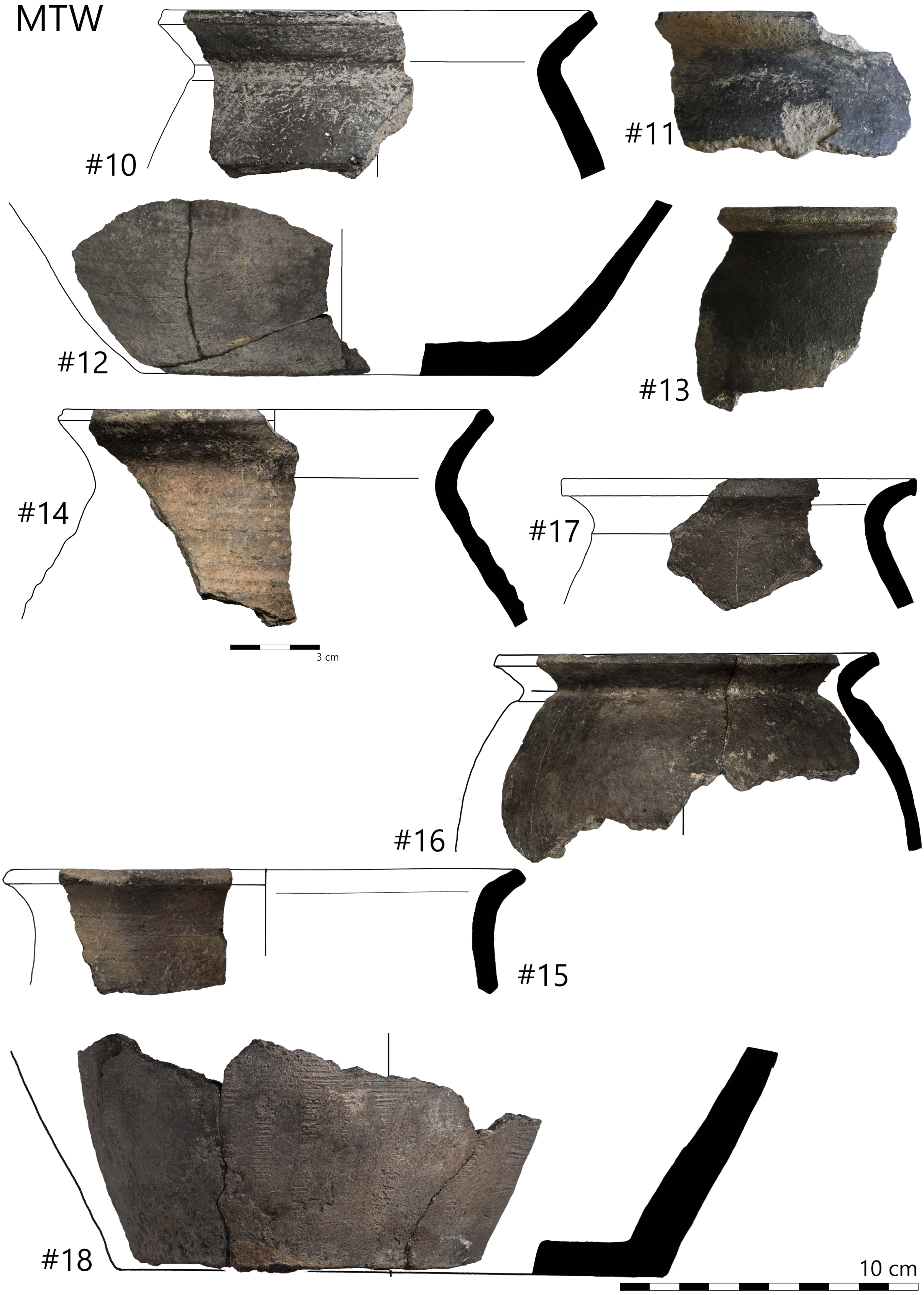

Supplementary Material A Figure S 21: 'Marble Tempered Ware' archaeological pottery sherds. See Supplementary Material B: Table S5 for details.

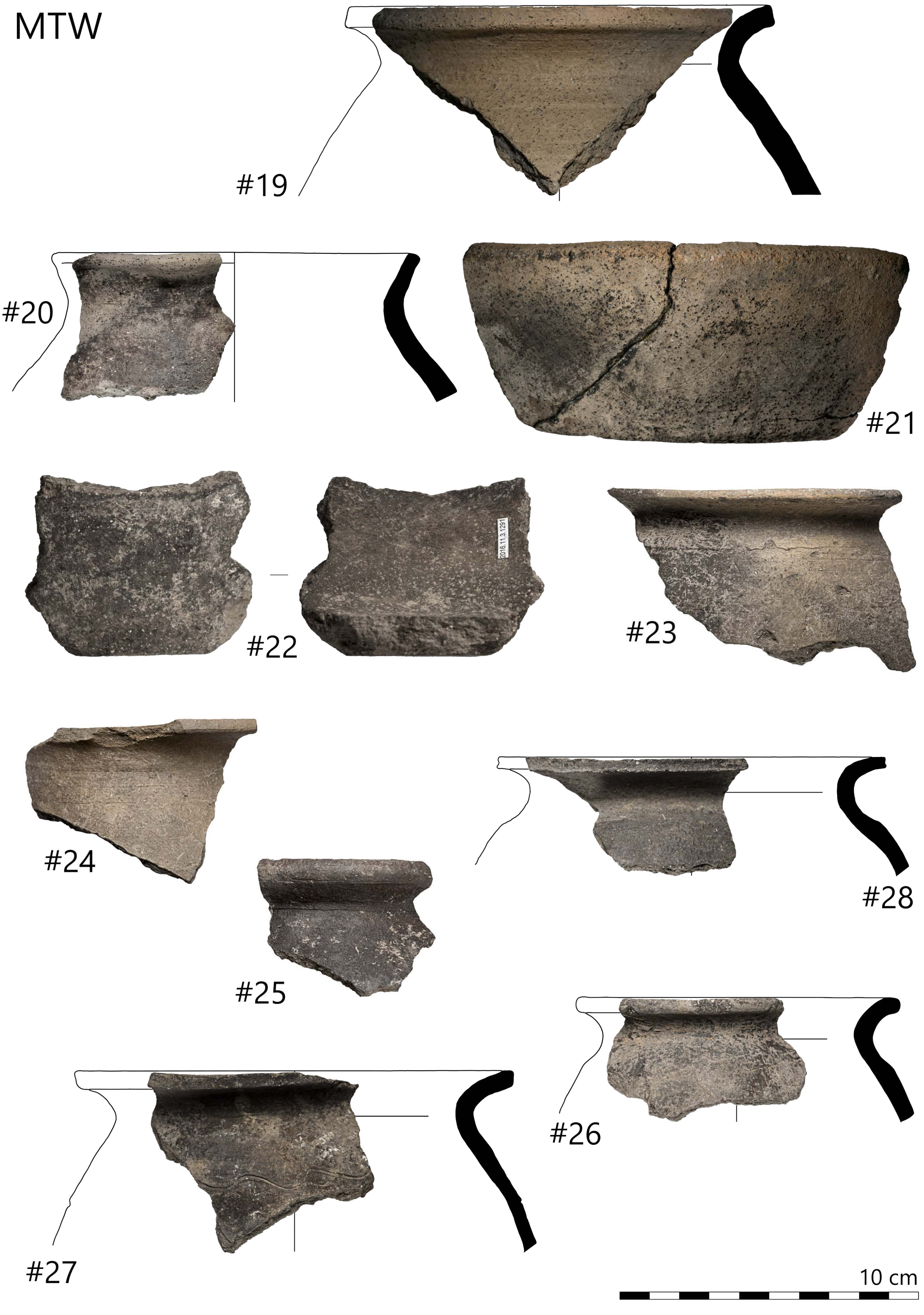

Supplementary Material A Figure S 22: ‘Marble Tempered Ware’ archaeological pottery sherds. See Supplementary Material B: Table S5 for details.

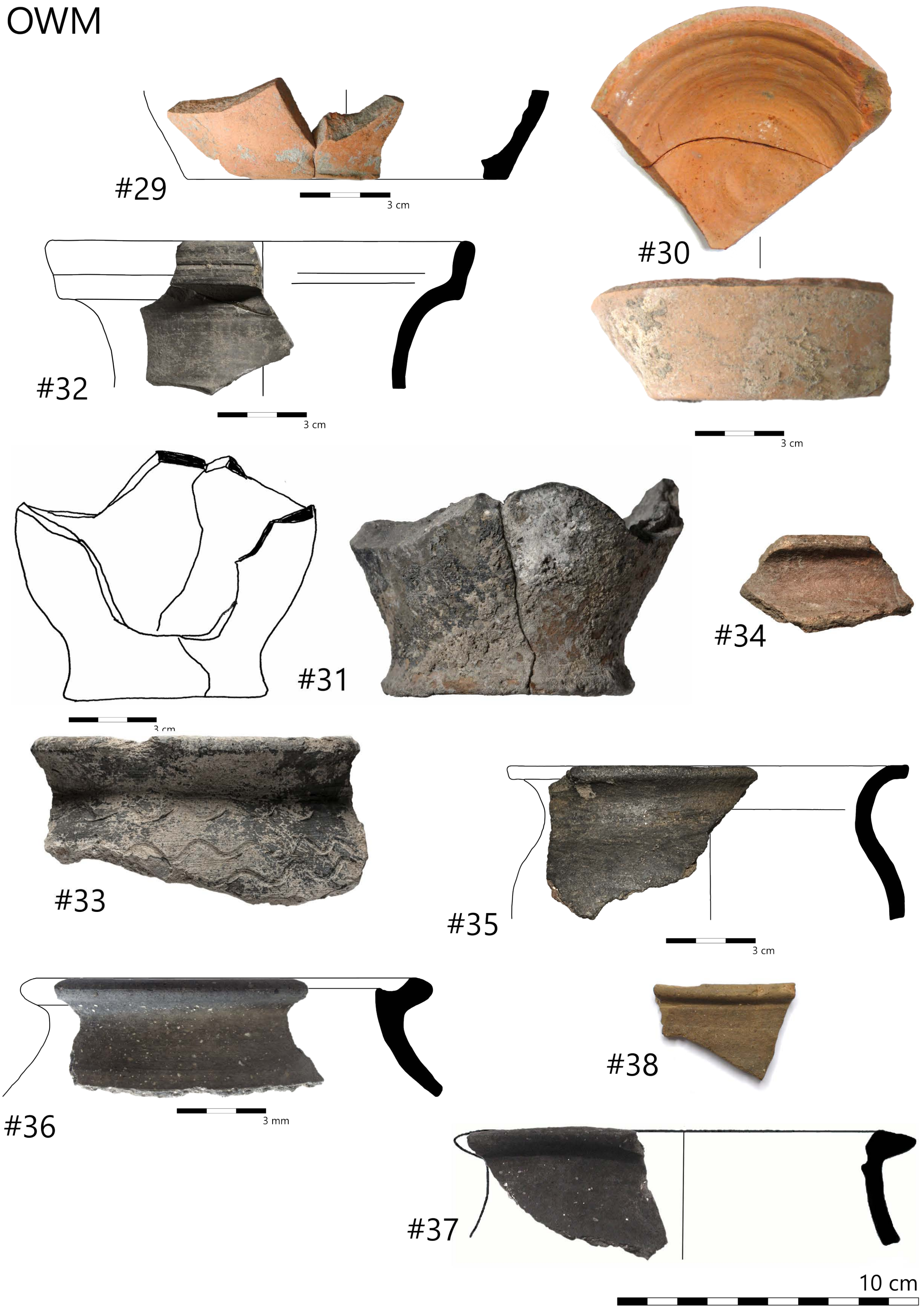

Supplementary Material A Figure S 23: ‘Other Wheel-Made’ archaeological pottery sherds. See Supplementary Material B: Table S5 for details.

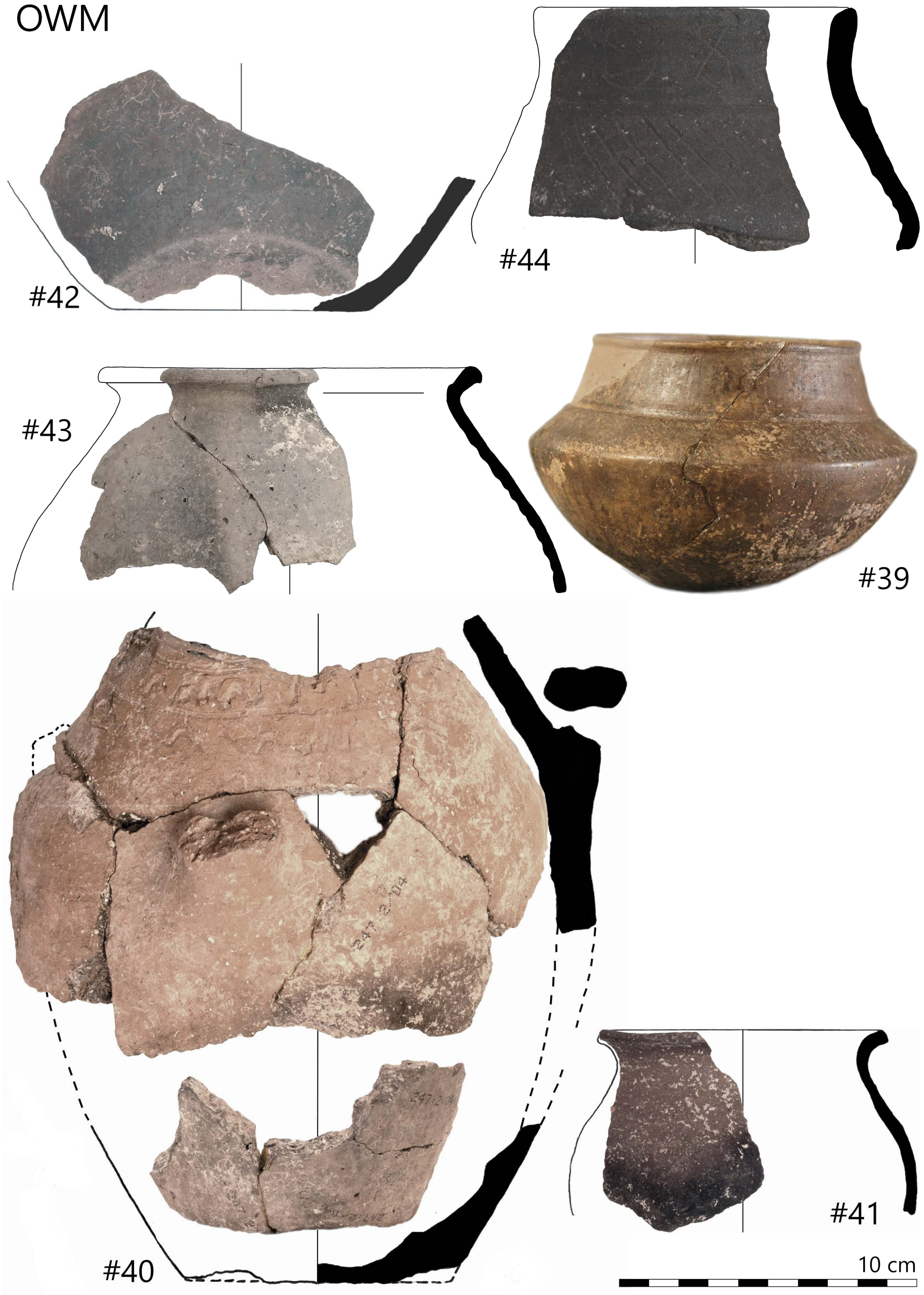

Supplementary Material A Figure S 24: ‘Other Wheel-Made’ archaeological pottery sherds. See Supplementary Material B: Table S5 for details.

OHB

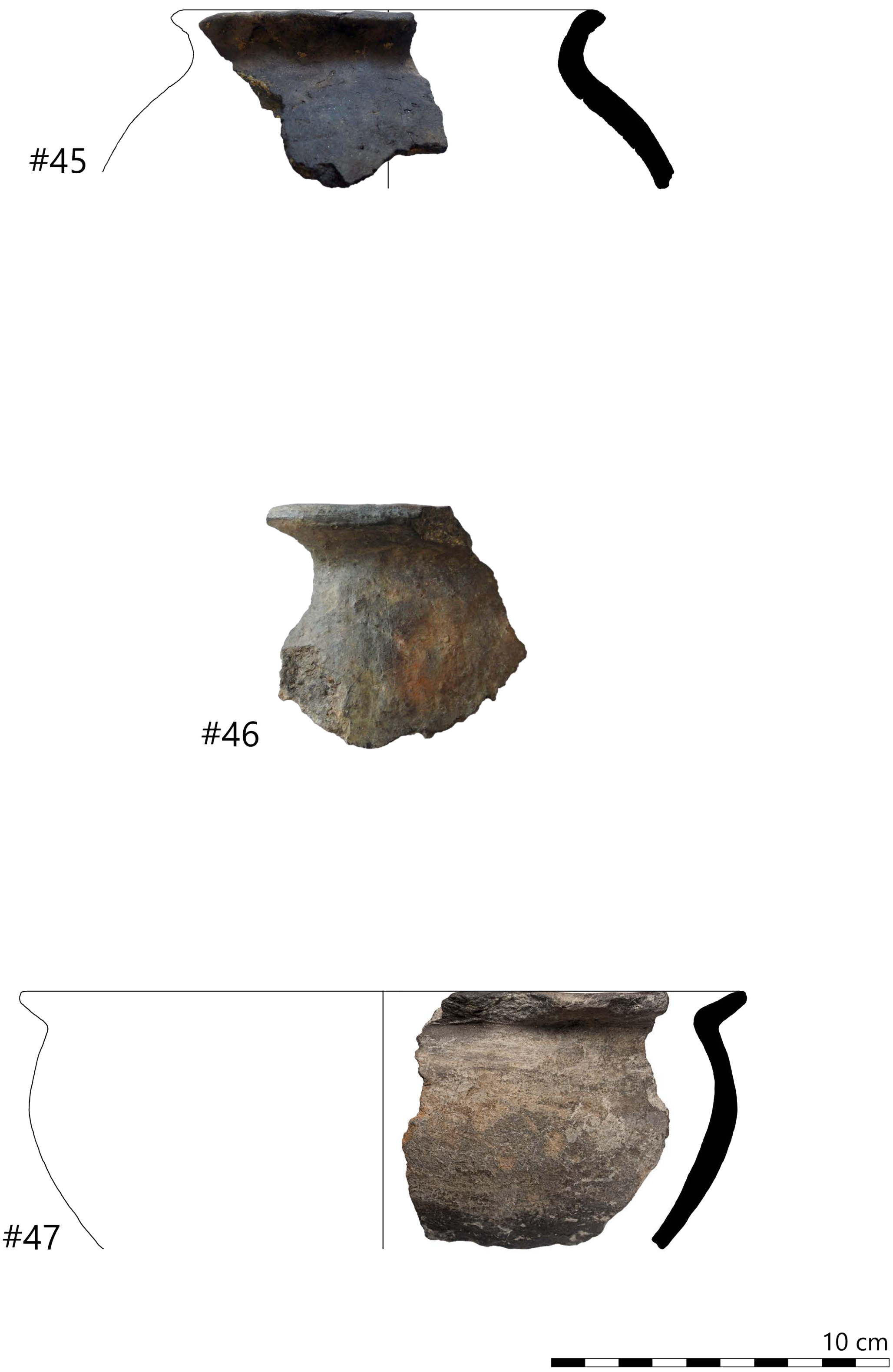

Supplementary Material A Figure S 25: ‘Other Hand-built’ archaeological pottery sherds. See Supplementary Material B: Table S5 for details.

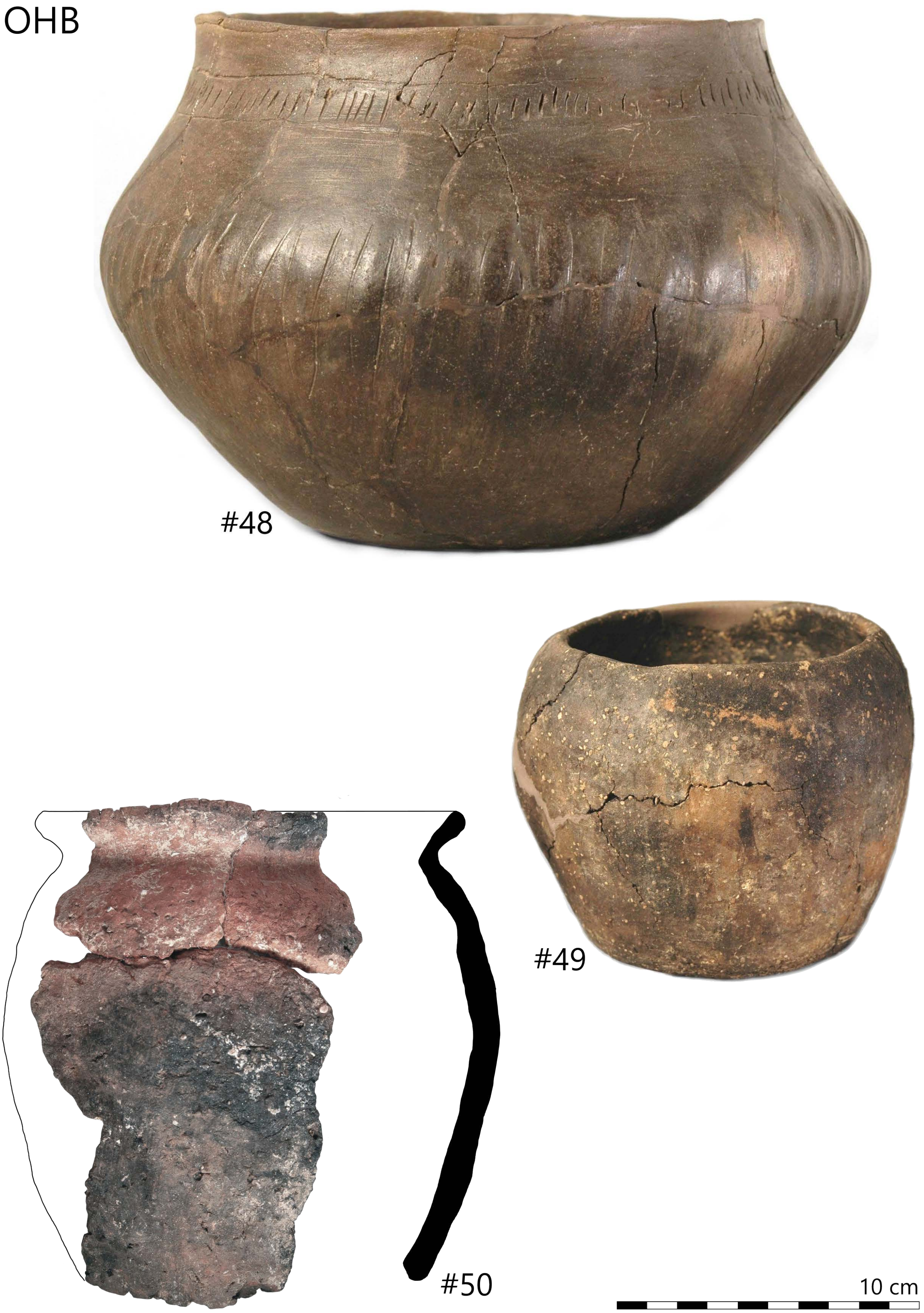

Supplementary Material A Figure S 26: 'Other Hand-built' archaeological pottery sherds. See Supplementary Material B: Table S5 for details.

References:

1. Keiderling, U. The new 'BerSANS-PC' software for reduction and treatment of small angle neutron scattering data. *Applied Physics A: Materials Science & Processing* **74**, s1455–s1457 (2002).
2. Hugot, N. Data processing for small angle neutron scattering analysis of archaeological samples: Research internship report, HUN-REN Centre for Energy Research, Budapest & ENSTA, Paris. https://bnc.hu/ys-sans/. (2022).
3. Pépy, G. New two-dimensional data treatment software for small-angle scattering. *J Appl Crystallogr* **40**, s433–s438 (2007).
4. Gait, J. *et al.* Quantitative 3D orientation analysis of particles and voids to differentiate hand-built pottery forming techniques using X-ray microtomography and neutron tomography. *Archaeol Anthropol Sci* **14**, 223 (2022).
5. Fazeli Nashali, H., Vidale, M., Bianchetti, P., Guida, G. & Coningham, R. The evolution of ceramic manufacturing technology during the Late Neolithic and Transitional Chalcolithic periods at Tepe Pardis, Iran. in 87–112 (Deutsches Archäologisches Institut, Eurasien-Abteilung Außenstelle Teheran, Berlin, 2010).
6. Roux, V. & Courty, M.-A. Identification of Wheel-fashioning Methods: Technological Analysis of 4th–3rdMillenniumBCOriental Ceramics. *Journal of Archaeological Science* **25**, 747–763 (1998).
7. Roux, V. & De Miroschedji, P. Revisiting the History of the Potter's Wheel in the Southern Levant. *Levant* **41**, 155–173 (2009).
8. Rye, O. S. *Pottery Technology: Principles and Reconstruction*. (Taraxacum, Washington, D.C, 1981).
9. Sterner, J. & David, N. Action on matter: the history of the uniquely African tamper and concave anvil pot-forming technique. *J African Arch* **1**, 3–35 (2003).
10. Berg, I. The potter's wheel in Mycenaean Greece: A re-assessment. in *Φιλική Συναυλία: Studies in Mediterranean Archaeology for Mario Benzi* (eds. Graziadio, G., Guglielmino, R., Lenuzza, V. & Vitale, S.) 113–121 (Archaeopress, Oxford, 2013).
11. Vidale, M. The onset of wheel-throwing in Middle Asia. A Neolithic innovation? in *Detecting and explaining technological innovation in prehistory* (eds. Spataro, M. & Furholt, M.) 199–218 (Sidestone Press, Leiden, 2020).
12. Heinrich-Tamáska, O. Castra and towns in the hinterland of the limes during Late Antiquity: Pannonia and the provinces along the Lower Danube in comparison. *Acta Archaeologica Carpathica* **LII**, 83–108 (2017).
13. Heinrich-Tamáska, O. & Prien, R. Castrum Virtuale: Rekonstruktion eines spätantiken Fundorts am Plattensee. Eine Ausstellung in Gedenken an Prof. Géza Alföldy. *(Universitätsmuseum Heidelberg – Kataloge* (2019) doi:10.11588/HEIBOOKS.480.

14. Heinrich-Tamáska, O. & Prien, R. Keszthely-Fenékpuszta in der Spätantike: Ein Vorbericht über die deutsch-ungarischen Ausgrabungen zwischen 2009 und 2017. *Antaeus, Communicationes ex Instituto Archaeologico Academiae Scientiarum Hungariae* **35–36**, 119–145 (2018).

15. Heinrich-Tamáska, O. Das spätrömische Innenbefestigung von Keszthely-Fenékpuszta: Innere Chronologie und funktioneller Wandel. in *Keszthely-Fenékpuszta im Kontext spätantiker Kontinuitätsforschung zwischen Noricum und Moesia* (ed. Heinrich-Tamáska, O.) 653–702 (Geisteswissenschaftlichen Zentrums Geschichte und Kultur Ostmitteleuropas e.V. und der Deutschen Forschungsgemeinschaft, Budapest, Leipzig, Keszthely, Rahden/Westf., 2011).

16. Blay, A. Überlegungen zur Bedeutung und Gültigkeit des Begriffs „Keszthely-Kultur“ und weitere mögliche Ansätze. *Antaeus* **35–36**, 167–186 (2017).

17. Heinrich-Tamáska, O. Archäologische Forschungen in Keszthely-Fenékpuszta: eine Einführung. in *Keszthely-Fenékpuszta: Katalog der Befunde und ausgewählter Funde sowie neue Forschungsergebnisse* (ed. Heinrich-Tamáska, O.) 9–23 (Archäologisches Institut der Ungarischen Akademie der Wissenschaften Geisteswissenschaftliches Zentrum Geschichte und Kultur Ostmitteleuropas e. V. Balatoni Museum, Budapest, Leipzig, Keszthely, Rahden/Westf., 2013).

18. Horváth, F. Das spätantike Keramikspektrum in Keszthely-Fenékpuszta – erste Ergebnisse. in *Keszthely-Fenékpuszta im Kontext spätantiker Kontinuitätsforschung zwischen Noricum und Moesia* (ed. Heinrich-Tamáska, O.) 597–652 (Geisteswissenschaftlichen Zentrums Geschichte und Kultur Ostmitteleuropas e.V. und der Deutschen Forschungsgemeinschaft, Budapest, Leipzig, Keszthely, Rahden/Westf., 2011).

19. Horváth, F. Römerzeitliche Keramik aus ausgewählten Befunden der Ausgrabungen bis 2002 in Keszthely-Fenékpuszta. in *Keszthely-Fenékpuszta: Katalog der Befunde und ausgewählter Funde sowie neue Forschungsergebnisse* (ed. Heinrich-Tamáska, O.) 397–442 (Archäologisches Institut der Ungarischen Akademie der Wissenschaften / Geisteswissenschaftliches Zentrum Geschichte und Kultur Ostmitteleuropas e. V. / Balatoni Museum, Budapest, Leipzig, Keszthely, Rahden/Westf., 2013).

20. Horváth, F. Glazed pottery of Keszthely-Fenékpuszta in the spectrum oft the ceramics of the Late Roman fortress. in *Late Roman Glazed Pottery in Carlino and in Central-East Europe. Proceedings of the Second International Meeting of Archaeology in Carlino (March 2009)* (eds. Magrini, Chiara & Sbarra, F.) 93–102 (BAR Publishing, Oxford, 2010).

21. Kulcsár, G. Ordacsehi-Kis-töltés. in *Gördülő Idő. Régészeti feltárások az M7-es autópálya Somogy megyei szakaszán Zamárdi és Ordacsehi között* (eds. Belényesy, K., Honti, S. & Kiss, V.) 185–192 (Somogy Megyei Múzeumok Igazgatósága & MTA Régészeti Intézete, Kaposvár & Budapest, 2007).

22. Bocsi, Z. 5. századi bronzműves műhely maradványai és késő római ékvéséssel díszített katonai övveret Ordacsehi–Kis-töltésről. in *Firkák III. Fiatal Római Koros Kutatók III. konferenciakötete* (ed. Balázs, P.) 13–26 (Savaria Megyei Hatókörű Városi Múzeum, Szombathely, 2014).

23. Bocsi, Z. Az Ordacsehi és Zamárdi V–VI. századi telepek, különös tekintettel a kerámia leletanyagra. (Eötvös Loránd Tudományegyetem, Budapest, 2007).

24. Bocsi, Z. Die Keramik aus zwei spätantiken Siedlungen am Balaton: Ordacsehi-Kis-töltés und Zamárdi-Kútvölgyi-dűlő, Komitat Somogy, Ungarn. in *Kulturwandel in Mitteleuropa.*

Langobarden, Awaren, Slawen. Akten der Internationalen Tagung in Bonn vom 25. bis 28. Februar 2008 (eds. Bemmann, J. & Schmauder, M.) 415–430 (R. Habelt, Bonn, 2008).

25. Pánczél-Bajnok, K., Pánczél, P., Szakmány, G. & Vida, T. 5-6. századi, Pannonia területéről származó kerámiák archeometriai elemzése / Archeometric analysis of 5–6th century ceramics from Pannonia. *Archeometriai Műhely* **XI**, 1–12 (2014).
26. Bocsi, Z., Gallina, Z. & Somogyi, K. Késő római - V. századi településrészlet Ordacsehi-Csereföldön. in *Beatus homo qui invenit sapientiam. Ünnepi kötet Tomka Péter 75. születésnapjára* (eds. Csécs, T. & Takács, M.) 93–115 (Lekri Group Kft., Győr, 2016).
27. Kiss, V. Zamárdi-Kútvölgyi-dűlő. in *Gördülő Idő. Régészeti feltárások az M7-es autópálya Somogy megyei szakaszán Zamárdi és Ordacsehi között* (eds. Belényesy, K., Honti, S. & Kiss, V.) 63–71 (Somogy Megyei Múzeumok Igazgatósága & MTA Régészeti Intézete, Kaposvár & Budapest, 2007).
28. Gál, E., Bartosiewicz, L. & Kiss, V. A fifth–sixth century CE lynx (Lynx lynx L., 1758) skeleton from Hungary: Cranial morphology and zoological interpretations. *Intl J of Osteoarchaeology* **32**, 783–791 (2022).
29. Bocsi, Z. Néhány ritka és jellemző edényfajta Zamárdi-Kútvölgyi-dűlőről. in *Hadak útján: a népvándorlás kor kutatóinak XIX. konferenciája, Xántus János Múzeum, Győr 2008. október 20-22* (eds. Bíró, S. & Tomka, P.) 111–124 (Győr-Moson-Sopron Megyei Múzeumok Igazgatósága, Győr, 2011).
30. Gál, E., Bartosiewicz, L., Kiss, V., Horváth, F. & Melis, E. A fifth- to sixth-century CE lynx (*Lynx lynx* L., 1758) skeleton from Hungary 2: Stature and archaeological interpretations. *Intl J of Osteoarchaeology* **34**, e3289 (2024).
31. Freeden, U. von & Vida, T. Ausgrabung des langobardzeitlichen Gräberfeldes von Szólád, Komitat Somogy, Ungarn. Vorbericht und Überblick über langobardzeitliche Besiedlung am Plattensee. Mit einem Exkurs von Péter Skriba. *Germania* **85**, 359-384. (2007).
32. Freeden, U. von. Ausgewählte Befunde aus dem langobardenzeitlichen Gräberfeld von Szólád, Komitat Somogy. in *Kulturwandel in Mitteleuropa Langobarden – Awaren – Slawen. Akten der Internationalen Tagung in Bonn vom 25. bis 28. Februar 2008* (eds. Bemmann, J. & Schmauder, M.) 399-413. (Deutsches Archäologisches Institut. Römisch-Germanische Kommission, Rheinisches Landesmuseum Bonn, Universität Bonn. Institut für Vor- und Frühgeschichtliche Archäologie, 2008).
33. Vida, T. *et al.* A multidisciplinary study on the Langobard period cemetery of Szólád in Pannonia. *Hungarian Archaeology* 46–62 (2017).
34. Alt, K. W. *et al.* Lombards on the Move – An Integrative Study of the Migration Period Cemetery at Szólád, Hungary. *PLoS ONE* **9**, e110793 (2014).
35. Amorim, C. E. G. *et al.* Understanding 6th-century barbarian social organization and migration through paleogenomics. *Nat Commun* **9**, 3547 (2018).
36. Bajnok, K. *et al.* Integrated petrographic and geochemical analysis of the Langobard age pottery of Szólád, Western Hungary. *Archaeol Anthropol Sci* **14**, 13 (2022).

37. Pap, I. K. Szeleste, Vasút mellett, Almás-alja II. (KÖH 67187). *Régészeti Kutatások Magyarországon* **2013**, 117–118 (2019).

38. Pap, I. K. Savaria keleti temetője és a szelestei germán temető épített és tegulás sírjai. *Savaria – A Vas Megyei Múzeumok értesítője* **38**, 91–105 (2016).

39. Tóth, G. A. & Pap, I. K. Germán temető a Nyugat-Dunántúlon / German cemetery in west Pannon area. in *Trendek és eredmények a biológiai kutatás és oktatás terén* (eds. Nagy, M. & Porácová, J.) 22–27 (Selye János Egyetem Tanárképző Kara, Komarno, 2016).
